# A Remote Nutritional Intervention to Change the Dietary Habits of Patients Undergoing Ablation of Atrial Fibrillation: Randomized Controlled Trial

**DOI:** 10.2196/21436

**Published:** 2020-12-07

**Authors:** Leticia Goni, Víctor de la O, M Teresa Barrio-López, Pablo Ramos, Luis Tercedor, Jose Luis Ibañez-Criado, Eduardo Castellanos, Alicia Ibañez Criado, Rosa Macias Ruiz, Ignacio García-Bolao, Jesus Almendral, Miguel Ángel Martínez-González, Miguel Ruiz-Canela

**Affiliations:** 1 Department of Preventive Medicine and Public Health University of Navarra Instituto de Investigación Sanitaria de Navarra Pamplona Spain; 2 Fisiopatología de la Obesidad y Nutrición Centro de Investigación Biomédica en Red Instituto de Salud Carlos III Madrid Spain; 3 Electrophysiology Laboratory and Arrhythmia Unit Hospital Montepríncipe, Grupo HM Hospitales University CEU-San Pablo Madrid Spain; 4 Arrhythmia Unit Department of Cardiology and Cardiac Surgery Clínica Universidad de Navarra Pamplona Spain; 5 Department of Cardiology Virgen de las Nieves University Hospital Granada Spain; 6 Biosanitary Research Institute of Granada (ibs.GRANADA) Granada Spain; 7 Arrhythmia Unit, Cardiology Service Alicante Institute of Health and Biomedical Research (ISABIAL-FISABIO Foundation) University General Hospital of Alicante Alicante Spain; 8 Department of Nutrition, Harvard TH Chan School of Public Health Harvard University Boston, MA United States

**Keywords:** atrial fibrillation, secondary prevention, remote intervention, Mediterranean diet, olive oil

## Abstract

**Background:**

The Prevention With Mediterranean Diet (PREDIMED) trial supported the effectiveness of a nutritional intervention conducted by a dietitian to prevent cardiovascular disease. However, the effect of a remote intervention to follow the Mediterranean diet has been less explored.

**Objective:**

This study aims to assess the effectiveness of a remotely provided Mediterranean diet–based nutritional intervention in obtaining favorable dietary changes in the context of a secondary prevention trial of atrial fibrillation (AF).

**Methods:**

The PREvention of recurrent arrhythmias with Mediterranean diet (PREDIMAR) study is a 2-year multicenter, randomized, controlled, single-blinded trial to assess the effect of the Mediterranean diet enriched with extra virgin olive oil (EVOO) on the prevention of atrial tachyarrhythmia recurrence after catheter ablation. Participants in sinus rhythm after ablation were randomly assigned to an intervention group (Mediterranean diet enriched with EVOO) or a control group (usual clinical care). The remote nutritional intervention included phone contacts (1 per 3 months) and web-based interventions with provision of dietary recommendations, and participants had access to a web page, a mobile app, and printed resources. The information is divided into 6 areas: *Recommended foods*, *Menus*, *News and Online resources*, *Practical tips*, *Mediterranean diet classroom*, and *Your personal experience*. At baseline and at 1-year and 2-year follow-up, the 14-item Mediterranean Diet Adherence Screener (MEDAS) questionnaire and a semiquantitative food frequency questionnaire were collected by a dietitian by phone.

**Results:**

A total of 720 subjects were randomized (365 to the intervention group, 355 to the control group). Up to September 2020, 560 subjects completed the first year (560/574, retention rate 95.6%) and 304 completed the second year (304/322, retention rate 94.4%) of the intervention. After 24 months of follow-up, increased adherence to the Mediterranean diet was observed in both groups, but the improvement was significantly higher in the intervention group than in the control group (net between-group difference: 1.8 points in the MEDAS questionnaire (95% CI 1.4-2.2; *P*<.001). Compared with the control group, the Mediterranean diet intervention group showed a significant increase in the consumption of fruits (*P*<.001), olive oil (*P*<.001), whole grain cereals (*P*=.002), pulses (*P*<.001), nuts (*P*<.001), white fish (*P*<.001), fatty fish (*P*<.001), and white meat (*P*=.007), and a significant reduction in refined cereals (*P*<.001), red and processed meat (*P*<.001), and sweets (*P*<.001) at 2 years of intervention. In terms of nutrients, the intervention group significantly increased their intake of omega-3 (*P*<.001) and fiber (*P*<.001), and they decreased their intake of carbohydrates (*P*=.02) and saturated fatty acids (*P*<.001) compared with the control group.

**Conclusions:**

The remote nutritional intervention using a website and phone calls seems to be effective in increasing adherence to the Mediterranean diet pattern among AF patients treated with catheter ablation.

**Trial Registration:**

ClinicalTrials.gov NCT03053843; https://www.clinicaltrials.gov/ct2/show/NCT03053843

## Introduction

### Atrial Fibrillation

Atrial fibrillation (AF) is currently the most common cardiac arrhythmia. In 2010, AF affected more than 33.5 million persons worldwide (20.9 million men and 12.6 million women) [1] and by 2030, 14 to 17 million people in Europe, and 12.1 million people in the United States will be diagnosed with AF [2,3]. This increased number of incident cases and age-adjusted prevalence of AF over the last few decades has led to a substantial disease and economic burden [1,4]. Currently, there are effective rhythm control strategies, such as catheter ablation, to recover sinus rhythm in patients with AF [5]. However, relapses occur in approximately 30% of the patients 1 year after the intervention [6]. A recent study showed that about 1 in 8 patients treated with catheter ablation needs to undergo a second procedure [7]. Therefore, research is needed to identify modifiable risk factors for the recurrence of AF to prevent recurrence and to maximize the durability of the sinus rhythm after ablation [8].

### The Mediterranean Diet and AF

The Mediterranean diet is considered an ideal nutritional model for cardiovascular health [9]. However, data on the relationship between the Mediterranean diet and AF are limited. The Prevention With Mediterranean Diet (PREDIMED) trial, in primary prevention, showed a protective effect of a Mediterranean diet enriched with extra virgin olive oil (EVOO) on new-onset AF [10]. Previously, a short-term trial showed that a supplement of omega-3 fatty acids and vitamin antioxidants was associated with a lower probability of AF occurring after on-pump cardiac surgery [11]. A case-control study has also suggested that the Mediterranean diet and a high intake of antioxidants increased the probability of spontaneous conversion of AF [12]. Other cohort studies have analyzed the effect of some components of the Mediterranean diet, such as olive oil, red wine, fatty fish, and nuts, although with inconclusive results [13-16]. A higher consumption of coffee and chocolate is inversely associated with the risk of incident AF [17,18]. In summary, the anti-inflammatory and anti-oxidative effects of these foods seem to support the potential preventive effect of the Mediterranean diet on the development of AF [19].

As far as we know, no previous study has assessed the effect of a Mediterranean diet intervention on preventing recurrences of AF after ablation. The PREvention of recurrent arrhythmias with Mediterranean diet (PREDIMAR) study is an ongoing secondary prevention trial aimed at assessing the effect of a Mediterranean diet enriched with EVOO [20]. The novelty of this trial is that dietitians conduct the nutritional intervention remotely in coordination with the face-to-face intervention conducted by the cardiologists and health care team. Different studies have demonstrated that remote nutritional interventions (web page, mobile phone app, email, text messaging, and phone calls) are at least as effective as face-to-face interventions in terms of weight loss and in changing eating behaviors [21-23]. However, no previous study has assessed how a remote intervention may change the adherence to the Mediterranean diet in patients with AF after undergoing catheter ablation to recover sinus rhythm.

The principal objective of this study is to assess the effect of a web-based and telephone intervention in obtaining favorable dietary changes in the context of a secondary prevention trial of AF. In addition, we provide a detailed description of the remote nutritional intervention conducted in the PREDIMAR trial.

## Methods

### Overview of the PREDIMAR Study

The PREDIMAR study was a multicenter, randomized, controlled, single-blind trial. The study protocol has been described in detail elsewhere [20]. Briefly, the aim of the PREDIMAR study was to analyze the effect of a Mediterranean diet intervention enriched with EVOO on the prevention of atrial tachyarrhythmia recurrence after catheter ablation. Participants were recruited from 4 Spanish hospitals: Hospital Montepríncipe (Madrid), Clínica Universidad de Navarra (Pamplona), Hospital Virgen de las Nieves (Granada), and Hospital General Universitario (Alicante).

**Figure 1:**
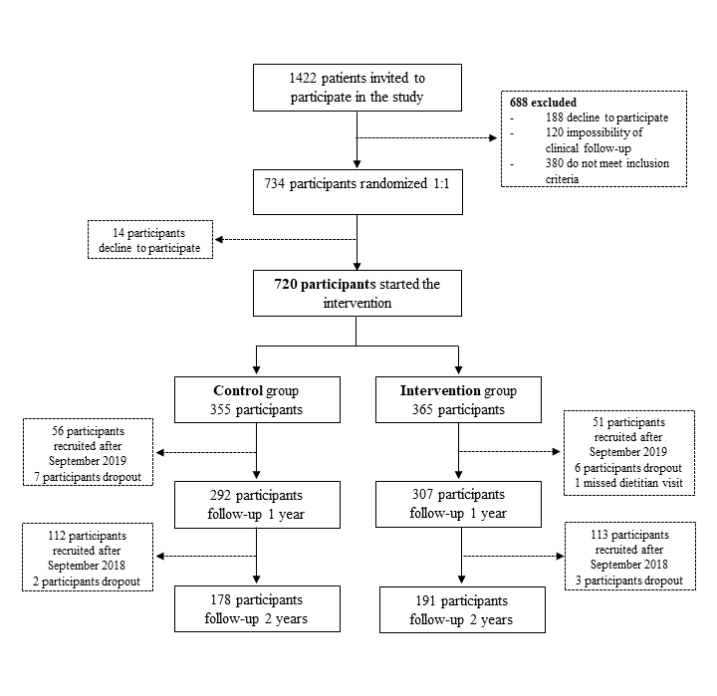
Flowchart of participant screening, recruitment, and randomization.

Between March 2017 and January 2020, 1422 patients were invited to participate in the study (Figure 1). Of them, although 734 patients were recruited and randomized, 720 started the intervention. The PREDIMAR trial is an ongoing study that will finish in January 2022. The flowchart shows participants who have completed 1- or 2-year follow-up with data updated to September 2020. Until this date, the number of dropouts was 18, and the retention rate was 95.6% among participants with follow-up over 12 months (560/574), and 94.4% with follow-up over 24 months (304/322).

After catheter ablation for the treatment of AF, all participants who gave their informed consent, and who fulfilled the inclusion criteria were randomly assigned to the intervention (Mediterranean diet enriched with EVOO) or the control group (usual clinical care). The intervention period took 2 years (Multimedia Appendix 1). During this time, clinical follow-up visits took place at 3, 6, 12, 18, and 24 months after the ablation. To determine possible atrial tachyarrhythmia recurrences, each patient received a portable cardiac rhythm-monitoring device on the third month visit, which was returned on the 18th-month visit. This was because recurrences that occur during the first 3 months after the ablation were not considered clinically relevant, as they could be due to the ablation and the healing process (so called blanking period) [5-7]. At baseline and at 12- and 24-month follow-up, information about lifestyle habits (including food habits, physical activity, and life quality) was recorded by a dietitian by phone. Blood and urine samples were also collected at baseline and at 12 and 24 months of the intervention period. Clinical care providers (cardiac electrophysiologists) were blinded to the group assignment.

The trial was registered at ClinicalTrials.gov NCT03053843. The Research Ethics Committees from each recruitment center approved the protocol. All participants provided written informed consent after they received the information sheet and additional verbal explanation of the study characteristics.

### Nutritional Intervention

The aim of the nutritional intervention of the PREDIMAR study is to improve adherence to the Mediterranean diet. The Mediterranean diet is characterized by the exclusive use of EVOO for all culinary purposes and high consumption of vegetables, fruits, whole grains, legumes, and nuts; moderate consumption of fish; and very low consumption of red and processed meats, refined grains, sweet desserts, and whole-fat dairy products (only fermented dairy products, cheese and yogurt, are consumed in moderation) and ultraprocessed foods [9]. Specifically, the dietary recommendations for the intervention group were the use of 4 or more spoons of EVOO for cooking and dressing of dishes; consumption of 2 or more servings (200 g per serving) per day of vegetables (at least one of them as salads); 3 or more servings (125 g per serving) per day of fruits (including natural juices); 3 or more servings (60-80 g per serving) per week of legumes; 3 or more servings (150 g per serving) per week of fish or seafood (at least one serving of fatty fish); 3 or more servings (30 g per serving) per week of nuts; selected white meats (ie, poultry without skin, rabbit) instead of red meats (ie, beef, pork) or processed meats (ie, sausages, burgers); regularly cooking (2 or more times per week) with salsa made with minced tomato, garlic, and onion simmered in olive oil (sofrito) for dressing different dishes; selected whole grain cereals (ie, bread, pasta, rice) instead of refined cereals; eliminate or limit the consumption of cream, butter, and margarine, carbonated and/or sweetened beverages, commercial bakery products (ie, sweet desserts, cakes, pastries, cookies), and ultraprocessed foods. These recommendations were discussed with participants during periodical phone interviews with a dietitian, and personalized goals were set for the participants according to their improvement needs. Nutritional intervention was not based on a specific amount of calories or a macronutrient distribution.

In the PREDIMAR study, we used a remote nutritional intervention rather than an in-person approach. This remote intervention was conducted by a team of registered dietitians by phone and on the internet, and participants had access to web-based information on a website, a mobile app, and printed material. The intervention was conducted by the same group of dietitians to all participants from the 4 centers. This characteristic allowed a highly homogeneous intervention according to the protocol of the study, but it also allowed for tailored nutrition education through personal interviews conducted by phone and web-based communication with the dietitians.

#### Web Page

A multidisciplinary group of dietitians-nutritionists, epidemiologists, medical doctors, and chefs developed the content of the website for this study, and professional multimedia programmers produced it (Nubba Group).

Once a participant was randomized, they received an automated email with a username and password to access the PREDIMAR website. Participants in the control group had access to general information about AF only.

For participants in the intervention group, the content of the website was divided into 6 areas: *recommended foods*, *menus*, *News and Online resources*, *practical tips*, *Mediterranean Diet classroom*, and *Your personal experience*. These resources were mainly focused on the Mediterranean diet, based in most cases on locally and seasonally available products, or looking for a new combination of healthy foods to increase the interest of participants in healthy food and cooking. This information was based on 2 of the 3 essential phases of nutrition education: the motivational phase, which aimed to increase awareness and enhance motivation (*why* to make changes), and the action phase, which aimed to facilitate the ability to take action (*how* to make changes) [24]. Moreover, participants could self-assess their level of adherence to the Mediterranean diet through the 14-item Mediterranean Diet Adherence Screener (MEDAS) questionnaire (Multimedia Appendix 2) [25]. Multimedia Appendix 3 provided screenshots of the web page.

The information of the different areas of the web page was updated every week and was sequentially and automatically activated according to the number of weeks that each participant had been followed up in the study.

*Recommended foods* encompassed a total of 53 typical foods from the Mediterranean diet. Each factsheet provided an overview of the food, including the definition, the portion size, the recommended frequency of consumption, the nutritional value, health benefits, examples of how to include it in the diet, and 2 simple and delicious recipes.

The first entry of the *Menus* area was a week eating plan adapted to achieve the Mediterranean dietary pattern and a table that specified the recommended frequency of consumption for each group of food, which foods were included in the group, and the portion size. The following entries, 26 menus, provided examples to follow the eating guidelines outlined in the week eating plan. In general, each menu comprised 8 recipes based on crude and cooked vegetables, 3 recipes based on legumes, 3 recipes based on whole cereals, 3 recipes based on lean meats, 7 recipes based on fish, and 4 recipes based on eggs. Some menus specifically focused on a Mediterranean breakfast, healthy snacks, desserts based on fruit, and homemade healthy takeaway, among others. Each menu focused on one objective of the Mediterranean diet pattern and included a shopping list for one person for a week.

The area *News and Online resources* included a total of 71 news items and 7 web pages for the general public. In each news item, we provided our opinion, based on scientific evidence, about news regarding health and diet, taking into account that sometimes the information is confusing for the general population. The web pages were also related to diet and health and included blogs and web-based tools to increase adherence to the Mediterranean diet.

*Practical tips* were developed to facilitate the ability to dietary change. The tips used graphic images to calculate the hand-based portion size of different food groups, identify the seasonality of fruits and vegetables, guide healthy food shopping, guide how to eat healthy food in a restaurant, and identify the benefits of eating in family, among others. Recommendations using hand-based portion sizes were followed because they are considered to be an easy and acceptably accurate method for estimating portion sizes [26,27].

The *Mediterranean diet classroom* area consisted of videos about the theoretical and practical aspects of healthy nutrients, foods, and cooking. There were 24 videos related to theoretical aspects of nutrition, including information about nutrients (definition, classification, function, food source of the nutrient, and health effect) and food groups (definition, classification, health properties, portion size, and recommended frequency consumption). In addition, there were 12 videos with practical tips aimed to increase the adherence to the Mediterranean diet and 16 videos with novel but easily made recipes developed in collaboration with chefs of the Basque Culinary Center.

Finally, testimonials from volunteers of the PREDIMAR study were included in the *Your personal experience* area to motivate other volunteers to follow the Mediterranean diet.

#### Mobile App

The intervention program also included an Android and iPhone app that allowed participants to access the web page content in an easier manner (Multimedia Appendix 4). The mobile and tablet app was activated when 446 subjects (226 subjects in the intervention group) had begun the trial. Its availability was announced to all volunteers by email, and it included a pop-up on the web page and a paper sheet with the announcement in the print material modules.

#### Website and Mobile App Usage

Data on the frequency of access to the website and the mobile app were provided by the same web page to the dietitians, and this information was used to determine the level of engagement of each participant and to orient the intervention of the dietitian during the phone call with each participant during the follow-up. In these phone calls, participants were informed about the tools available on the web page and encouraged to use it every week.

Biweekly automated email notification was sent, announcing the updated information of each area according to the number of weeks that each participant had been followed up in the study.

#### Printed Material

Participants in the intervention group also received printed material across the time of the study. Once participants were assigned to the intervention group, they received a book about the traditional Mediterranean diet [28]. This book explains the main food habit objectives of the Mediterranean diet and provides more than 50 recipes.

Later in the first follow-up clinical visit (third month), participants in the intervention group received a binder with the first print module, which corresponds to the information of the first 8 weeks of the website. Subsequently, every 8 weeks, participants were sent 10 print modules with the information of the website.

A second book was provided to participants at the sixth month of the intervention [29]. This book shows the beneficial effects of healthy lifestyles, mainly focusing on the Mediterranean diet and its components.

Finally, in the 12th month clinical visit, a magnetic board was given to each volunteer of the intervention group. This board was a helpful tool to improve weekly eating plans.

#### Human Support (Phone Calls and Email)

The intervention began with a phone call from the dietitian once the patient had agreed to participate in the study and was randomly assigned to the intervention or control group. During this first phone call, the dietitian collected information about lifestyle, nutrition, and quality of life from all participants [25,30,31]. Those participants in the control group received only general information about the study, and no nutritional information was provided to them. Participants in the control group were informed that they would receive a second phone call after 1 year of follow-up to collect further information and a third phone call at the end of the study after 2 years of follow-up. They were informed that they would receive a gift when they visited their cardiologist at the end of the study. This gift was a book about the Mediterranean diet [28] and 3 L of EVOO, but they were not told this to minimize any nutritional intervention.

Every 3 months during the 2-year follow-up, participants in the intervention group were contacted by the dietitian by phone to complete the MEDAS questionnaire (Multimedia Appendix 2) and to conduct the nutritional education session [25]. The nutritional information collected in the food frequency questionnaire (FFQ) (Multimedia Appendix 5) and the updated information obtained with the MEDAS questionnaire were used by the dietitian to provide personalized nutritional information. The MEDAS questionnaire was designed to assess the level of compliance with the Mediterranean diet as well as to improve adherence to the Mediterranean diet [25]. Moreover, in each phone call, participants in the intervention group were asked about their use of the web page or mobile app and the printed material.

The aim of this tailored nutritional education was to increase the quality of the participants’ diet according to the traditional Mediterranean diet and to adapt these general recommendations to the personal food preferences and lifestyles of each participant. Thus, volunteers with excessive body weight, with diabetes, or with any other disease related to nutrition, received specific recommendations by the dietitian to avoid contradictory information from other care professionals because in some cases, the intake of some foods is limited.

On the basis of behavioral literature showing the importance of continued contact during intervention [32], a specific section was designed on the website in which participants could ask during the study any questions regarding diet and health topics. Later, participants’ questions were included anonymously in a *frequently asked questions* area.

#### EVOO

Each participant in the intervention group received 0.5 L of EVOO per week for free. The EVOO was provided at each clinical visit, and the aim was to encourage participants to consume at least four spoons of EVOO per day. As part of the Mediterranean diet intervention, participants were encouraged to use EVOO as the culinary fat in their homes.

### Dietary Assessment

Trained registered dietitians collected data on food habits during the phone calls at baseline and at years 1 and 2 of follow-up. Adherence to the traditional Mediterranean diet was appraised by a validated 14-item MEDAS questionnaire (Multimedia Appendix 2) [25]. In the intervention group, this tool was used to assess the level of compliance with the intervention and as a key element to guide the personalized motivational interviews during the follow-up study every 3 months. Dietary intake was analyzed by a 147-item semiquantitative FFQ validated for the Spanish population (Multimedia Appendix 5) [30]. The FFQ included 9 frequency options for a specified usual portion size (never or less than once a month, 1-3 times a month, once a week, 2-4 times a week, 5-6 times a week, once a day, 2-3 times a day, 4-6 times a day, and more than 6 times a day). Energy and nutrient intake were calculated from Spanish food composition tables [33,34]. For the present analysis, changes in food consumption were assessed for 20 food groups: fruits, vegetables, refined olive oil, virgin olive oil (VOO), other fats than olive oil, whole grain cereals, refined cereals, dairy products, pulses, nuts, white fish, fatty fish, white meat, red meat, eggs, sweets, red wine, other wines than red wine, beer, and other alcoholic drinks (liquors, distilled beverages); and 9 nutrients, carbohydrates, protein, total fat, monounsaturated fatty acids (MUFAs), polyunsaturated fatty acids (PUFAs), saturated fatty acids (SFAs), omega-3, dietary fiber, and sodium.

Information on the hydroxytyrosol content in foods was obtained from the Phenol-Explorer database. When an item of the FFQ included more than one food, we used a weighted average according to the typical relative frequency of consumption in the Spanish population [35]. If this information was not available, the consumption of the foods included in the same item was equally divided. For recipes, polyphenol content was calculated according to the ingredients. The retention factors from the Phenol-Explorer database were applied to consider food cooking and processing to calculate the hydroxytyrosol content.

### Other Measurements

At baseline, registered dietitians also collected information about sociodemographic characteristics (education level, civil status, and working status), smoking habit, and anthropometric measurements (self-reported weight and height). During this phone call, information about the physical activity was also collected with a physical activity questionnaire validated for the Spanish population [31].

In clinical visits, the cardiologists collected AF-related variables, complications related to catheter ablation, presence of concomitant chronic diseases, and changes in medication related to arrhythmia, among others. Electrocardiographic monitoring was performed at each visit [20].

### Statistical Analyses

For this study, we used the PREDIMAR database generated until September 2020, including 1- and 2-year follow-up data. Quantitative variables were expressed as means and SDs, whereas categorical variables were described as number and percentages (n [%]). The Student *t* test for continuous variables and the chi-square test for categorical variables were applied to test differences in baseline characteristics between the intervention groups. Mixed effects linear models were used to assess changes in nutritional variables from baseline to 12- and 24-month follow-up visits. A 2-level mixed linear model with random intercepts at the recruitment center and participant was fitted. The analyses were performed using STATA (v 12.0, StataCorp LP). The significance level (2-tailed) was set at *P* values lower than .05.

## Results

### Baseline Characteristics

Between March 2017 and January 2020, 720 patients started the intervention (Figure 1). Among them, 549 were men (76%) and 171 were women (24%), and the mean age was 59.7 years (SD 10.7). Table 1 shows the demographic, anthropometric, and lifestyle baseline characteristics of participants according to the randomized groups (intervention or control). No significant differences were found for age, sex, type of AF, BMI, educational level, civil and working status, smoking habit, physical activity, and protein intake between intervention groups. Meanwhile, significant differences were observed in dietary habits. Participants in the intervention group had a lower intake of energy and carbohydrates and a higher intake of fat than participants in the control group. Moreover, participants in the intervention group had a higher adherence to the Mediterranean diet. These differences could be because of social desirability bias, although the magnitude of the differences between groups was small, and they could be interpreted in the light of the large power and sample size of the study.

**Table 1:** Baseline characteristics of the participants in the prevention of recurrent arrythmias with Mediterranean diet trial.

Characteristics		Intervention (n=365)	Control (n=355)	*P* value
Age, years, mean (SD)		59.9 (10.5)	59.6 (10.9)	.77
**Sex, n (%)**				**.41**
	Men	283 (77.5)	266 (74.9)	
	Women	82 (22.5)	89 (25.1)	
**Type of AF^a^, n (%)**				**.94**
	Persistent	147 (40.3)	142 (40.0)	
	Paroxysmal	218 (59.7)	213 (60.0)	
BMI (kg/m^2^)		27.8 (4.0)	27.8 (4.3)	.92
**Education, n (%)**				**.47**
	Secondary or less	198 (54.2)	202 (56.9)	
	University	167 (45.7)	153 (43.1)	
**Civil status, n (%)**				**.63**
	Single	24 (6.6)	27 (7.6)	
	Married	278 (76.2)	275 (77.5)	
	Others	63 (17.3)	53 (14.9)	
**Working status, n (%)**				**.80**
	Working	199 (54.5)	191 (53.8)	
	Retired	139 (38.1)	133 (37.5)	
	Others	27 (7.4)	31 (8.7)	
**Smoking status, n (%)**				**.39**
	Never	139 (38.1)	121 (34.1)	
	Former	206 (56.4)	208 (59.6)	
	Current	20 (5.5)	26 (7.3)	
Physical activity (MET^b^-hours/week)		33.4 (20.7)	34.1 (22.1)	.67
MEDAS^c^ score (14 items)		7.8 (2.0)	7.2 (2.0)	<.001
Energy intake (kcal/day)		2396 (670)	2527 (813)	.01
Carbohydrate intake g/day)		251.5 (5.2)	275.7 (5.6)	.002
Protein intake (g/day)		94.3 (1.3)	99.9 (1.4)	.002
Fat intake (g/day)		104.8 (1.5)	107.6 (2.0)	.21

^a^AF: atrial fibrillation.

^b^MET: metabolic equivalent.

^c^MEDAS: Mediterranean Diet Adherence Screener.

### Mediterranean Diet Adherence

**Figure 2:**
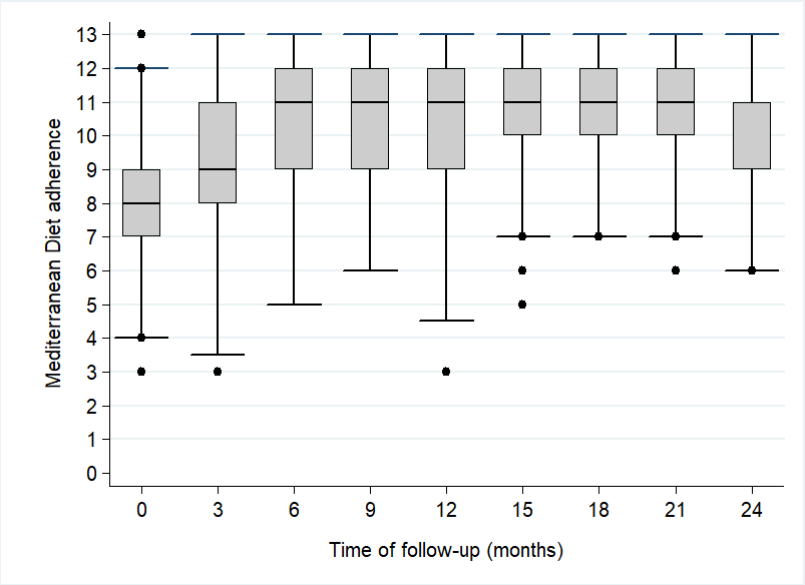
Adherence to the Mediterranean diet among participants in the intervention group in each follow-up phone call.

After 12 and 24 months of follow-up, an increase in the adherence to the Mediterranean diet was observed in both groups (Multimedia Appendix 6). The mean (95% CI) MEDAS score was 7.8 (7.6-8.0) at baseline, 10.2 (10.0-10.4) at 12 months (increase, 2.4 [2.2-2.6]) and 10.2 (10.0-10.4) at 24 months (increase, 2.4 [2.1-2.6]) in the intervention group; and 7.2 (7.0-7.4) at baseline, 7.5 (7.2-7.7) at 12 months (increase, 0.3 [0.03-0.5]), and 7.9 (7.6-8.2) at 24 months (increase, 0.6 [0.4-0.9]) in the control group. Thus, the increase in Mediterranean diet adherence was higher in the intervention than in the control group at 12 months (between-group difference 2.1, 95% CI 1.8-2.4, *P*<.001) and 24 months (between-group difference 1.8, 95% CI 1.4-2.1; *P*<.001) of follow-up. Figure 2 shows the adherence to the Mediterranean diet for each 3-month follow-up visit among participants of the intervention group. The median score of the adherence to the Mediterranean diet increased gradually until the 6-month follow-up visit. After that, the median adherence was maintained until the 21-month follow-up phone call.

### Food Group Consumption

As intended, the Mediterranean diet intervention group showed a significant improvement in the consumption of vegetables, fruits, whole grain cereals, pulses, nuts, white fish, fatty fish, white meat, and VOO compared with the control group at 1-year follow-up (Table 2). Moreover, the intervention group reduced the consumption of refined olive oil and unhealthy foods, refined cereals, red and processed meat, and sweets compared with the control group. No significant differences between groups were observed for the change in the consumption of dairy products, eggs, other fats than olive oil, wine, beer, and other alcoholic drinks.

**Table 2:** Baseline food groups consumption and changes by randomized treatment group at 12- and 24-month follow-up visits of participants in the prevention of recurrent arrythmias with Mediterranean diet trial.

Food groups				Group intervention				Between group difference^a^, mean (95% CI)		*P* value^b^
				Intervention, mean (95% CI)		Control, mean (95% CI)				
**Vegetables (g/day)**										
	Baseline		217.3 (208.1 to 226.5)		228.2 (217.3 to 239.1)		N/A^c^		N/A	
	**1 year**		**282.6 (269.2 to 295.9)**		**251.6 (237.7 to 265.5)**		**N/A**		**N/A**	
		1-year change		65.2 (52.1 to 78.4)		23.4 (9.8 to 37.0)		41.9 (23.0 to 60.8)		<.001
	**2 years**		**250.4 (238.3 to 262.5)**		**280.8 (265.0 to 296.7)**		**N/A**		**N/A**	
		2 years change		33.1 (19.8 to 46.3)		52.6 (36.7 to 68.6)		−19.5 (−40.3 to 1.2)		.06
**Fruits (g/day)**										
	Baseline		313.7 (293.2 to 334.2)		313.7 (291.6 to 335.9)		N/A		N/A	
	**1 year**		**464.9 (441.4 to 488.4)**		**327.6 (304.9 to 350.3)**		**N/A**		**N/A**	
		1-year change		151.2 (127.6 to 174.7)		13.9 (−6.8 to 34.6)		137.3 (105.9 to 168.7)		<.001
	**2 years**		**439.5 (414.6 to 464.3)**		**304.3 (274.8 to 333.7)**		**N/A**		**N/A**	
		2 years change		125.7 (99.8 to 151.7)		−9.5 (−40.3 to 21.3)		135.2 (94.9 to 175.5)		<.001
**Refined cereals (g/day)**										
	Baseline		122.8 (110.4 to 135.2)		131.8 (119.8 to 143.9)		N/A		N/A	
	**1 year**		**66.3 (57.8 to 74.8)**		**111.4 (99.9 to 122.9)**		**N/A**		**N/A**	
		1-year change		−56.5 (−67.8 to −45.2)		−20.4 (−32.0 to −8.8)		−36.1 (−52.3 to −19.9)		<.001
	**2 years**		**56.2 (46.8 to 65.6)**		**108.3 (94.8 to 121.8)**		**N/A**		**N/A**	
		2 years change		−66.6 (−80.6 to −52.6)		−23.5 (−38.1 to −9.0)		−43.0 (−63.2 to −22.9)		<.001
**Whole cereals (g/day)**										
	Baseline		40.3 (33.5 to 47.2)		36.2 (29.3 to 43.1)		N/A		N/A	
	**1 year**		**50.5 (43.7 to 57.3)**		**28.6 (22.7 to 34.4)**		**N/A**		**N/A**	
		1-year change		10.1 (1.9 to 18.4)		−7.7 (−15.3 to 0.00)		17.8 (6.5 to 29.1)		.002
	**2 years**		**47.5 (40.1 to 54.8)**		**23.0 (15.0 to 31.0)**		**N/A**		**N/A**	
		2 years change		7.1 (−1.1 to 15.3)		−13.2 (−22.8 to −3.7)		20.3 (7.8 to 32.9)		.002
**Pulses (g/week)**										
	Baseline		120.7 (112.9 to 128.4)		131.5 (122.5 to 140.4)		N/A		N/A	
	**1 year**		**186.3 (175.6 to 197.1)**		**145.5 (133.8 to 157.2)**		**N/A**		**N/A**	
		1-year change		65.6 (53.9 to 77.4)		14.0 (0.4 to 27.7)		51.6 (33.6 to 69.7)		<.001
	**2 years**		**175.5 (168.4 to 182.6)**		**133.5 (121.5 to 145.4)**		**N/A**		**N/A**	
		2 years change		54.9 (44.6 to 65.1)		2.0 (−12.3 to 16.2)		52.9 (35.3 to 70.4)		<.001
**Nuts (g/week)**										
	Baseline		133.9 (119.7 to 148.0)		88.9 (78.7 to 99.1)		N/A		N/A	
	**1 year**		**200.4 (186.3 to 214.5)**		**103.0 (87.0 to 119.1)**		**N/A**		**N/A**	
		1-year change		66.5 (48.5 to 84.5)		14.2 (−0.23 to 28.6)		52.4 (29.3 to 75.4)		<.001
	**2 years**		**193.8 (177.0 to 210.5)**		**101.8 (87.1 to 116.5)**		**N/A**		**N/A**	
		2 years change		59.9 (40.1 to 79.7)		12.9 (−2.7 to 28.6)		47.0 (21.7 to 72.2)		<.001
**Dairy products (g/week)**										
	Baseline		292.4 (272.5 to 312.3)		333.1 (309.4 to 356.9)		N/A		N/A	
	**1 year**		**301.5 (279.2 to 323.8)**		**351.3 (324.5 to 378.1)**		**N/A**		**N/A**	
		1-year change		9.1 (−11.3 to 29.5)		18.2 (−7.7 to 44.0)		−9.1 (−42.0 to 23.8)		.59
	**2 years**		**308.1 (283.0 to 333.3)**		**379.4 (349.7 to 409.1)**		**N/A**		**N/A**	
		2 years change		15.7 (−7.6 to 39.1)		46.3 (18.8 to 73.8)		−30.5 (−66.6 to 5.5)		.10
**White fish (g/week)**										
	Baseline		443.1 (415.5 to 470.7)		483.4 (453.9 to 513.0)		N/A		N/A	
	**1 year**		**548.7 (521.6 to 575.8)**		**482.1 (448.0 to 516.2)**		**N/A**		**N/A**	
		1-year change		105.6 (74.1 to 137.1)		−1.3 (−32.9 to 30.3)		106.9 (62.3 to 151.6)		<.001
	**2 years**		**528.0 (499.8 to 556.2)**		**450.4 (414.3 to 486.6)**		**N/A**		**N/A**	
		2 years change		84.9 (49.7 to 120.2)		−33.0 (−70.4 to 4.4)		117.9 (66.5 to 169.3)		<.001
**Fatty fish (g/week)**										
	Baseline		210.6 (195.4 to 225.9)		222.0 (204.9 to 239.1)		N/A		N/A	
	**1 year**		**315.3 (290.8 to 339.7)**		**241.1 (222.2 to 260.1)**		**N/A**		**N/A**	
		1-year change		104.6 (79.6 to 129.6)		19.1 (−1.1 to 39.3)		85.5 (53.3 to 117.6)		<.001
	**2 years**		**280.6 (259.0 to 302.2)**		**231.7 (206.4 to 257.0)**		**N/A**		**N/A**	
		2 years change		70.0 (46.1 to 93.8)		9.7 (−13.8 to 33.2)		60.3 (26.8 to 93.7)		<.001
**White meat (g/week)**										
	Baseline		436.6 (414.5 to 458.7)		392.3 (369.7 to 414.8)		N/A		N/A	
	**1 year**		**458.8 (435.3 to 482.3)**		**368.7 (345.6 to 391.7)**		**N/A**		**N/A**	
		1-year change		22.2 (−0.7 to 45.1)		−23.6 (−46.6 to −0.6)		45.8 (13.3 to 78.3)		.006
	**2 years**		**466.0 (440.1 to 491.9)**		**363.0 (331.0 to 395.1)**		**N/A**		**N/A**	
		2 years change		29.4 (2.4 to 56.3)		−29.2 (−62.3 to 3.9)		58.6 (15.9 to 101.2)		.007
**Red and processed meat (g/week)**										
	Baseline		580.6 (542.3 to 619.0)		655.8 (618.1 to 693.5)		N/A		N/A	
	**1 year**		**444.2 (412.0 to 476.4)**		**634.4 (587.9 to 681.0)**		**N/A**		**N/A**	
		1-year change		−136.4 (−174.3 to −98.6)		−21.4 (−63.5 to 20.8)		−115.1 (−171.8 to −58.4)		<.001
	**2 years**		**408.4 (370.9 to 445.9)**		**603.5 (559.8 to 647.2)**		**N/A**		**N/A**	
		2 years change		−172.2 (−213.6 to −130.9)		−52.3 (−90.9 to −13.7)		−119.9 (−176.5 to −63.3)		<.001
**Eggs (g/week)**										
	Baseline		29.3 (27.1 to 31.5)		28.3 (26.3 to 30.3)		N/A		N/A	
	**1 year**		**29.8 (27.6 to 31.9)**		**30.6 (27.8 to 33.3)**		**N/A**		**N/A**	
		1-year change		0.4 (−1.5 to 2.4)		2.2 (−0.3 to 4.8)		−1.8 (−5.0 to 1.4)		.27
	**2 years**		**30.0 (27.7 to 32.3)**		**32.2 (29.0 to 35.3)**		**N/A**		**N/A**	
		2 years change		0.7 (−1.5 to 2.8)		3.9 (1.2 to 6.6)		−3.2 (−6.7 to 0.2)		.07
**Refined olive oil (g/day)**										
	Baseline		2.9 (2.0 to 3.8)		4.0 (2.9 to 5.2)		N/A		N/A	
	**1 year**		**0.3 (0.1 to 0.6)**		**3.9 (2.6 to 5.1)**		**N/A**		**N/A**	
		1-year change		−2.7 (−3.6 to −1.7)		−0.2 (−1.5 to 1.1)		−2.5 (−4.1 to −0.9)		.003
	**2 years**		**0.5 (0.07 to 1.0)**		**5.2 (3.4 to 7.0)**		**N/A**		**N/A**	
		2 years change		−2.4 (−3.3 to −1.5)		1.2 (−0.7 to 3.0)		−3.6 (−5.6 to −1.5)		.001
**Virgin olive oil (g/day)**										
	Baseline		34.5 (32.8 to 36.2)		30.5 (28.7 to 32.3)		N/A		N/A	
	**1 year**		**45.0 (43.5 to 46.5)**		**28.3 (26.3 to 30.3)**		**N/A**		**N/A**	
		1-year change		10.5 (8.5 to 12.4)		−2.2 (−4.4 to −0.0)		12.7 (9.7 to 15.7)		<.001
	**2 years**		**43.3 (41.3 to 45.4)**		**27.9 (25.3 to 30.5)**		**N/A**		**N/A**	
		2 years change		8.8 (6.3 to 11.3)		−2.6 (−5.5 to 0.3)		11.4 (7.6 to 15.3)		<.001
**Other fats than olive oil (g/day)**										
	Baseline		2.0 (1.5 to 2.4)		3.0 (2.2 to 3.7)		N/A		N/A	
	**1 year**		**0.7 (0.4 to 1.0)**		**2.2 (1.6 to 2.7)**		**N/A**		**N/A**	
		1-year change		−1.3 (−1.7 to −0.9)		−0.8 (−1.5 to −0.04)		−0.5 (−1.3 to 0.3)		.25
	**2 years**		**0.7 (0.4 to 1.1)**		**2.9 (1.8 to 4.0)**		**N/A**		**N/A**	
		2 years change		−1.2 (−1.7 to −0.7)		−0.02 (−1.1 to 1.1)		−1.2 (−2.4 to 0.02)		.05
**Sweets (g/week)**										
	Baseline		217.7 (188.7 to 246.6)		392.9 (335.2 to 450.7)		N/A		N/A	
	**1 year**		**174.6 (146.3 to 203.0)**		**443.5 (384.7 to 502.3)**		**N/A**		**N/A**	
		1-year change		−43.0 (−79.3 to −6.7)		50.5 (−25.3 to 126.4)		−93.5 (−177.6 to −9.5)		.03
	**2 years**		**135.8 (115.7 to 155.9)**		**487.8 (419.3 to 556.3)**		**N/A**		**N/A**	
		2 years change		−81.9 (−113.4 to −50.4)		94.9 (17.9 to 171.9)		−176.8 (−260.0 to −93.6)		<.001
**Red wine (g of alcohol/day)**										
	Baseline		5.3 (4.3 to 6.2)		4.4 (3.5 to 5.3)		N/A		N/A	
	**1 year**		**6.3 (5.2 to 7.4)**		**4.9 (3.9 to 6.0)**		**N/A**		**N/A**	
		1-year change		1.0 (0.1 to 1.9)		0.5 (−0.2 to 1.3)		0.5 (−0.7 to 1.7)		.42
	**2 years**		**6.4 (5.1 to 7.6)**		**4.7 (3.7 to 5.8)**		**N/A**		**N/A**	
		2 years change		1.1 (0.04 to 2.2)		0.3 (−0.5 to 1.2)		0.8 (−0.6 to 2.1)		.27
**Other wines than red wine (g of alcohol/day)**										
	Baseline		0.7 (0.2 to 1.1)		0.4 (0.2 to 0.5)		N/A		N/A	
	**1 year**		**0.9 (0.5 to 1.4)**		**0.8 (0.4 to 1.2)**		**N/A**		**N/A**	
		1-year change		0.2 (−0.1 to 0.6)		0.5 (0.1 to 0.9)		−0.2 (−0.7 to 0.2)		.33
	**2 years**		**0.7 (0.1 to 1.2)**		**0.8 (0.4 to 1.2)**		**N/A**		**N/A**	
		2 years change		−0.01 (−0.6 to 0.5)		0.4 (0.07 to 0.8)		−0.4 (−1.1 to 0.2)		.18
**Beer (g of alcohol/day)**										
	Baseline		3.1 (2.5 to 3.8)		2.7 (2.1 to 3.4)		N/A		N/A	
	**1 year**		**2.4 (1.8 to 3.0)**		**3.0 (2.3 to 3.6)**		**N/A**		**N/A**	
		1-year change		−0.7 (−1.3 to −0.07)		0.2 (−0.5 to 1.0)		−0.9 (−1.9 to 0.04)		.06
	**2 years**		**2.5 (1.9 to 3.1)**		**3.3 (2.5 to 4.1)**		**N/A**		**N/A**	
		2 years change		−0.6 (−1.2 to −0.03)		0.5 (−0.4 to 1.5)		−1.2 (−2.3 to −0.06)		.04
**Other alcoholic drinks (g of alcohol/day)**										
	Baseline		0.7 (0.4 to 1.0)		0.5 (0.4 to 0.7)		N/A		N/A	
	**1 year**		**0.6 (0.3 to 0.9)**		**0.8 (0.6 to 1.1)**		**N/A**		**N/A**	
		1-year change		−0.09 (−0.5 to 0.3)		0.3 (0.02 to 0.6)		−0.4 (−0.9 to 0.09)		.11
	**2 years**		**0.4 (0.3 to 0.6)**		**0.9 (0.4 to 1.4)**		**N/A**		**N/A**	
		2 years change		−0.2 (−0.6 to 0.05)		0.4 (−0.09 to 0.8)		−0.6 (−1.2 to −0.07)		.03

^a^Calculated using mixed-effect models with center as random factor.

^b^*P* value between group intervention difference.

^c^N/A: not applicable.

At 2 years of intervention, between-group differences were sustained except for the consumption of vegetables, beer, and other alcoholic drinks (liquors and distilled beverages). There was a significant increase in the consumption of vegetables within the intervention group, although the difference in changes between the intervention groups was not statistically significant. The intervention group reduced the consumption of beer and other alcoholic drinks (liquors and distilled beverages) compared with the control group after 2 years of follow-up, but not during the first year of follow-up.

### Energy and Nutrient Intake

Consistent with changes in consumption of food groups associated with the Mediterranean diet, significant between-group differences were observed for increased intake of fat, MUFA, PUFA, omega-3, and fiber, for the intervention group versus the control group at 1 year of the intervention (Table 3). After a 2-year follow-up, there were no significant differences between groups in the intake of fat, MUFA, and PUFA. The intervention group showed a decrease in the intake of sodium at 1 and 2 years of follow-up and SFA and carbohydrates after 2 years when compared with the control group.

**Table 3:** Baseline nutrient intake and changes by randomized treatment group at 12- and 24-month follow-up visits of participants in the prevention of recurrent arrythmias with Mediterranean diet trial.

Energy or nutrient			Group intervention				Between group difference^a^, mean (95% CI)		*P* value^b^	
			Intervention, mean (95% CI)		Control, mean (95% CI)					
**Energy (kcal/day)**										
	Baseline		2396 (2328 to 2465)		2527 (2443 to 2612)		N/A^c^		N/A	
	**1 year**		**2377 (2320 to 2435)**		**2466 (2379 to 2552)**		**N/A**		**N/A**	
		1-year change		−9.1 (−85.9 to 47.7)		−61.6 (−154.8 to 31.7)		42.5 (−71.2 to 157.2)		.47
	**2 years**		**2250 (2191 to 2309)**		**2496 (2388 to 2604)**		**N/A**		**N/A**	
		2 years change		−1467 (−224 to -68.1)		−31.5 (−133.5 to 70.4)		−114.6 (−243.0 to 13.7)		.08
**Fat (g/day)**										
	Baseline		104.8 (101.8 to 107.8)		107.6 (103.7 to 111.5)		N/A		N/A	
	**1 year**		**110.4 (107.6 to 113.3)**		**106.0 (102.0 to 109.9)**		**N/A**		**N/A**	
		1-year change		5.6 (2.2 to 9.1)		−1.6 (−6.0 to 2.7)		7.3 (1.7 to 12.8)		.01
	**2 years**		**106.0 (102.9 to 109.0)**		**110.0 (105.0 to 115.0)**		**N/A**		**N/A**	
		2 years change		1.2 (−2.6 to 4.9)		2.4 (−2.6 to 7.5)		−1.3 (−7.6 to 5.1)		.70
**MUFA^d^ (g/day)**										
	Baseline		54.2 (52.6 to 55.9)		54.6 (52.5 to 56.8)		N/A		N/A	
	**1 year**		**59.9 (58.2 to 61.7)**		**54.5 (52.2 to 56.9)**		**N/A**		**N/A**	
		1-year change		5.7 (3.5 to 7.8)		−0.1 (−2.7 to 2.5)		5.8 (2.5 to 9.1)		.001
	**2 years**		**57.3 (55.3 to 59.3)**		**57.0 (54.1 to 59.9)**		**N/A**		**N/A**	
		2 years change		3.0 (0.7 to 5.4)		2.3 (−0.8 to 5.4)		0.7 (−3.2 to 4.6)		.72
**PUFA^e^ (g/day)**										
	Baseline		18.7 (18.0 to 19.3)		18.7 (17.9 to 19.5)		N/A		N/A	
	**1 year**		**20.5 (19.7 to 21.2)**		**18.3 (17.5 to 19.2)**		**N/A**		**N/A**	
		1-year change		1.8 (0.9 to 2.7)		−0.4 (−1.3 to 0.6)		2.2 (0.9 to 3.5)		.001
	**2 years**		**19.7 (18.8 to 20.5)**		**18.6 (17.7 to 19.6)**		**N/A**		**N/A**	
		2 years change		1.0 (0.02 to 2.0)		−0.06 (−1.1 to 1.0)		1.1 (−0.4 to 2.5)		.15
**SFA^f^ (g/day)**										
	Baseline		26.2 (25.3 to 27.2)		27.8 (26.6 to 28.9)		N/A		N/A	
	**1 year**		**24.6 (23.8 to 25.3)**		**27.2 (26.0 to 28.3)**		**N/A**		**N/A**	
		1-year change		−1.7 (−2.5 to −0.8)		−0.6 (−1.7 to 0.5)		−1.1 (−2.5 to 0.3)		.14
	**2 years**		**23.7 (22.9 to 24.6)**		**28.2 (26.8 to 29.7)**		**N/A**		**N/A**	
		2 years change		−2.5 (−3.5 to −1.5)		0.5 (−0.8 to 1.7)		−3.0 (−4.6 to −1.3)		<.001
**Omega 3 (mg/day)**										
	Baseline		0.8 (0.8 to 0.8)		0.9 (0.8 to 0.9)		N/A		N/A	
	**1 year**		**1.1 (1.0 to 1.2)**		**0.9 (0.9 to 1.0)**		**N/A**		**N/A**	
		1-year change		0.3 (0.2 to 0.4)		0.05 (−0.01 to 0.1)		0.3 (0.2 to 0.4)		<.001
	**2 years**		**1.0 (0.9 to 1.1)**		**0.9 (0.8 to 1.0)**		**N/A**		**N/A**	
		2 years change		0.2 (0.1 to 0.3)		0.01 (−0.05 to 0.08)		0.2 (0.09 to 0.3)		<.001
**Protein (g/day)**										
	Baseline		94.3 (91.8 to 96.8)		99.9 (97.2 to 102.5)		N/A		N/A	
	**1 year**		**96.3 (93.9 to 98.7)**		**98.8 (95.8 to 101.8)**		**N/A**		**N/A**	
		1-year change		2.0 (−0.4 to 4.4)		−1.1 (−3.9 to 1.8)		3.1 (−0.6 to 6.8)		.10
	**2 years**		**91.3 (88.8 to 93.8)**		**97.7 (94.0 to 101.4)**		**N/A**		**N/A**	
		2 years change		−3.0 (−5.6 to −0.3)		-2.2 (−5.4 to 1.1)		−0.8 (−5.0 to 3.4)		.70
**Carbohydrates (g/day)**										
	Baseline		251.5 (241.4 to 261.6)		275.7 (264.1 to 287.2)		N/A		N/A	
	**1 year**		**231.3 (223.5 to 239.1)**		**262.0 (250.5 to 273.5)**		**N/A**		**N/A**	
		1-year change		−20.2 (−29.9 to −10.5)		−13.7 (−26.6 to −0.7)		−6.6 (−22.7 to 9.6)		.43
	**2 years**		**215.1 (206.8 to 223.5)**		**260.3 (246.1 to 274.5)**		**N/A**		**N/A**	
		2 years change		−36.4 (−47.7 to −25.1)		−15.3 (−29.4 to −1.3)		−21.0 (−39.1 to −3.0)		.02
**Fiber (g/day)**										
	Baseline		24.1 (23.2 to 25.0)		25.1 (24.1 to 26.2)		N/A		N/A	
	**1 year**		**30.2 (29.2 to 31.2)**		**25.7 (24.5 to 26.9)**		**N/A**		**N/A**	
		1-year change		6.1 (5.0 to 7.2)		0.5 (−0.8 to 1.9)		5.6 (3.8 to 7.3)		<.001
	**2 years**		**28.2 (27.1 to 29.3)**		**25.1 (23.8 to 26.5)**		**N/A**		**N/A**	
		2 years change		4.1 (2.9 to 5.2)		−0.02 (−1.5 to 1.4)		4.1 (2.2 to 6.0)		<.001
**Sodium (mg/day)**										
	Baseline		3390 (3267 to 3512)		3523 (3398 to 3648)		N/A		N/A	
	**1 year**		**2831 (2719 to 2943)**		**3254 (3119 to 3390)**		**N/A**		**N/A**	
		1-year change		−558.4 (−684.3 to −432.6)		−268.9 (−403.9 to −133.9)		−289.5 (−474.1 to −105.0)		.002
	**2 years**		**2586 (2464 to 2709)**		**3214 (3048 to 3380)**		**N/A**		**N/A**	
		2 years change		−803.6 (-943.1 to −664.1)		−308.9 (−472.0 to −145.7)		−494.7 (−709.4 to −280.1)		<.001

^a^Calculated using mixed-effect models with center as random factor.

^b^*P* value between group intervention difference.

^c^N/A: not applicable.

^d^MUFA: monounsaturated fatty acid.

^e^PUFA: polyunsaturated fatty acid.

^f^SFA: saturated fatty acid.

### Hydroxytyrosol Intake

Finally, regarding total hydroxytyrosol intake, no significant differences between groups at the 1- and 2-year follow-up visits were found. However, when we studied the intake of hydroxytyrosol derived from olive oil (one of the two main sources of this polyphenol in the diet), we observed that the intervention group increased the intake of hydroxytyrosol from olive oil after 1 and 2 years of follow-up, compared with the control group. Meanwhile, the intake of hydroxytyrosol derived from wine (the other main source of hydroxytyrosol of the diet) did not change at 1- and 2-year follow-up visits between groups.

## Discussion

### Principal Findings

First, this work describes in detail the nutritional intervention of the PREDIMAR trial, which is, to our knowledge, the first remote dietary intervention based on the Mediterranean diet specifically designed for patients with AF treated with catheter ablation. Second, our results demonstrate that a remote nutritional intervention is a useful tool kit to improve the quality of the diet according to the goals of the Mediterranean diet.

### Comparison With Prior Work

Although nutritional interventions are typically derived face to face, at present, remote nutritional interventions (web page, mobile phone app, email, text messaging, and phone calls) are becoming more frequent. This could be in part because of the increase in the use of the internet and other technological resources as well as the high economic costs of traditional interventions. As far as we know, our research is unique in that it is a remote nutritional intervention using different behavioral change strategies including a web page and mobile app, printed material, and personalized advice by phone call and email. Remotely, intervention permits overcoming the barriers of in-person interventions such as lack of staff and institutional resources to reach a large number of participants, and that the participants have to attend to the onsite meetings (group or individual meetings), which in turn require substantial organizational skills [36]. On the other hand, the provision of personalized advice is associated with a greater change in dietary habits [36]. However, Hutchesson et al [37] reported that a web page–based intervention combined with comprehensive personalized feedback reports conducted similar improvements in dietary intake compared with the web-page-based intervention group. Therefore, we hypothesized that combining different nutritional intervention remotely tools could improve in a better manner the impact of the intervention. In fact, a previous systematic review concluded that using multiple modes of communication increases the effectiveness of remote intervention [22]. Moreover, it is widely acknowledged that web-based interventions provide effective changes in physical activity, eating behaviors, and weight loss [21-23]. To maintain engagement with the nutritional intervention, biweekly automated email notifications were sent to each participant. Moreover, we also used printed material to overcome potential barriers to internet access, especially among older participants [38,39]. In this context, the ManUp study concluded that an information technology-based (web and mobile) intervention was as effective as a print-based intervention [38].

Participants’ baseline scores showed that they had a reasonably good Mediterranean-style food pattern, according to previous studies developed in the Mediterranean area with patients at risk or with any cardiovascular disease [40-43]. The difference between groups in the MEDAS score at baseline is consistent with previous nutritional studies [40,41,43] and could be due in part to social desirability bias, which means that participants in the intervention group tended to report higher Mediterranean diet adherence. After 2 years of follow-up, not only the intervention group, but also the control group increased the adherence to the Mediterranean diet. In this sense, it is possible that participants in the control group were aware of the relationship between food habits and cardiovascular health and thus were motivated to change their dietary habits after catheter ablation. However, the improvement in the adherence to the Mediterranean diet was significantly higher in the intervention group, and the magnitude of the observed changes was similar to that in face-to-face intervention studies. As an example, in the PREDIMED trial, participants randomized to the Mediterranean diet group showed an increase of 2.3 points in the MEDAS score after the 12-month intervention period [40]. A more recent example is the PREDIMED-plus trial, in which the mean increase in adherence to the Mediterranean diet was 2.1 points in the Mediterranean diet group (control group) after 1 year of follow-up [42]. Moreover, our results are consistent with those of previous online intervention studies. Recio-Rodriguez et al [44] and Choi et al [45] demonstrated, using the MEDAS questionnaire, the efficacy of a mobile application intervention in promoting the Mediterranean diet. Neither study found a significant difference with the Mediterranean diet changes found in the face-to-face intervention group.

We observed a higher increase in the consumption of plant-based foods (fruits, vegetables, whole grain cereals, olive oil, pulses, and nuts) and fish in the intervention group than in the control group during the intervention period. Similar results were found in the PREDIMED study after 1 year of face-to-face intervention [40,41]. Moreover, previous online intervention studies found an increase in the intake of fruit and vegetables [22,46]. Other authors have reported a significant increase in the consumption of total fat and different fatty acids after different online interventions [47,48]. Finally, Hutchesson et al [37] demonstrated that participants of a web-based weight loss program significantly reduced energy-dense, nutrient-poor foods.

It is evident that the intervention group adopted healthier behaviors during the first year of follow-up. However, in general, dietary habits were sustained in the longer term, and even a high decrease in the intake of red and processed meat, refined cereals, and sweets was observed at 2 years of follow-up among participants of the intervention group. These findings are consistent with those reported in the literature [49,50].

In line with previous intervention studies, we found an increase of 1 serving per day of VOO (including extra virgin) and a reduction of refined olive oil in the intervention group compared with the control group [42]. The magnitude of the change in the consumption of VOO may reflect a small increase in the individual effect, but a mean change of this magnitude may have a great impact at the population level. In this sense, a recent study demonstrated that, compared with nonconsumers or low consumers (0-<4.5 g per day), those with a high consumption of olive oil (>7 g per day) had 14% and 18% lower risk of cardiovascular and coronary heart disease, respectively [51]. On the other hand, previous research suggests that free provision of key food items is an effective strategy to increase adherence to the prescribed interventions [40,42]. Therefore, we hypothesized that the remote intervention combining general nutritional information and personalized advice, together with the free provision of EVOO, may be very effective in increasing adherence to a Mediterranean dietary pattern.

### Limitations

There are several strengths and limitations of this study that should be considered when interpreting the results. First, these findings are based on preliminary analyses within the context of an ongoing randomized controlled trial, and it is unknown whether and how these results may be related to beneficial health outcomes. Second, the results of the nutritional intervention may not be applicable to the general population for 2 main reasons. On the one hand, the population of this study was patients with AF, and therefore they could be worried about their health. On the other hand, the free provision of EVOO, which could be a strength of our study, can also represent a barrier because of the high cost of this product. Third, although the clinical providers were blinded to the allocation group, the dietitians were not blinded. Fourth, we used a self-reported FFQ instead of objective instruments, such as biomarkers. Recall bias, social desirability bias, and other potential reporting biases may have affected the results. However, the FFQ has been previously validated and is suitable for repeatedly ranking people according to their food and nutrient intake [30]. Fifth, the FFQ did not differ between the consumption of VOO and EVOO. However, in Spain, the consumption of EVOO outweighs the consumption of VOO (39% vs 9%) [52] and both have similar contents of MUFA and polyphenols [34,53,54]. A higher content of MUFA and polyphenols explains the attributed health effects of olive oil; therefore, both VOO and EVOO could have a similar beneficial effect on AF. Sixth, self-reported use of nutritional intervention tools (website, app, and printed materials) may not fully reflect the completion of health education. However, periodical phone calls from the dietitian were used as a monitor system to assess and meet the educational needs of each participant in the intervention group. Finally, we acknowledge that our results do not provide evidence to indicate that a remote intervention is more effective than an in-person intervention because this study did not use a control group with face-to-face intervention.

### Conclusions

We found that a multifaceted remote nutritional intervention seems to be effective in increasing the knowledge and skills of participants and improving their dietary intake in the direction of the Mediterranean diet pattern. Moreover, our study suggests that remote health promotion interventions could offer a cost-effective community approach to address the increasing health burden.

## Appendix

Multimedia Appendix 1 Clinical follow-up visits and intervention period.

Multimedia Appendix 2 English version of the 14-item Mediterranean adherence screener (MEDAS) questionnaire.

Multimedia Appendix 3 Screenshots of the prevention of recurrent arrythmias with Mediterranean diet (PREvención con DIeta Mediterránea de Arritmias Recurrentes) website.

Multimedia Appendix 4 Screenshots of the prevention of recurrent arrythmias with Mediterranean diet app.

Multimedia Appendix 5 Food frequency questionnaire.

Multimedia Appendix 6 Adherence to the Mediterranean diet at baseline, after 12- and 24-month follow-up visits according to intervention groups.

Multimedia Appendix 7 CONSORT-eHEALTH (V 1.6.1).

## Abbreviations

AF: atrial fibrillation
EVOO: extra virgin olive oil
FFQ: food frequency questionnaire
MEDAS: Mediterranean diet adherence screener
MUFAs: monounsaturated fatty acids
PREDIMAR: prevention of recurrent arrythmias with Mediterranean diet (PREvención con DIeta Mediterránea de Arritmias Recurrentes)
PREDIMED: prevention with Mediterranean diet (PREvención con DIeta MEDiterránea)
PUFAs: polyunsaturated fatty acids
SFA: saturated fatty acids
VOO: virgin olive oil
