# Supplementary material for: A Remote Nutritional Intervention to Change the Dietary Habits of Patients Undergoing Ablation of Atrial Fibrillation: Randomized Controlled Trial
Source: J Med Internet Res. 2020 Dec 7;22(12):e21436. doi: 10.2196/21436 (PMC7752535; doi:10.2196/21436)
Supplement: Multimedia Appendix 1 [file jmir_v22i12e21436_app1.pdf]

Multimedia Appendix 1. Clinical follow-up visits and intervention period

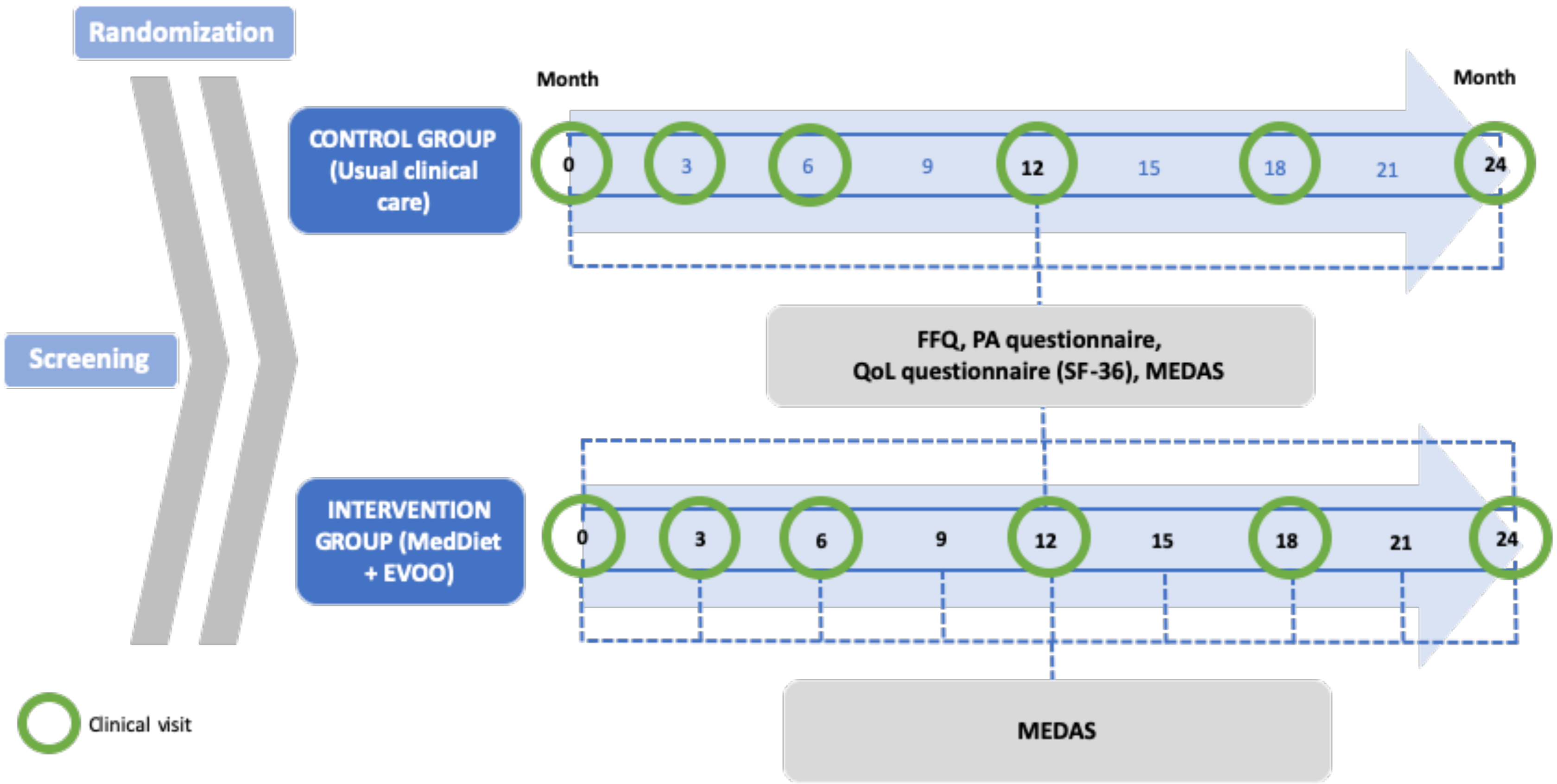

MedDiet, Mediterranean Diet; EVOO, Extra virgin olive oil; FFQ, Food frequency questionnaire; PA, Physical activity; QoL, Quality of life; MEDAS, Mediterranean Diet Adherence Screener
