# Supplementary material for: A Remote Nutritional Intervention to Change the Dietary Habits of Patients Undergoing Ablation of Atrial Fibrillation: Randomized Controlled Trial
Source: J Med Internet Res. 2020 Dec 7;22(12):e21436. doi: 10.2196/21436 (PMC7752535; doi:10.2196/21436)
Supplement: Multimedia Appendix 2 [file jmir_v22i12e21436_app2.pdf]

MULTIMEDIA APPENDIX 2. ENGLISH VERSION OF THE 14-ITEM MEDITERRANEAN ADHERENCE SCREENER (MEDAS) QUESTIONNAIRE

	Yes (1 point)	No (0 points)
1. Do you use olive oil as the principal source of fat for cooking?		
2. How much olive oil do you consume per day (including that used in frying, meals eaten away from home, salads, etc.)?¿4 or more tablespoons? *(1 tablespoon=13.5g)*		
3. How many servings of vegetables do you consume per day? ¿2 or more? (at least 1 portion raw or salad) *(1 serving = 200g – side dishes count as ½ a serving, not including potatoes or sweetcorn)*		
4. How many pieces of fruit (including fresh-squeezed fruit juice) do you consume per day? ¿3 or more? *(not including frozen or dried fruit)*		
5. How many servings of red meat, hamburger, or meat products (ham, sausage, etc.) do you consume per day? ¿less than 1? *(1 serving = 100-150g)*		
6. How many servings of butter, margarine, or cream do you consume per day? ¿less than 1? *(1 serving = 12g, 1 tablespoon)*		
7. How many sugar-sweetened beverages do you drink per day? ¿less than 1? (1 cup = 100ml)		
8. How much wine do you drink per week? ¿7 or more glasses? (1 glass = 125ml)		
9. How many servings of pulses do you consume per week? ¿3 or more? *(1 serving = 150g) (including canned varieties)*		
10. How many servings of fish or shellfish/seafood do you consume per week? ¿3 or more? *(1 serving = 100-150g fish, or 4-5 pieces or 200g shellfish)*		
11. How many times per week do you consume commercial sweets or pastries (not homemade), such as cakes, cookies, biscuits, or custard? ¿less than 2?		
12. How many servings of nuts (including peanuts) do you consume per week? ¿3 or more? *(1 serving = 30g)*		
13. Do you prefer to eat chicken, turkey, or rabbit meat instead of beef, pork, hamburgers, or sausages?		
14. How many times per week do you consume cooked vegetables, pasta, rice, or other dishes prepared with a sauce of tomato, garlic, onions or leeks sautéed in olive oil (sofrito)? ¿2 or more?		

Papadaki A, Johnson L, Toumpakari Z, England C, Rai M, Toms S, Penfold C, Zazpe I, Martínez-González MA, Feder G. Nutrients 2018;(10):138. [doi: 10.3390/nu10020138]
