# Supplementary material for: A Remote Nutritional Intervention to Change the Dietary Habits of Patients Undergoing Ablation of Atrial Fibrillation: Randomized Controlled Trial
Source: J Med Internet Res. 2020 Dec 7;22(12):e21436. doi: 10.2196/21436 (PMC7752535; doi:10.2196/21436)

Multimedia Appendix 3

Screenshots of the Predimar website

www.predimar.es

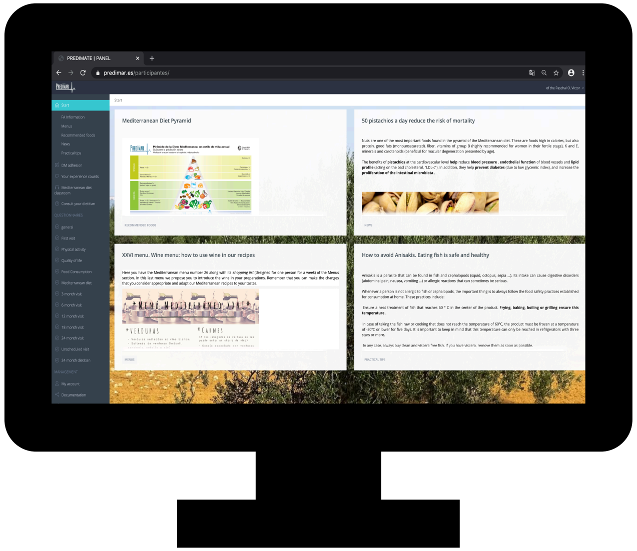

FOREGROUND: PREDIMAR WEB

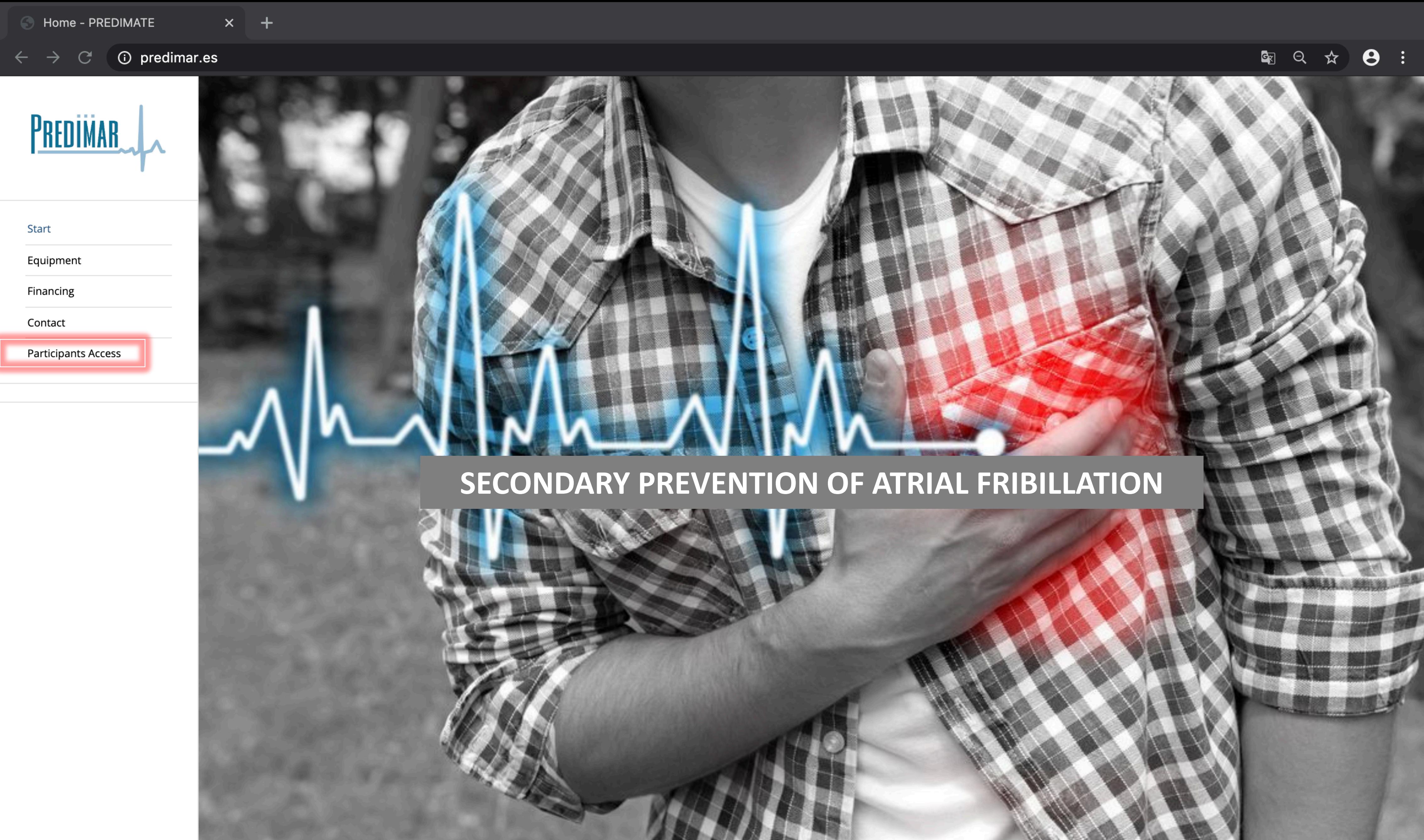

LOG IN

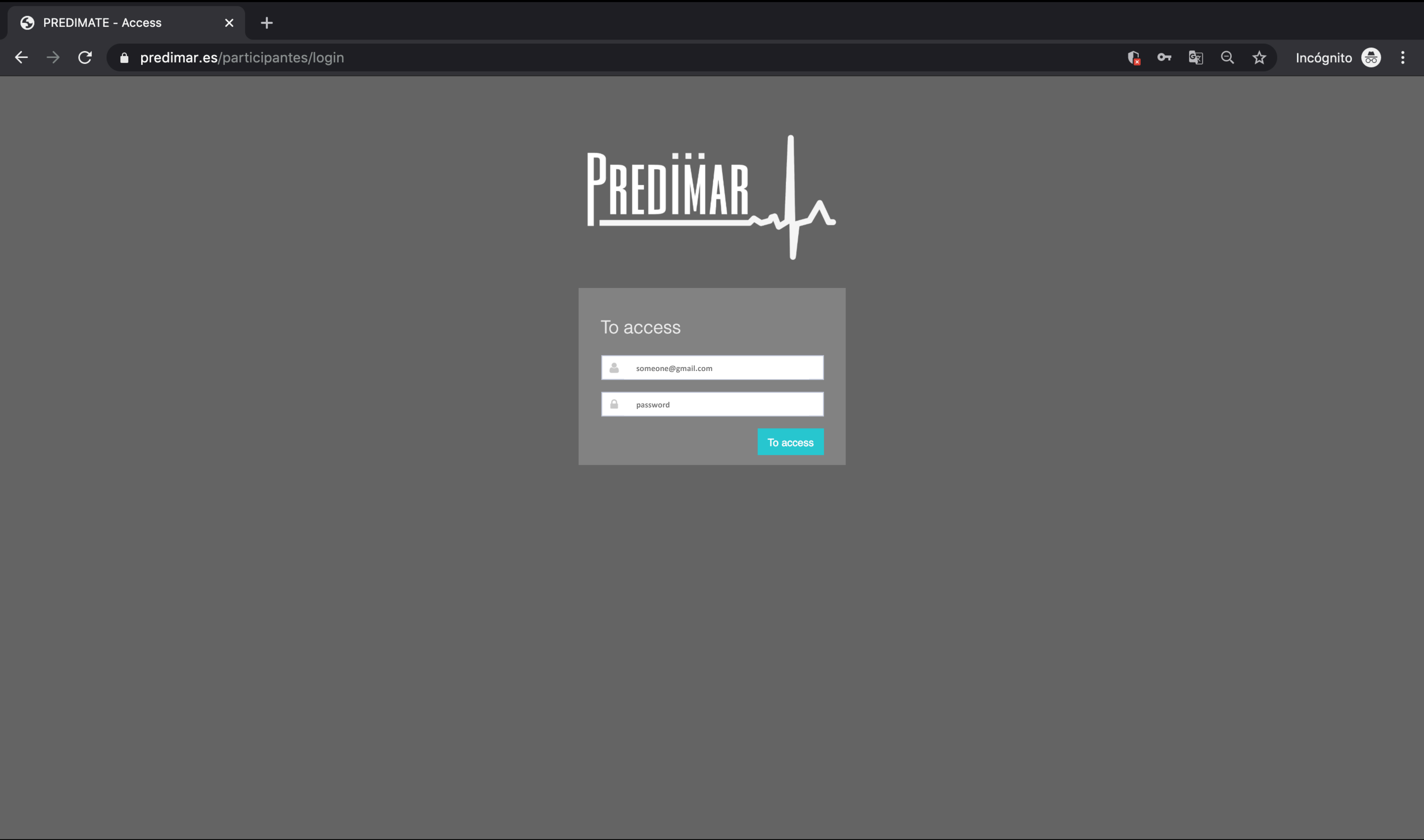

PREDIMATE - Access
predimar.es/participantes/login
Incógnito
PREDIMAR
To access
someone@gmail.com
password
To access

BACKGROUND OF HOME SCREEN

PREDIMATE | PANEL

predimar.es/participantes/

of the Paschal O, Victor

- Start
- FA Information
- Menus
- Recommended foods
- News
- Practical tips
- DM adhesion
- Your experience counts
- Mediterranean diet classroom
- Consult your dietitian

QUESTIONNAIRES

- general
- First visit
- Physical activity
- Quality of life
- Food Consumption
- Mediterranean diet
- 3 month visit
- 6 month visit
- 12 month visit
- 18 month visit
- 24 month visit
- Unscheduled visit
- 24 month dietitian

Start

Mediterranean Diet Pyramid

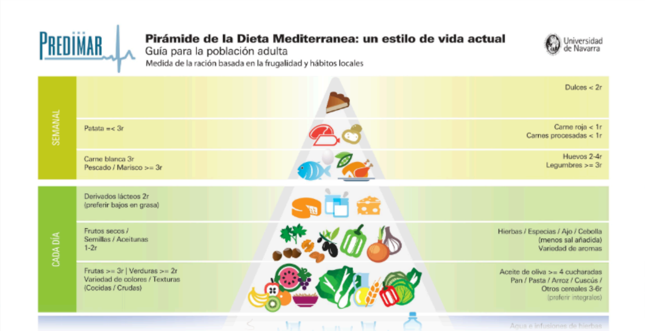

RECOMMENDED FOODS

50 pistachios a day reduce the risk of mortality

Nuts are one of the most important foods found in the pyramid of the Mediterranean diet. These are foods high in calories, but also protein, good fats (monounsaturated), fiber, vitamins of group B (highly recommended for women in their fertile stage), K and E, minerals and carotenoids (beneficial for macular degeneration presented by age).

The benefits of **pistachios** at the cardiovascular level **help** reduce **blood pressure** , **endothelial function** of blood vessels and **lipid profile** (acting on the bad cholesterol, "LDL-c"). In addition, they help **prevent diabetes** (due to low glycemic index), and increase the **proliferation of the intestinal microbiota** .

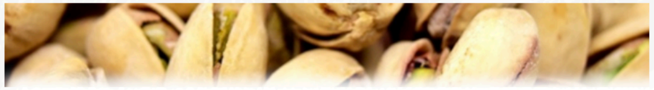

NEWS

XXVI menu. Wine menu: how to use wine in our recipes

Here you have the Mediterranean menu number 26 along with its *shopping list* (designed for one person for a week) of the Menus section. In this last menu we propose you to introduce the wine in your preparations. Remember that you can make the changes that you consider appropriate and adapt our Mediterranean recipes to your tastes.

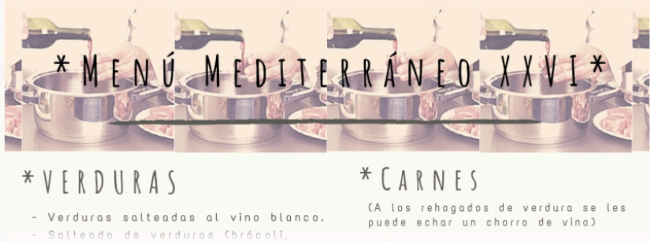

MENUS

How to avoid Anisakis. Eating fish is safe and healthy

Anisakis is a parasite that can be found in fish and cephalopods (squid, octopus, sepia ...). Its intake can cause digestive disorders (abdominal pain, nausea, vomiting ...) or allergic reactions that can sometimes be serious.

Whenever a person is not allergic to fish or cephalopods, the important thing is to always follow the food safety practices established for consumption at home. These practices include:

Ensure a heat treatment of fish that reaches 60 ° C in the center of the product. **Frying, baking, boiling or grilling ensure this temperature** .

In case of taking the fish raw or cooking that does not reach the temperature of 60ºC, the product must be frozen at a temperature of -20ºC or lower for five days. It is important to keep in mind that this temperature can only be reached in refrigerators with three stars or more.

In any case, always buy clean and viscera free fish. If you have viscera, remove them as soon as possible.

PRACTICAL TIPS

MENU OPTIONS

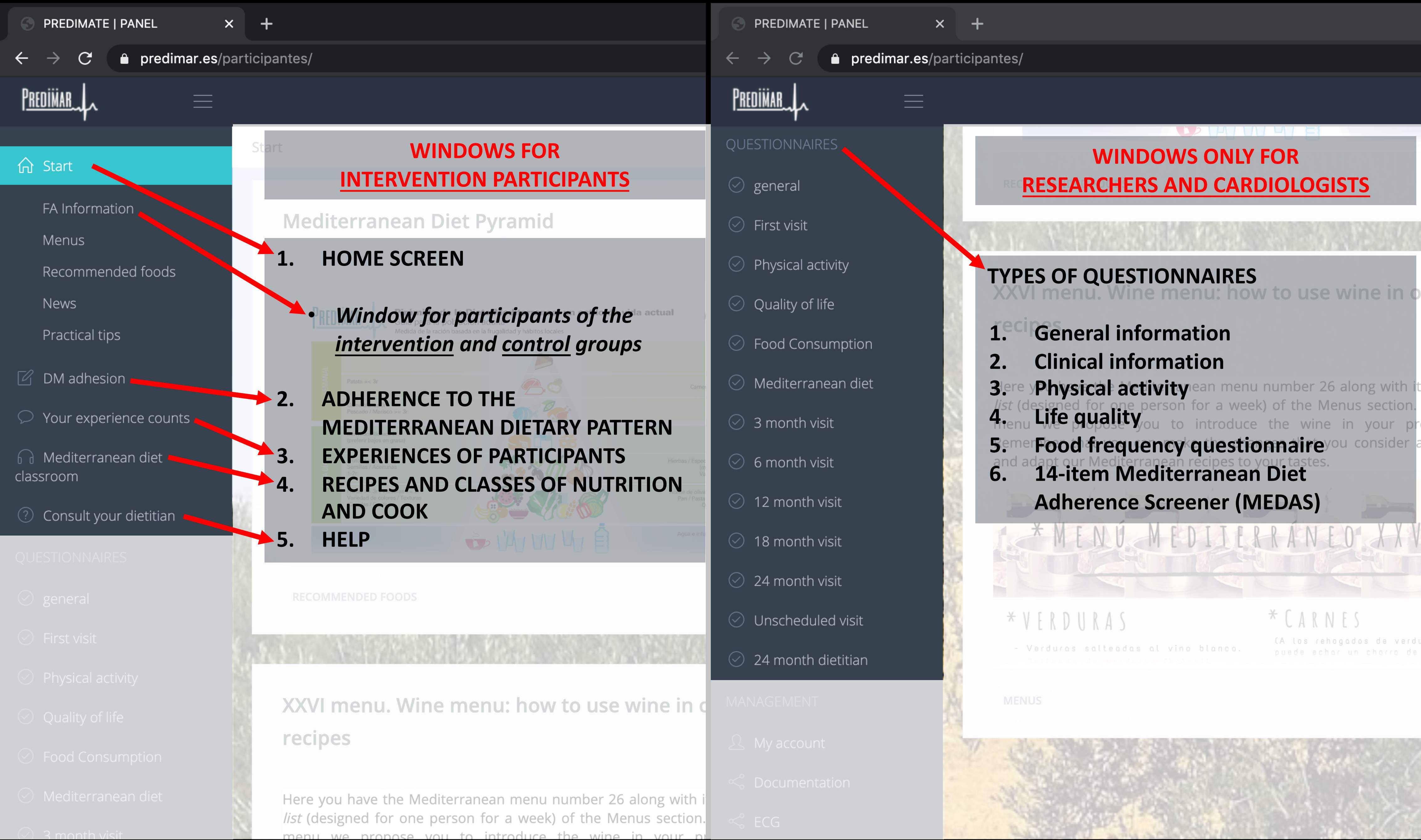

1. BACKGROUND OF HOME SCREEN: ATRIAL FIBRILLATION INFORMATION

PREDIMATE | PANEL

predimar.es/participantes/tag/informacion

PREDIMAR

of the Paschal O, Victor

- Practical tips
- DM adhesion
- Your experience counts
- Mediterranean diet classroom
- Consult your dietitian

QUESTIONNAIRES

- general
- First visit
- Physical activity
- Quality of life
- Food Consumption
- Mediterranean diet
- 3 month visit
- 6 month visit
- 12 month visit
- 18 month visit
- 24 month visit
- Unscheduled visit
- 24 month dietitian

MANAGEMENT

- My account
- Documentation
- ECG
- Variables
- Users
- Participants
- Registrars

General Ablation Recommendations

The first two days after the procedure we recommend **relative rest at home.** It does not mean bed rest, if not **avoid walking too much.** This simple measure prevents bruising [illegible] can progressively make normal life.

To **restart the sports activity we** recommend **waiting two to three weeks.**

During the first three months we recommend **avoiding risky or contact sports as much** as **possible** since it is very likely that you are taking anticoagulant treatment.

After the ablation you can **travel with total normality** in any means of transport.

If you have **arrhythmia during the first weeks after the ablation, you should not worry because it is normal and very frequent.** Our recommendation is that **if it lasts** [illegible] **the emergency room** to assess what it is and to be put.

What is ablation?

How is catheter ablation of the pulmonary veins done?

-The electrical origin in the heart of atrial fibrillation is in the pulmonary veins. The pulmonary veins are 4 veins that carry blood from the [illegible] left atrium.

-The walls of these veins emit electrical impulses at a very fast and disorganized frequency and transmit them to the heart. Ablation treat[illegible]cal impulses from passing from these veins to the heart. For this we make a physical barrier between the veins and the left atrium. These barriers [illegible]on) or radiofrequency.

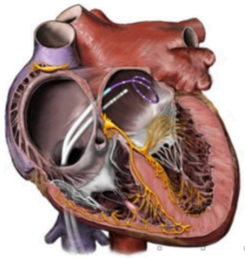

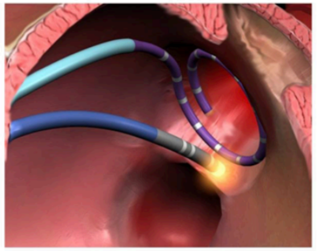

Normal symptoms after ablation:

1.- The first few days after the ablation it is normal that you are somewhat tired. This state usually lasts a week, after which each day you will feel better.

2.- You may have chest pain. It is very common that this pain increases with breathing or postural changes. This pain can be calmed with PARACETAMOL. If you do not improve, you should consult your cardiologist.

3.- You may have a sore throat in the days after the ablation. It is due to orotracheal intubation and it is normal to decrease after 2-3 days.

4.- The first 3 MONTHS after the ablation it is NORMAL to have arrhythmia but it does not mean that the ablation has gone wrong. After ablation, the heart undergoes a very intense inflammatory response and episodes of atrial fibrillation are normal. It is even more frequent the days immediately after the procedure.

PREDIMATE | PANEL

predimar.es/p

PREDIMAR

- Start
- FA Information
- Menus
- Recommended foods
- News
- Practical tips
- DM adhesion
- Your experience counts
- Mediterranean diet classroom
- Consult your dietitian

1. BACKGROUND OF HOME SCREEN: MONTHLY MENUS

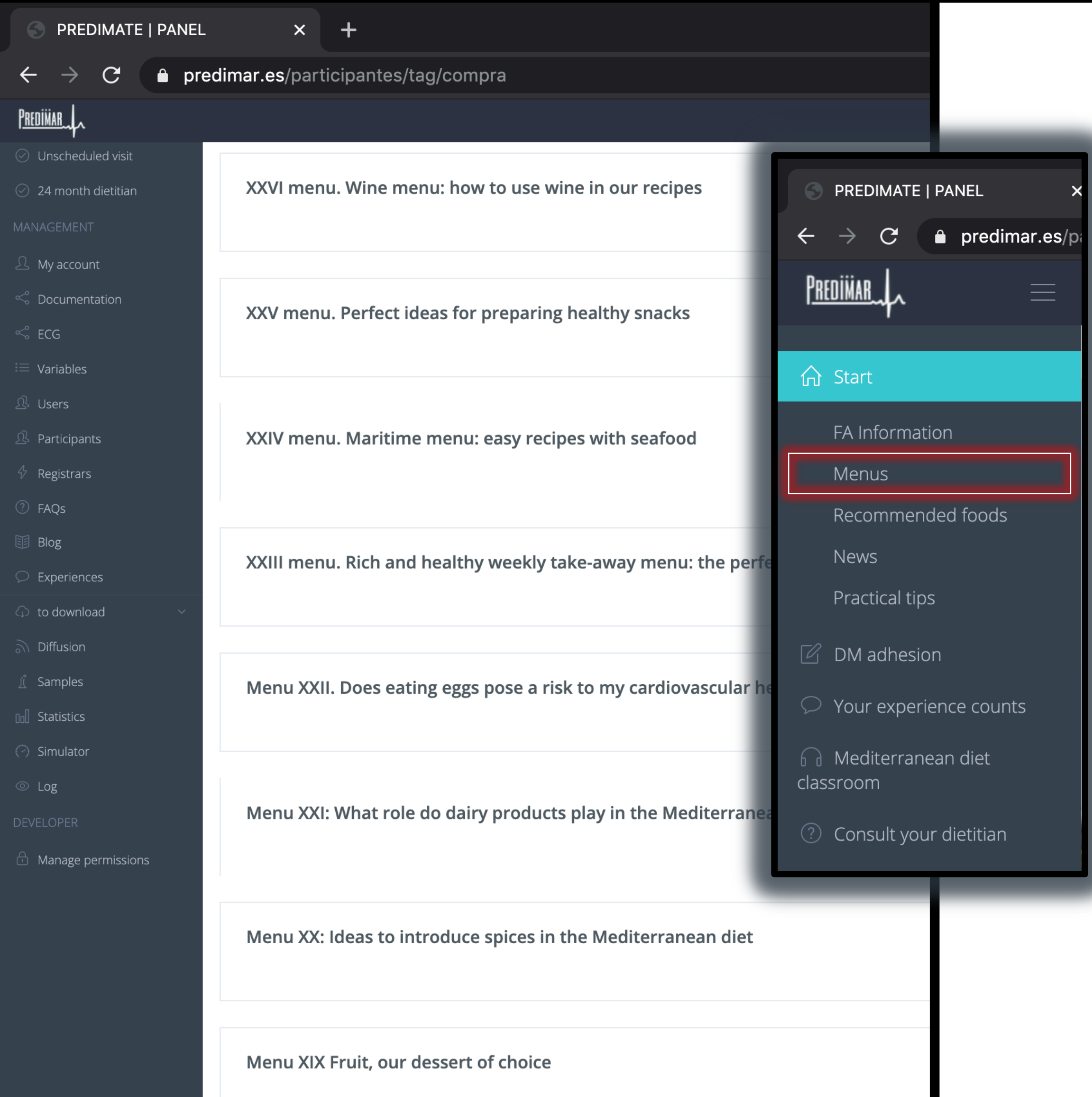

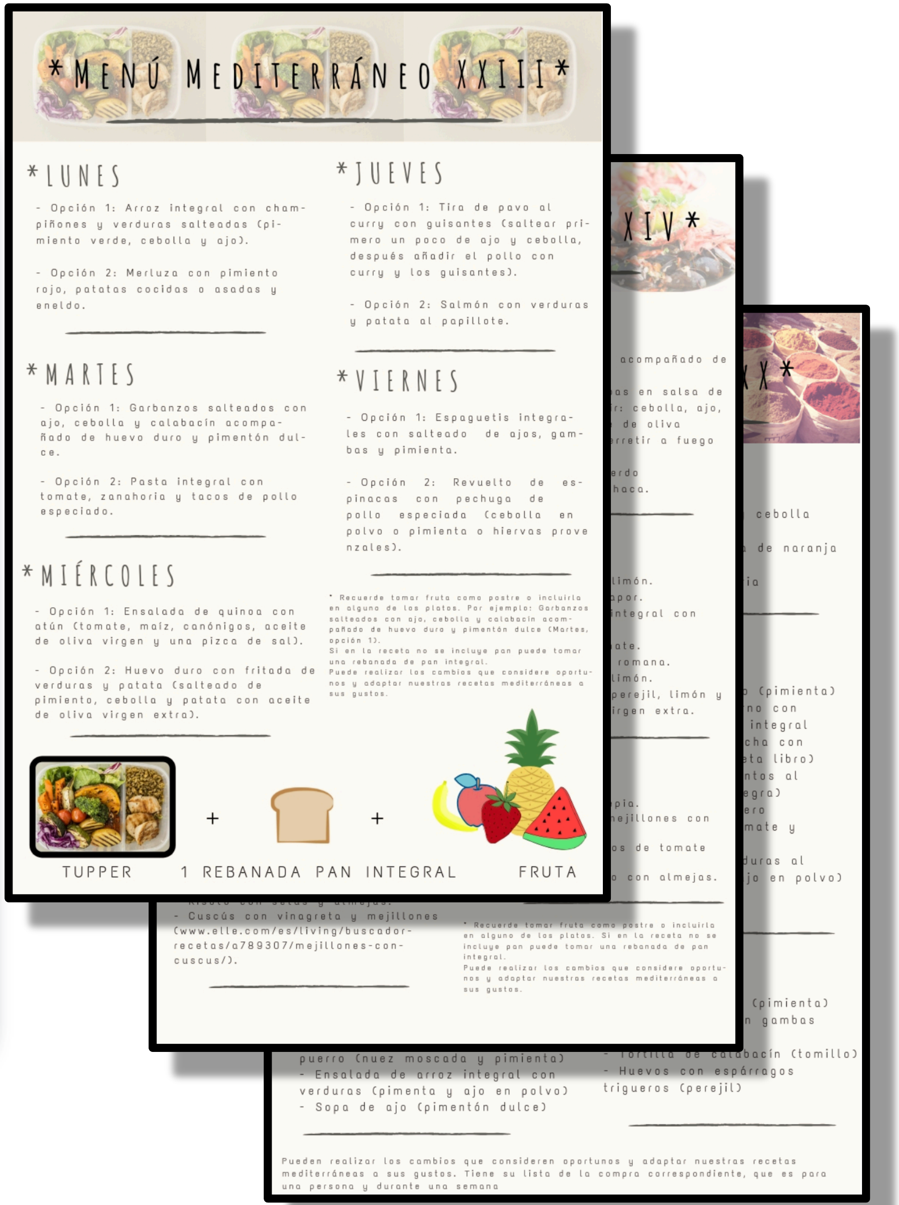

1. BACKGROUND OF HOME SCREEN: RECOMMENDED FOODS

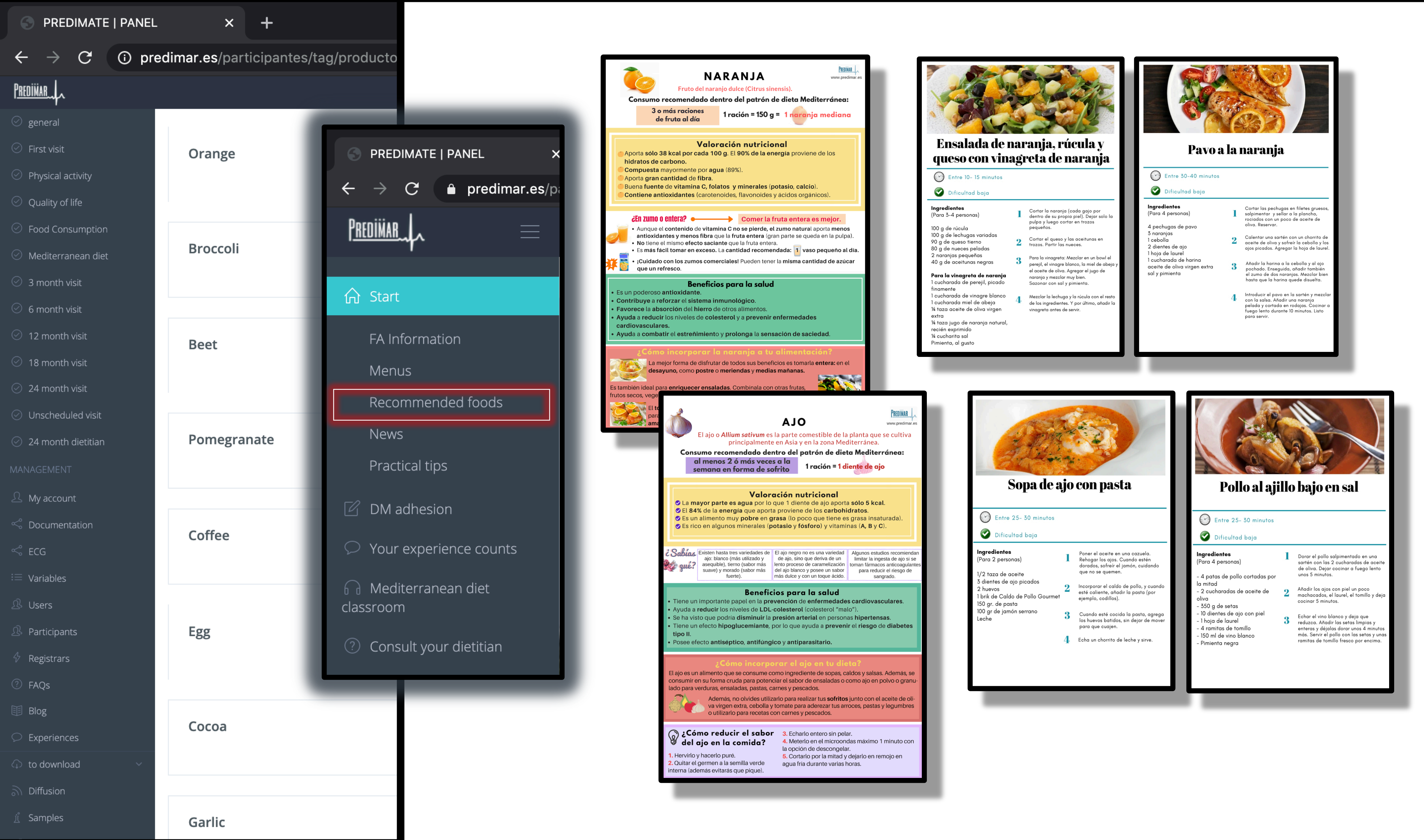

1. BACKGROUND OF HOME SCREEN: RECOMMENDED FOODS

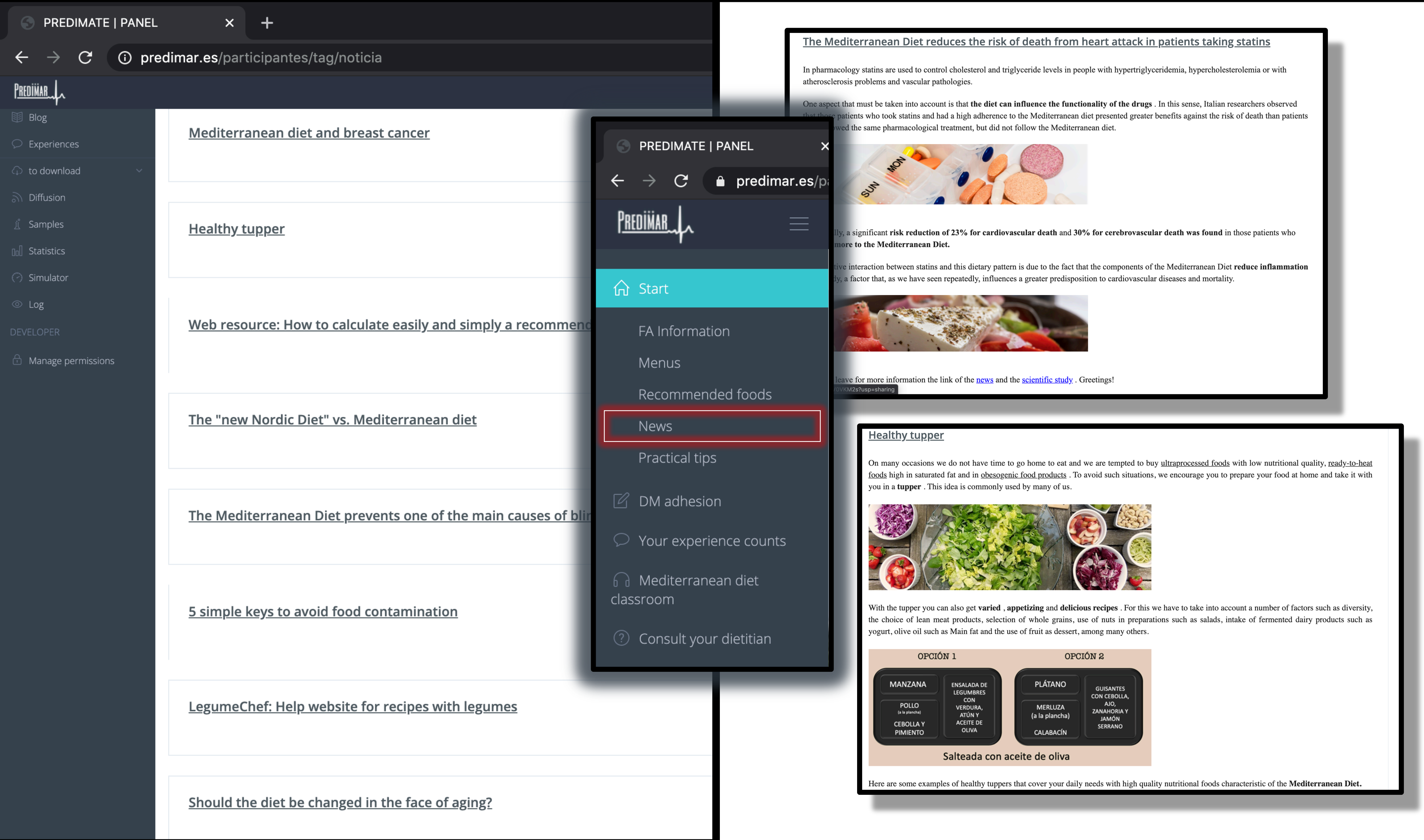

1. BACKGROUND OF HOME SCREEN: PRACTICAL TIPS

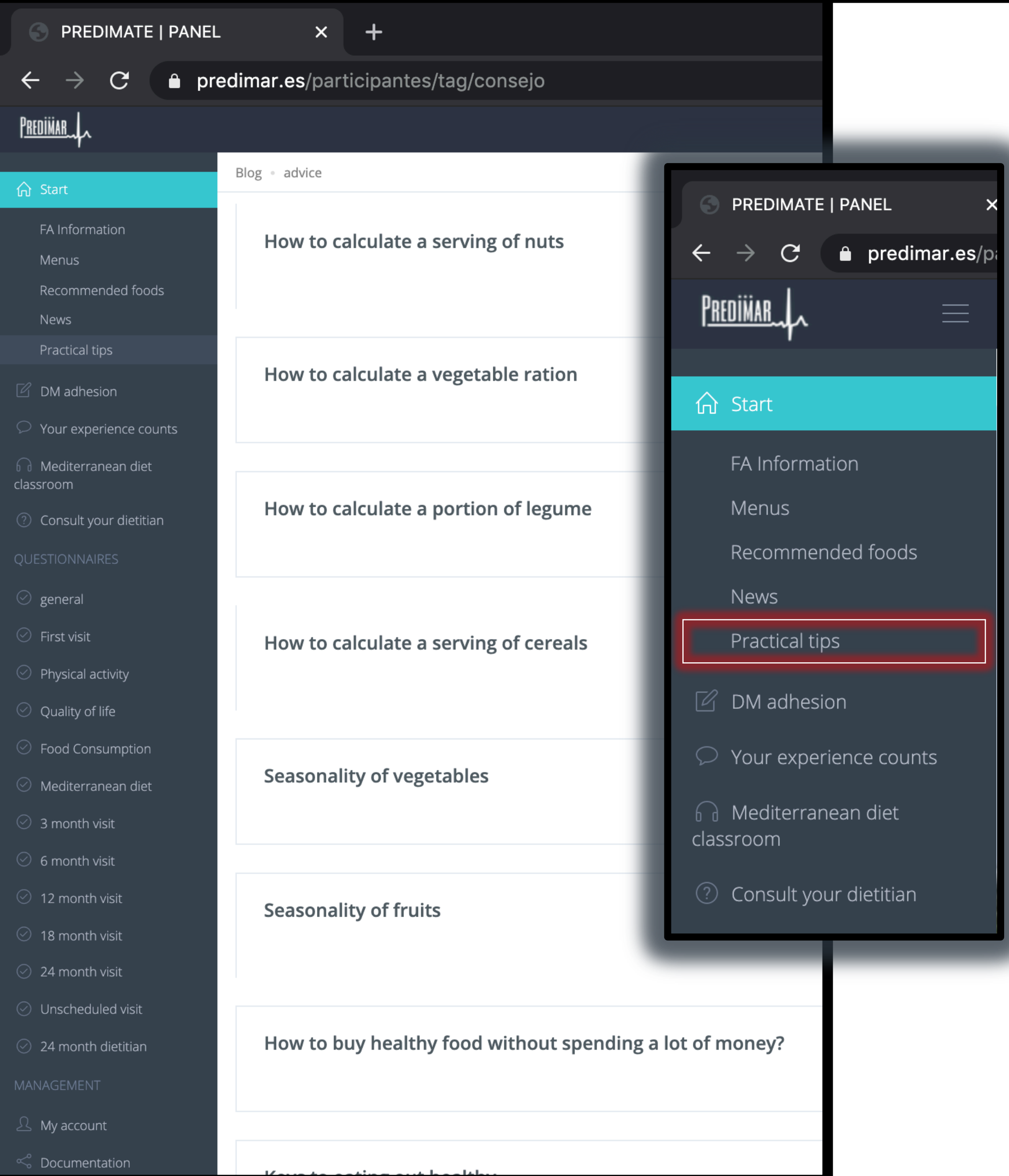

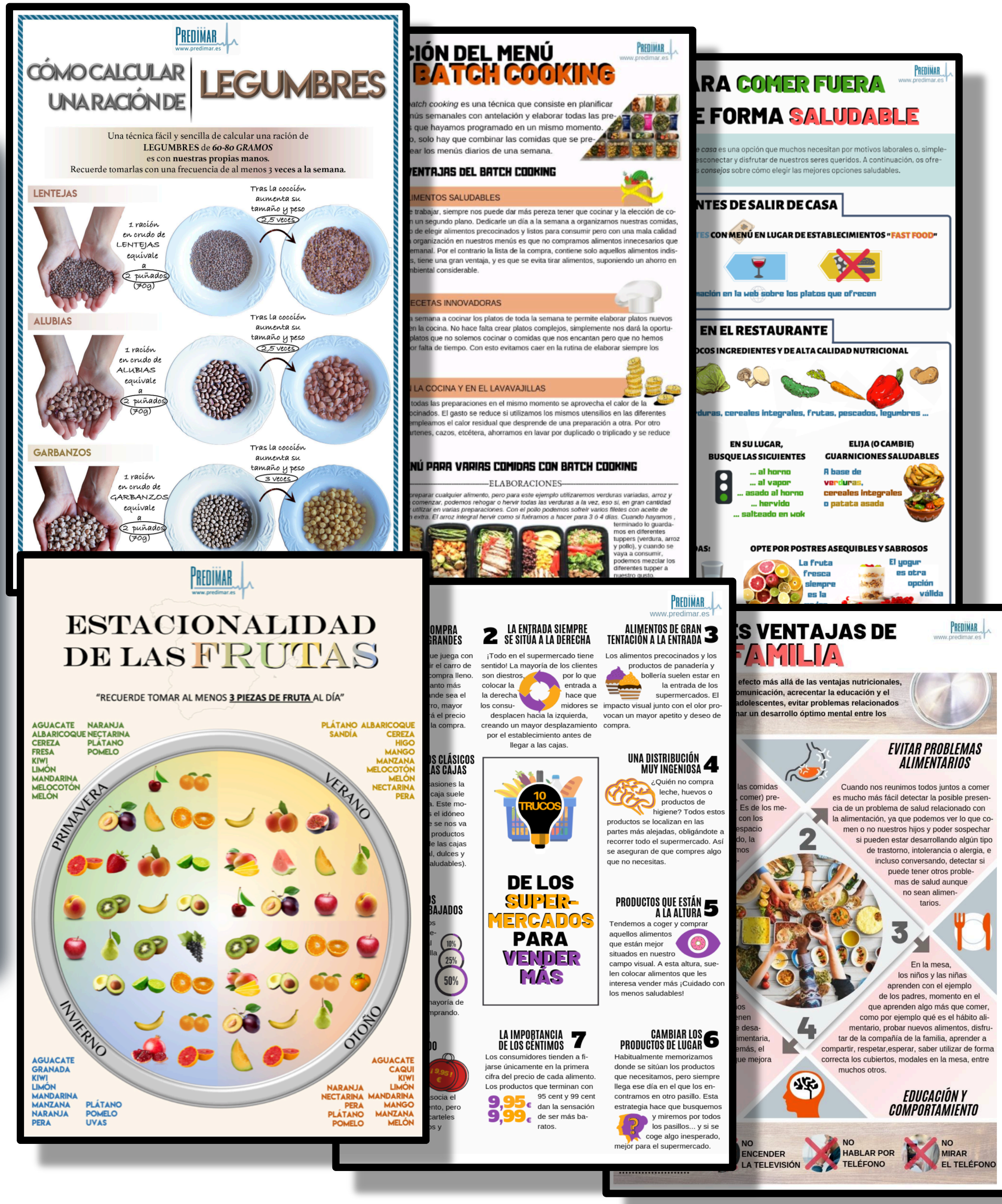

2. QUESTIONNAIRE OF ADHERENCE TO THE MEDITERRANEAN DIET

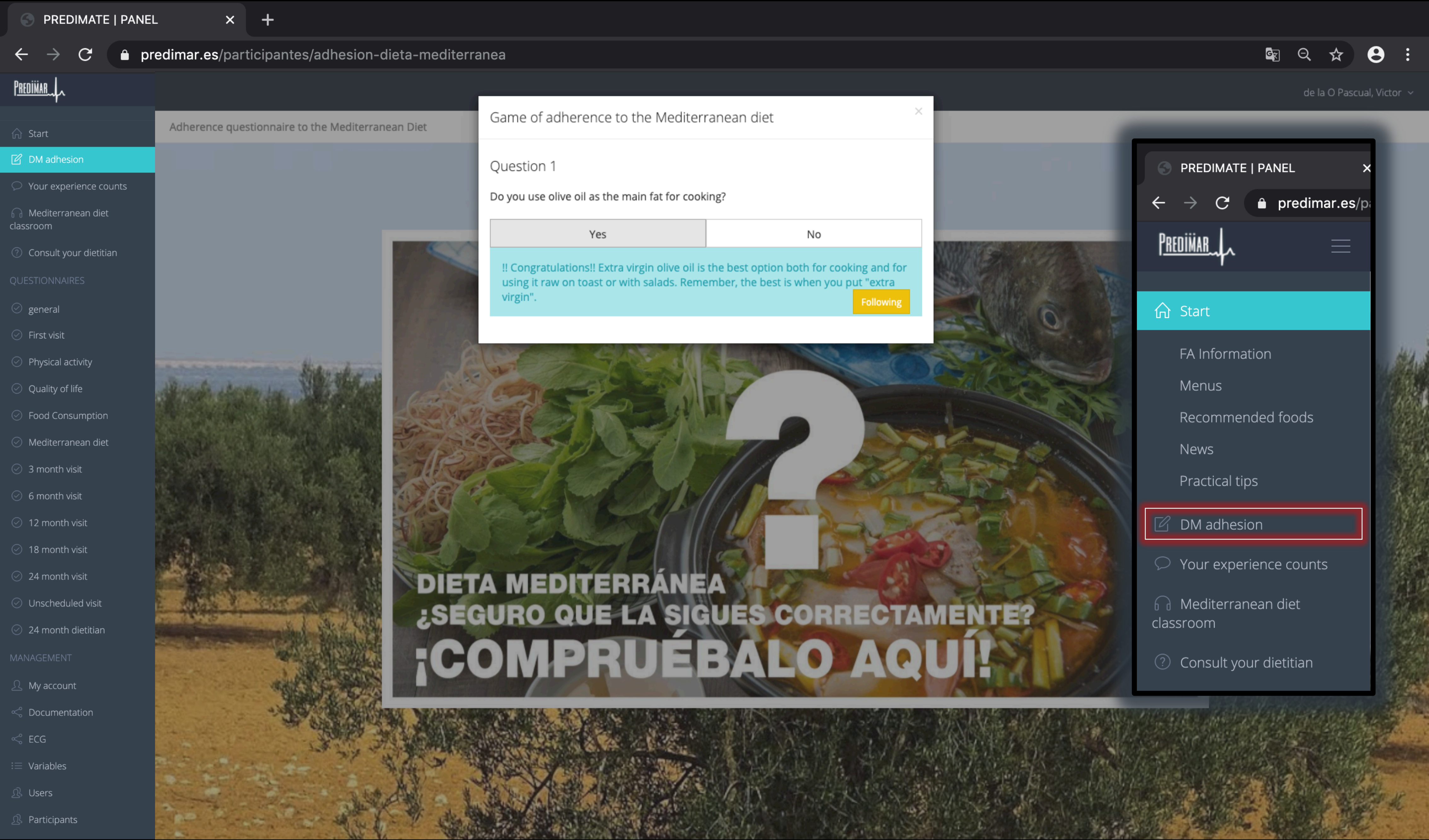

3. EXPERIENCES OF PARTICIPANTS

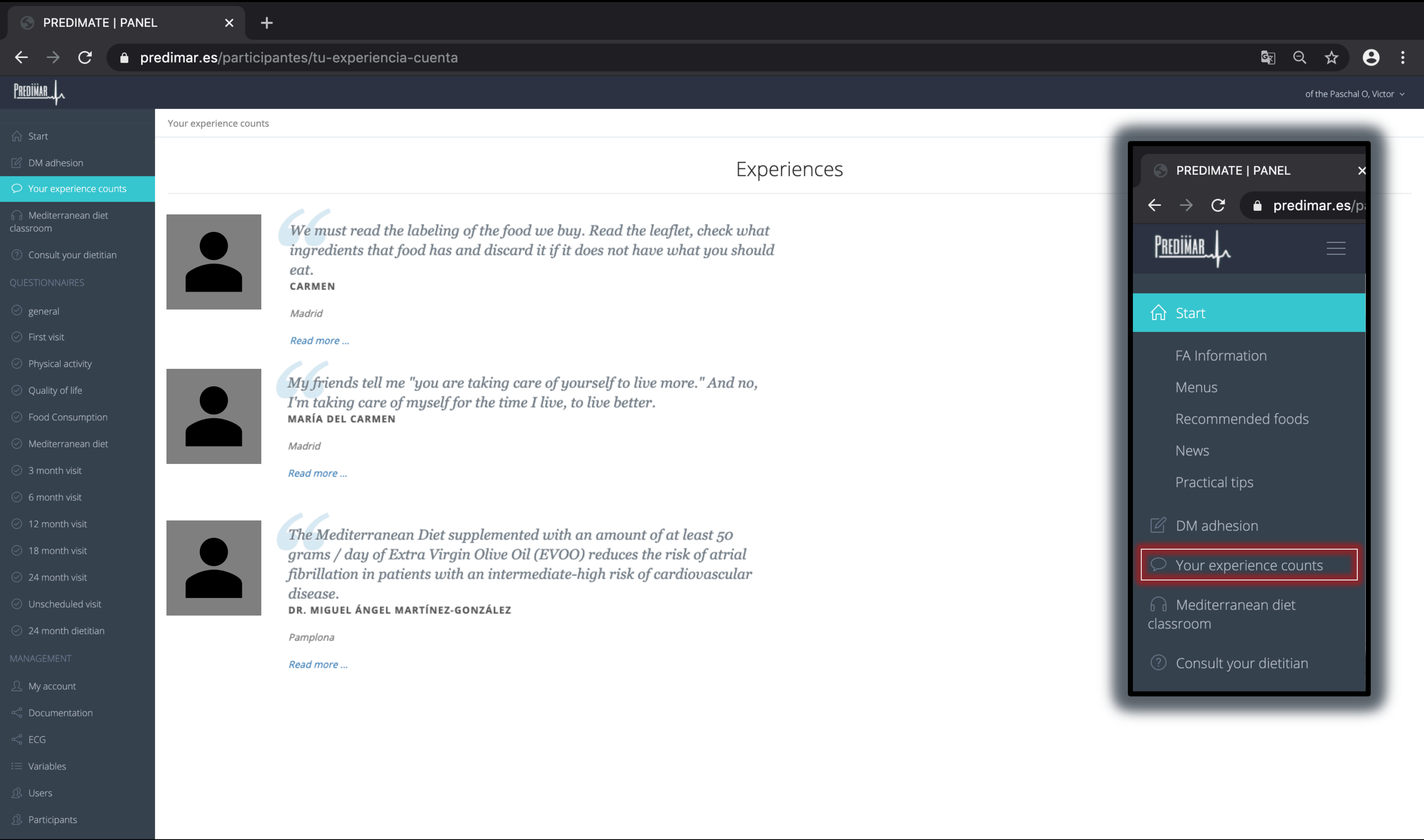

4. CLASSROOM OF MEDITERRANEAN DIET

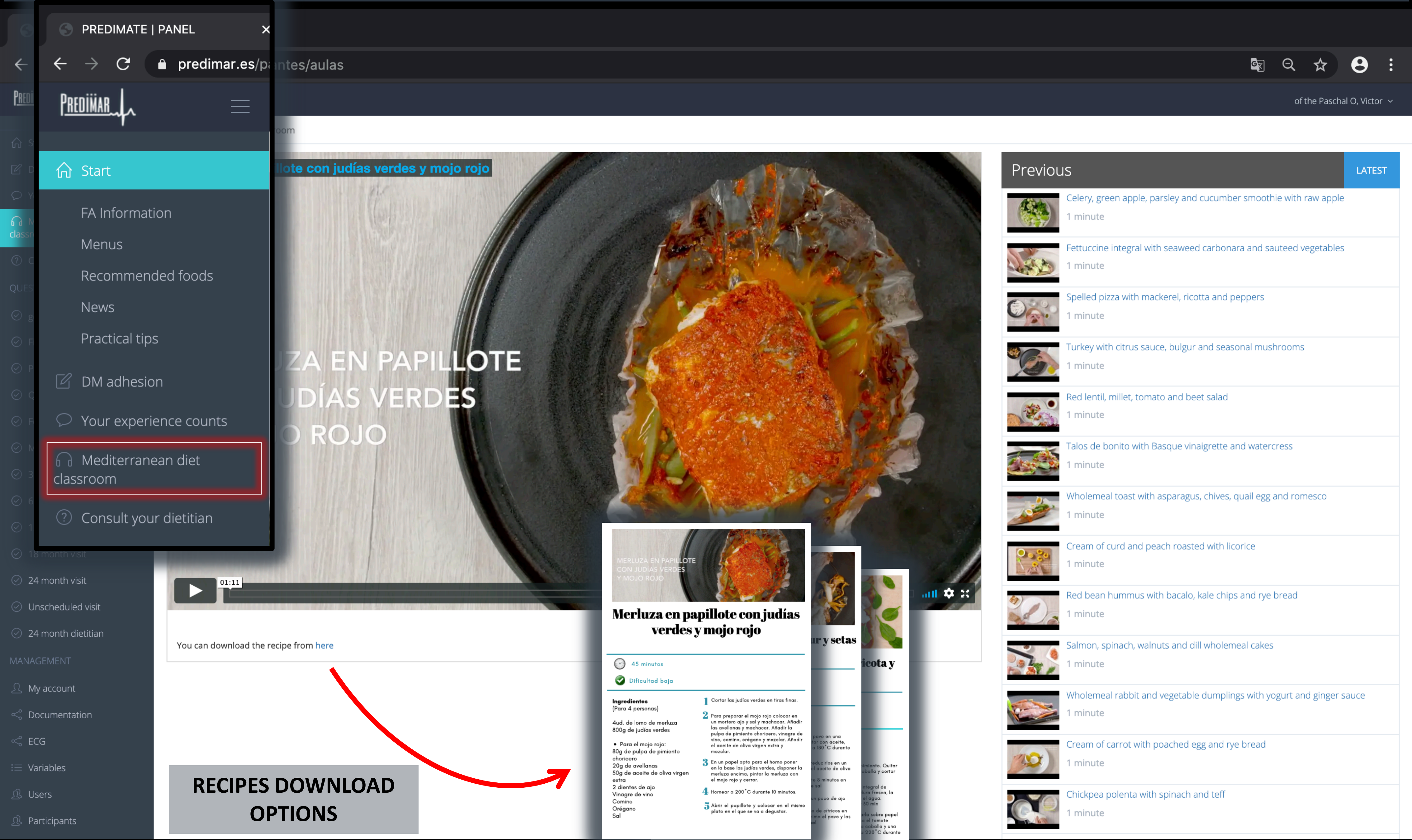

4. CLASSROOM OF MEDITERRANEAN DIET

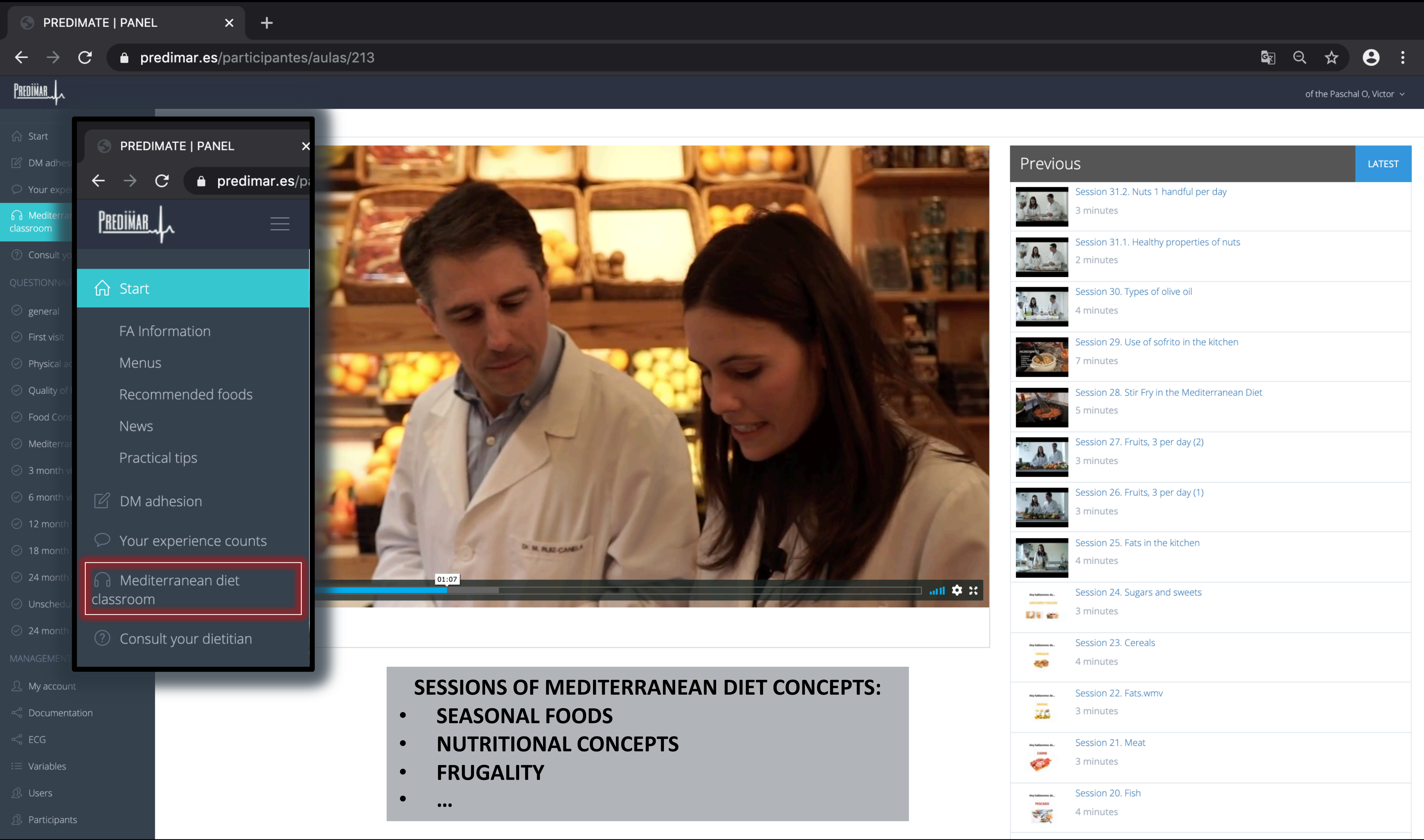

SESSIONS OF MEDITERRANEAN DIET CONCEPTS:

- **SEASONAL FOODS**
- **NUTRITIONAL CONCEPTS**
- **FRUGALITY**
- **...**

5. CONSULT YOUR DIETITIAN

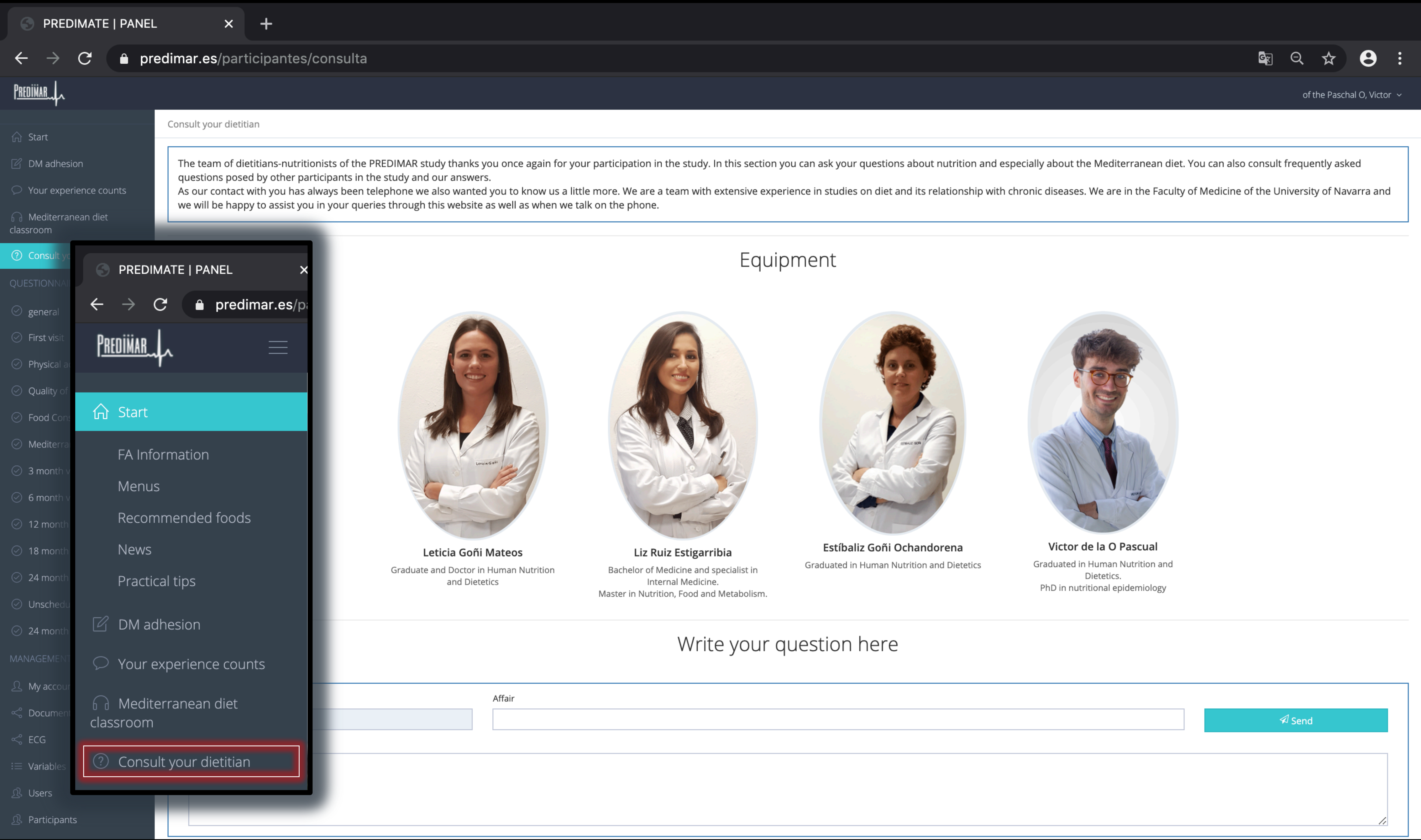

5. CONSULT YOUR DIETITIAN: FAQs

PREDIMATE | PANEL

predimar.es/participantes/consulta

PREDIMAR

of the Paschal O, Victor

Are ginger infusions recommended for my heart?

There is scientific evidence on the consumption of ginger and cardiovascular diseases. In fact, a recent publication concludes that ginger consumption lowers triglyceride levels and LDL cholesterol (or bad cholesterol) in the blood. In addition, the cardioprotective effect of ginger consumption could be related to its antioxidant properties, inhibition of oxidative stress, hypotensive and anti-atherosclerotic mechanisms (preventing the formation of a plaque of fat and other substances in the arteries).
However, as far as we know there is limited evidence about the effect of ginger in the case of atrial fibrillation. In this sense, a study indicates that in patients with non-valvular atrial fibrillation treated with Warfari [illegible] her herbs, would be contraindicated. This is because its consumption complicates the anticoagulant control of the drug.
If we take into account the cardioprotective effect of ginger consumption, its consumption may be recommended. However, some anticoagulant treatments that are used to treat atrial fibrillation, such as the synt [illegible] ore, if you take ginger or if you are considering doing so, we suggest that you discuss it with your cardiologist.

Can you make homemade desserts when you replace the unhealthy ingredients with more beneficial ones?

When we have a meal with family or friends, a problem is usually the choice of dessert since they are usually rich in sugars and saturated (unhealthy) fats and therefore are not included in the Mediterranean diet [illegible]

A possible option is to make homemade desserts in which sugar is replaced by artificial or natural sweeteners (add dried fruits such as dates, raisins), and unhealthy fats such as butter or margarine for healthier [illegible] ays make homemade desserts based on fresh fruit.

For example, you can make rice pudding at home using sweetener instead of sugar and skim milk instead of whole milk. In this way, excessive consumption of calories and consumption of unhealthy foods such as [illegible] followed, the calories provided by rice as cereal should be taken into account, so on that day you should control your consumption, for example, of bread and other cereals such as pasta or potatoes.

The use of homemade desserts in which sugars and low-fat fats are substituted can occasionally be taken within the Mediterranean diet pattern as long as they do not displace the consumption of fresh fruit. That [illegible] out the day, you can have homemade rice pudding for lunch or dinner.

Fruit, before or after meals

The idea that you have to eat the fruit before meals usually is based on the fact that this will make us less fat. However, all foods provide us with the same number of calories regardless of the time of day we take [illegible] l both before and after eating. Of course, drinking the fruit before eating can have a satiating effect due to its high fiber content. If we feel fuller, this will usually make us take smaller portions of the prepared dishes at th [illegible]

A danger of taking the fruit before is that later in the meals it is replaced by other less healthy desserts for its greater content in sugars and fats (custard, rice pudding, cakes). Therefore we recommend that, prefer [illegible]

Do nuts favor the appearance of migraine sprouts?

Migraine can be triggered by various causes including stress, hormonal factors, food allergies, intake of certain drugs, hypoglycemia (low blood sugar levels) or lack of sleep. However, most cases of migraine are re [illegible] se or DAO) that is responsible for metabolizing (digesting) the histamine present in food. This deficit is caused by various causes including genetic factors, consumption of certain drugs or inflammatory bowel diseases. Histan [illegible] d and tissues producing migraines among other symptoms.

There are foods that can give us a greater amount of histamine. Among foods rich in histamines are fermented or ripened foods such as cured raw meat derivatives (chorizo, salchichón, salami, fuer, sobrasada), m [illegible] l, gruyère, roquefort, camembert), canned fish (tuna, sardine, mackerel, anchovies, herring), fermented vegetable products (sauerkraut, soy derivatives), fermented alcoholic beverages (wine, beer). There are also some comp [illegible] ods such as seafood, nuts, egg white, chocolate, some fruits (banana, strawberries, pineapple) and vegetables (tomato, spinach). Although foods rich in histamine and other compounds that may affect their levels have bee [illegible] rect relationship between the consumption of certain foods and headache. We must also bear in mind that migraine appears when histamine has accumulated over time and not because of the occasional consumptio [illegible]

In summary, we cannot objectively conclude that the consumption of nuts favors the appearance of migraines. Our opinion is that the consumption of 30g of nuts at least 3 times a week should not be related to t [illegible]

If you have migraines, we recommend you avoid fasting, reduce stress situations, sleep enough hours, avoid low blood pressure and glucose and moderate caffeine consumption. Regarding the diet, we would recommend not abusing those foods considered rich in histamine in the same day. Following the Mediterranean Diet we ensure a varied consumption of food and also the consumption of some of the foods rich in histamine such as cured raw meat derivatives or matured cheeses is also limited.

Can canned fish be consumed?

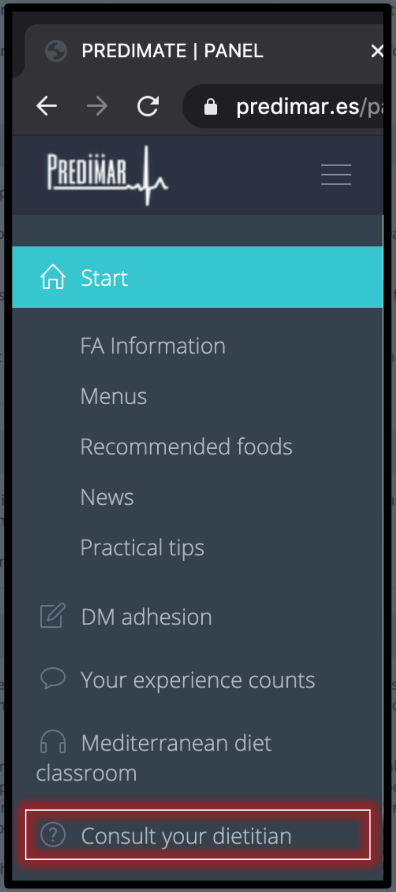

5. STATISTICS OF PARTICIPANTS RECRUITMENT

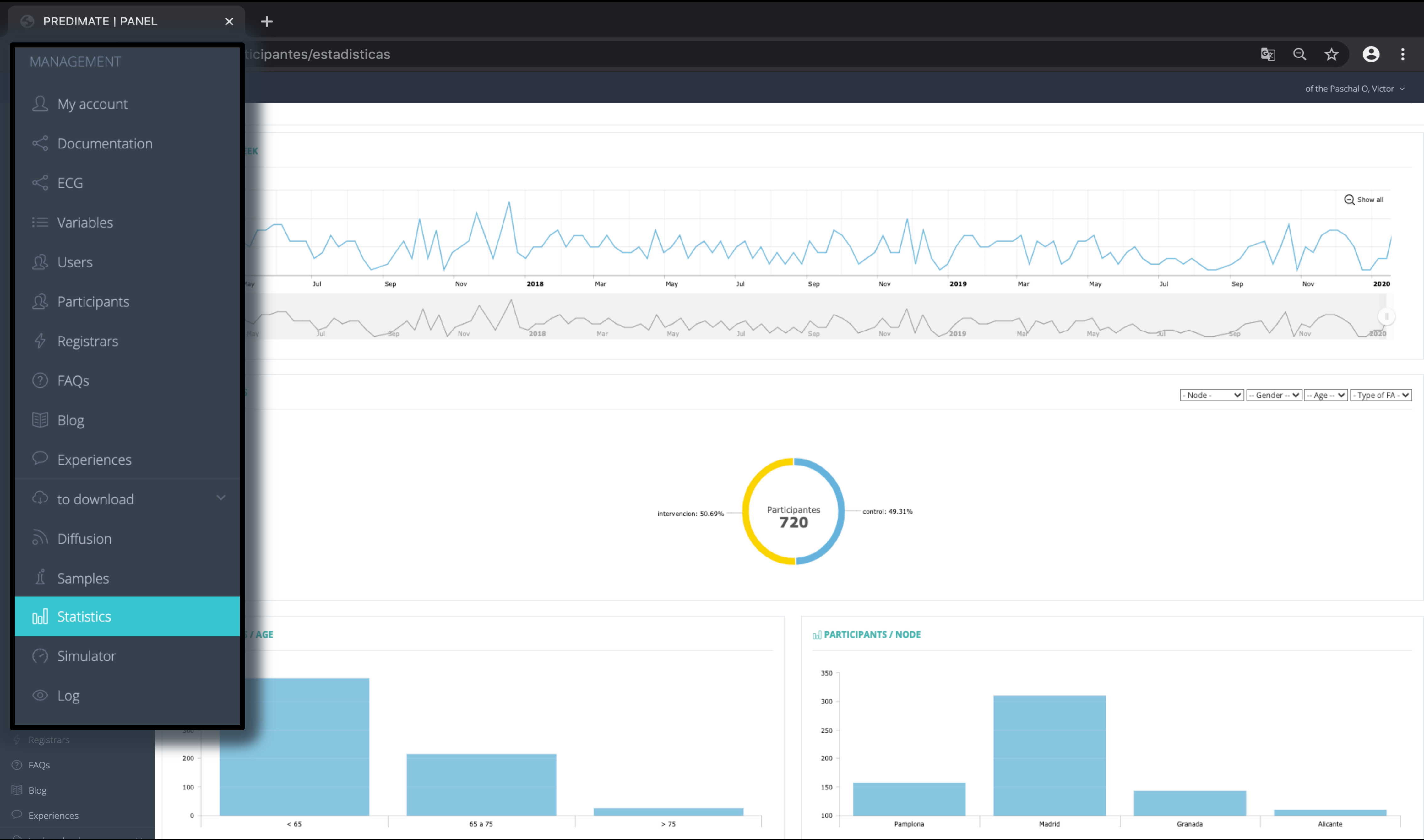

Supplement: Multimedia Appendix 3 [file jmir_v22i12e21436_app3.pdf]
