# Supplementary material for: A Remote Nutritional Intervention to Change the Dietary Habits of Patients Undergoing Ablation of Atrial Fibrillation: Randomized Controlled Trial
Source: J Med Internet Res. 2020 Dec 7;22(12):e21436. doi: 10.2196/21436 (PMC7752535; doi:10.2196/21436)

Multimedia Appendix 4

Screenshots of the Predimar app

GooglePlay | AppStore

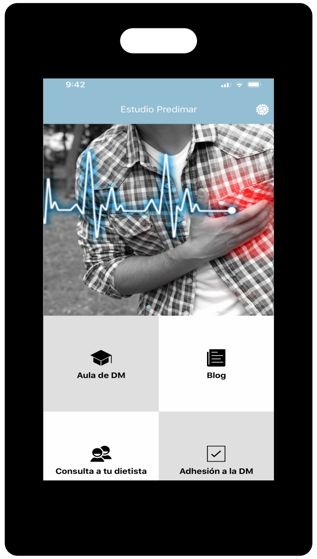

1. MOBILE AND TABLET STORE APP

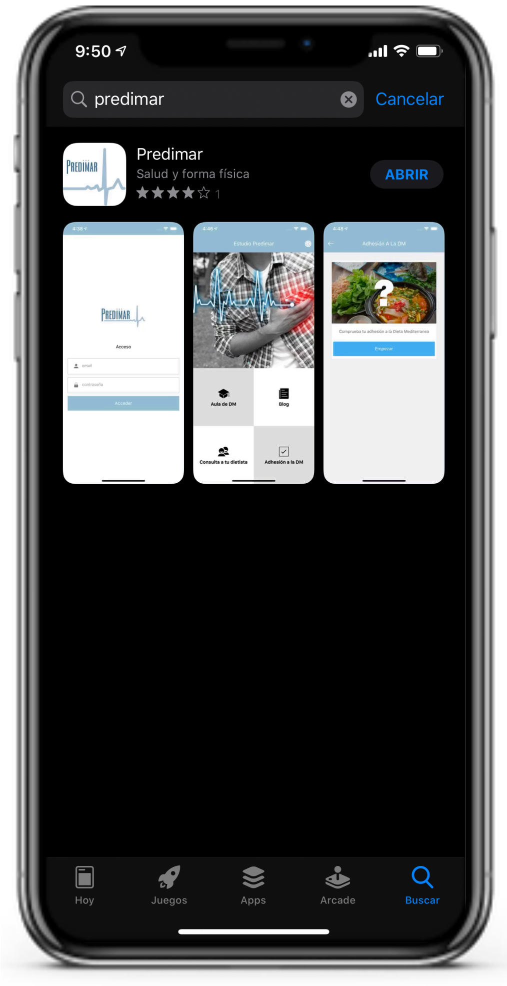

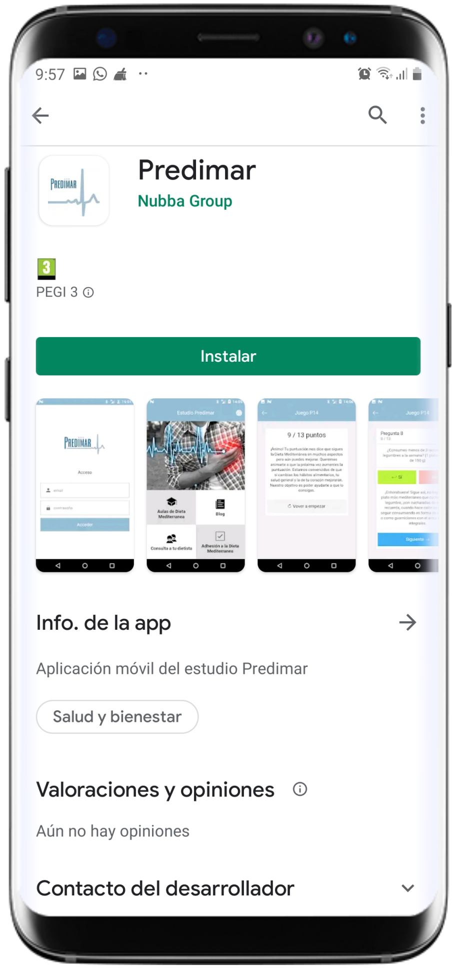

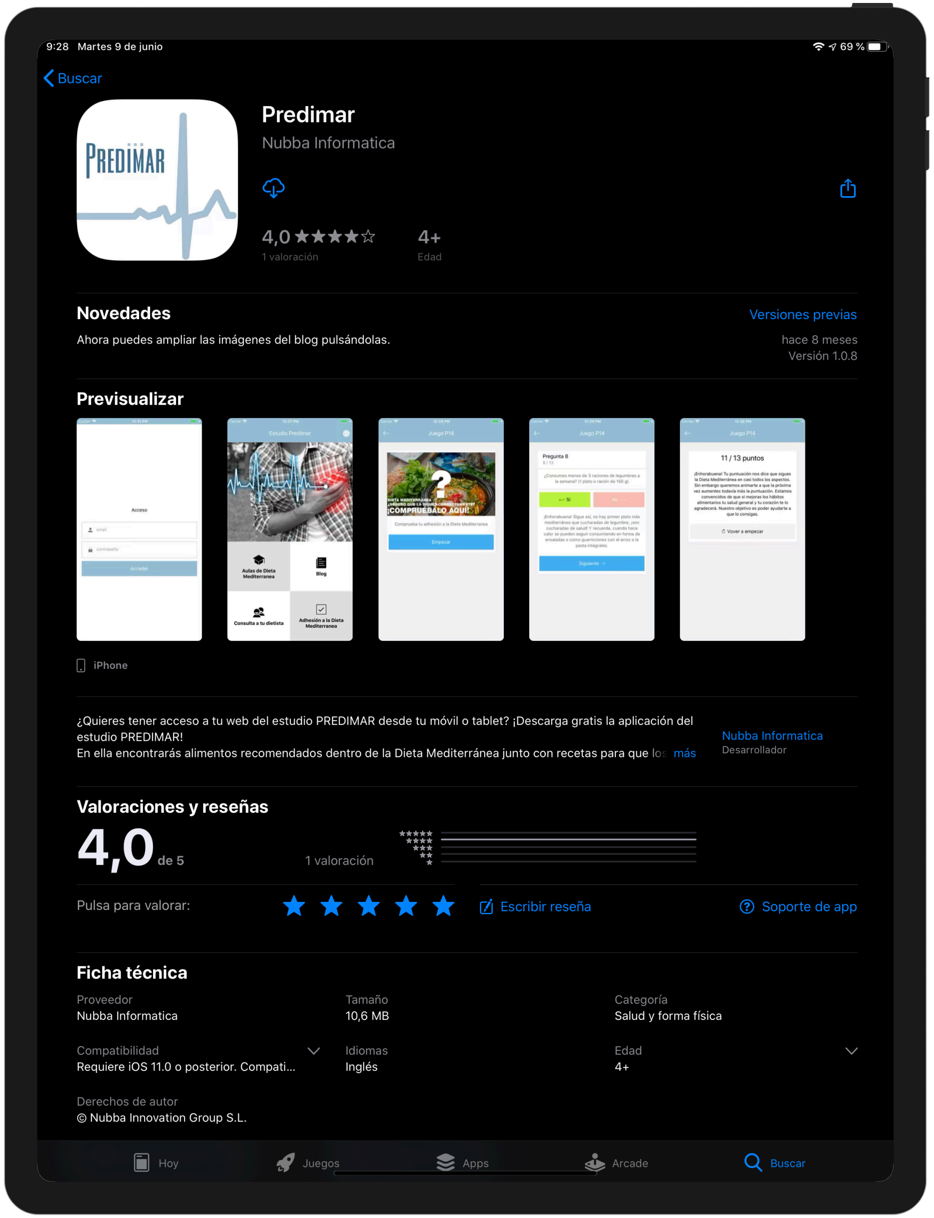

2. LOG IN AND FOREGROUND OF PREDIMAR APP

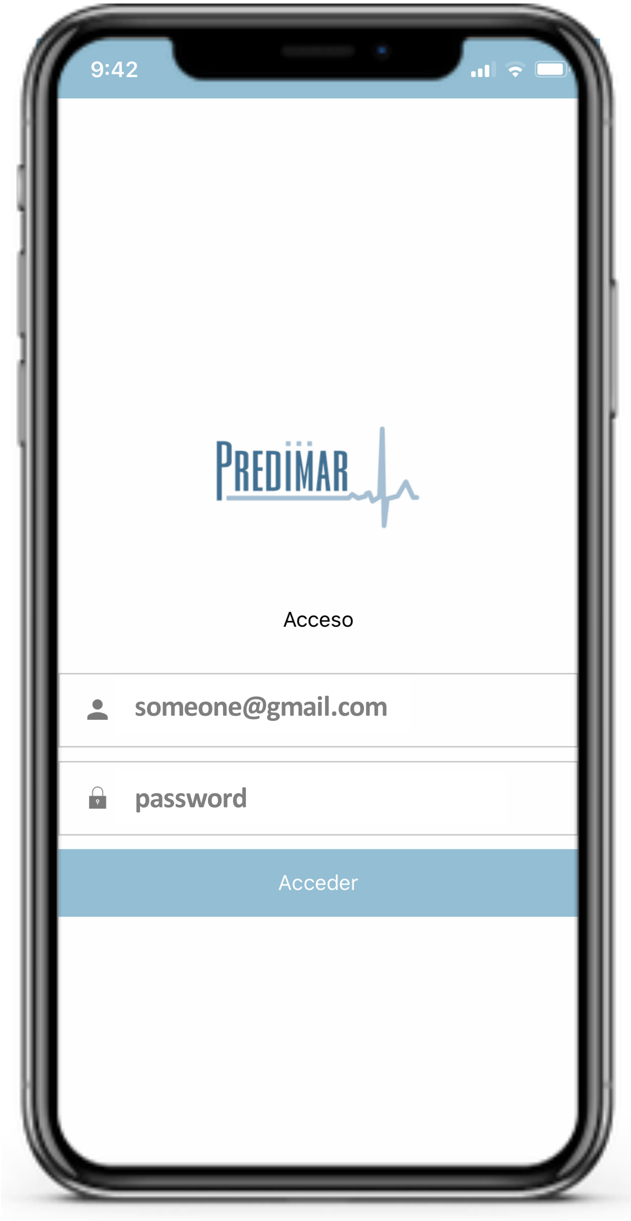

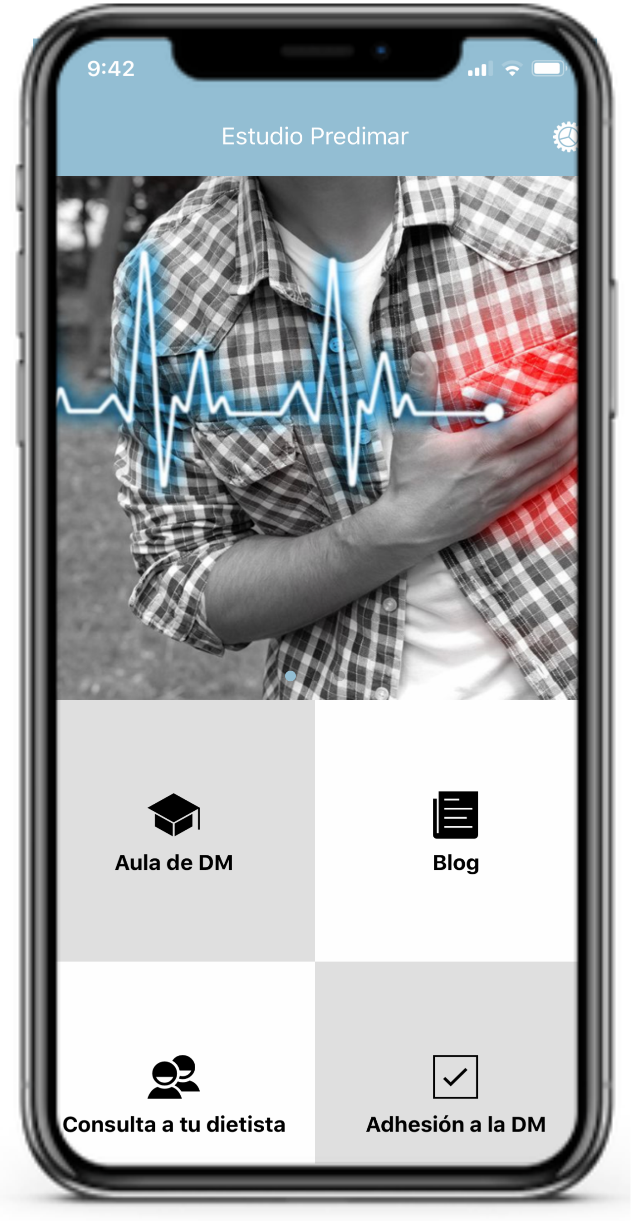

3. CLASSROOM OF MEDITERRANEAN DIET

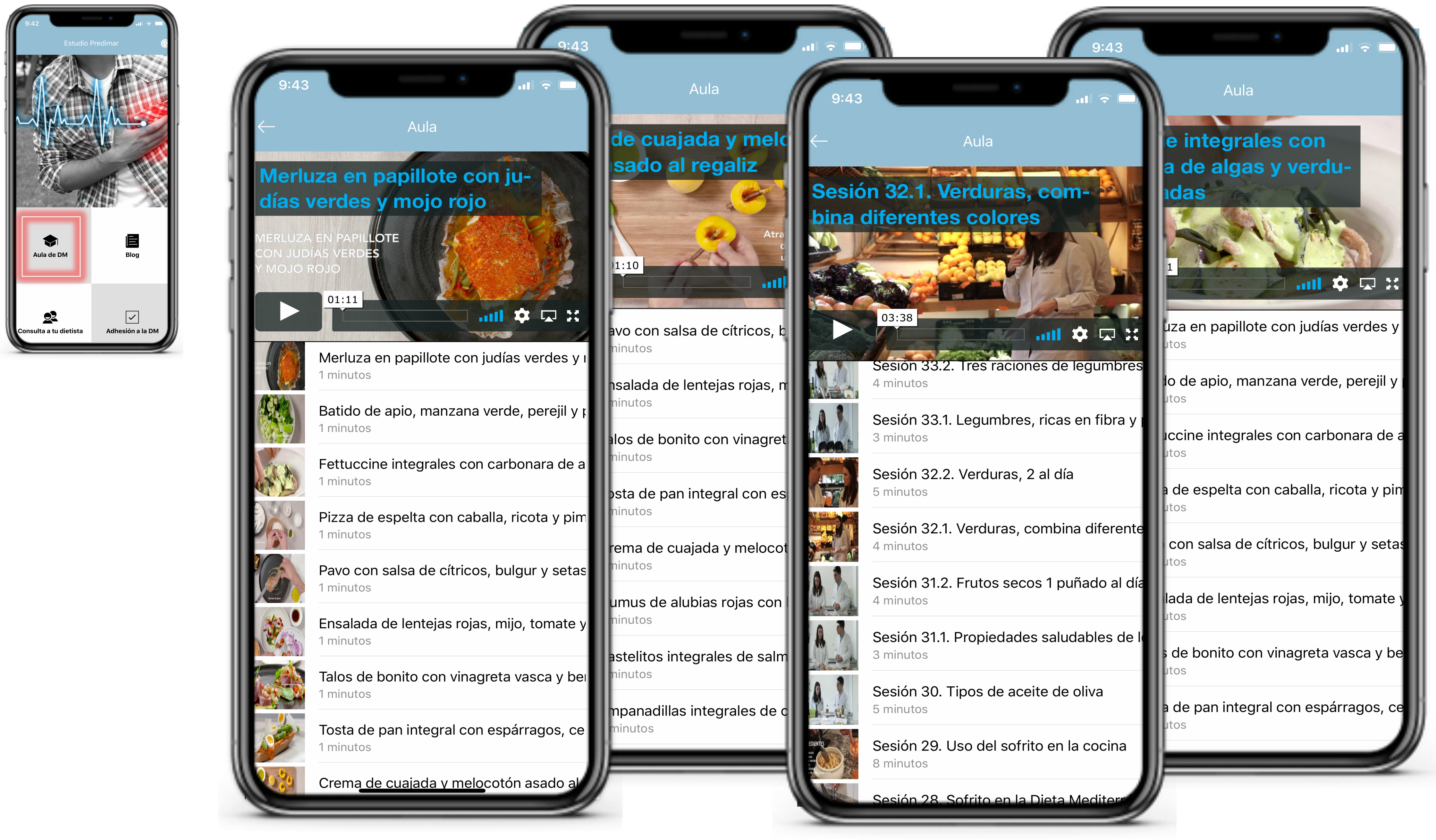

4. BLOG: RECOMMENDED FOODS

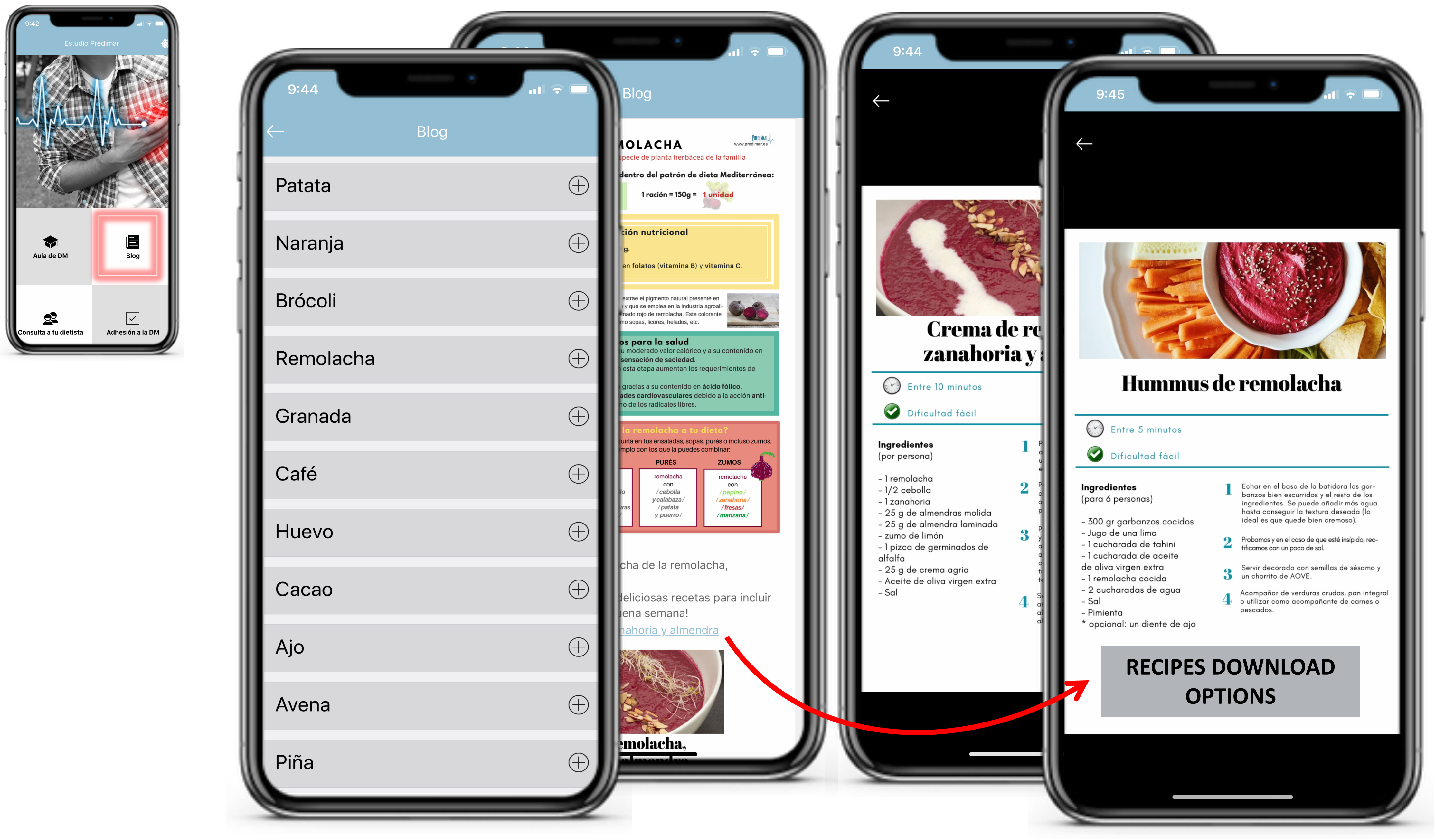

4. BLOG: NEWS

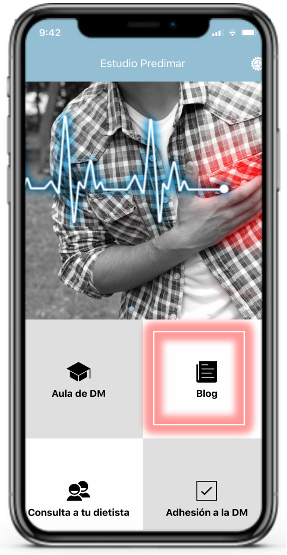
9:42
Estudio Predimar
Aula de DM
Blog
Consulta a tu dietista
Adhesión a la DM

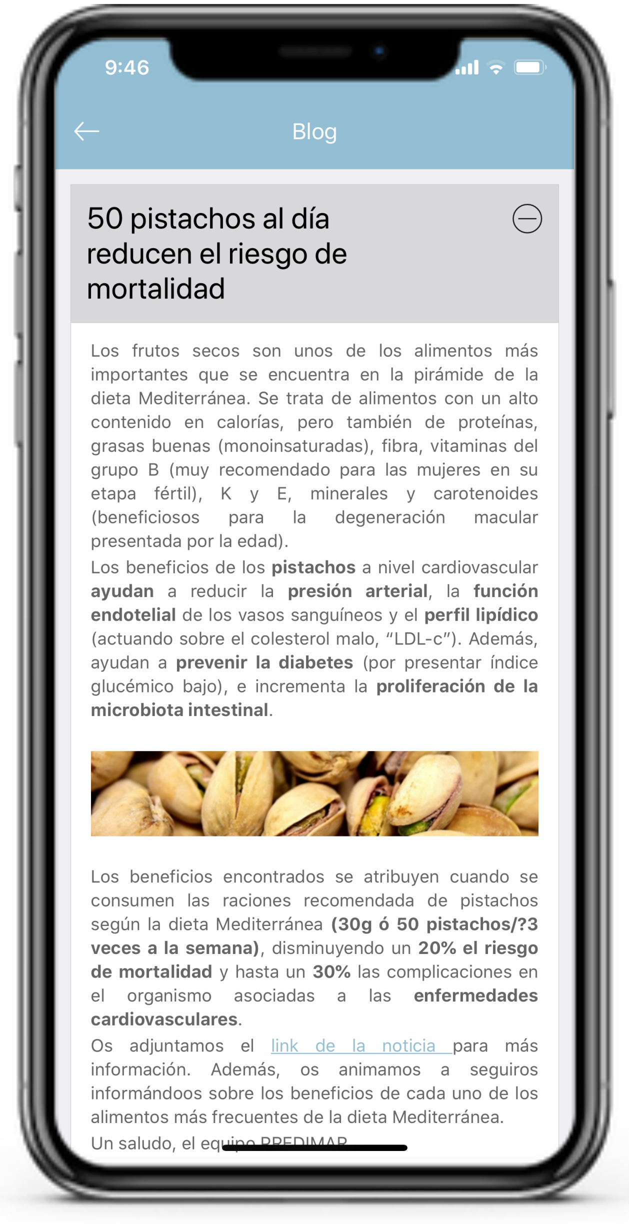
9:46
Blog
50 pistachos al día reducen el riesgo de mortalidad
Los frutos secos son unos de los alimentos más importantes que se encuentra en la pirámide de la dieta Mediterránea. Se trata de alimentos con un alto contenido en calorías, pero también de proteínas, grasas buenas (monoinsaturadas), fibra, vitaminas del grupo B (muy recomendado para las mujeres en su etapa fértil), K y E, minerales y carotenoides (beneficiosos para la degeneración macular presentada por la edad).
Los beneficios de los pistachos a nivel cardiovascular ayudan a reducir la presión arterial, la función endotelial de los vasos sanguíneos y el perfil lipídico (actuando sobre el colesterol malo, "LDL-c"). Además, ayudan a prevenir la diabetes (por presentar índice glucémico bajo), e incrementa la proliferación de la microbiota intestinal.
Los beneficios encontrados se atribuyen cuando se consumen las raciones recomendada de pistachos según la dieta Mediterránea (30g ó 50 pistachos/?3 veces a la semana), disminuyendo un 20% el riesgo de mortalidad y hasta un 30% las complicaciones en el organismo asociadas a las enfermedades cardiovasculares.
Os adjuntamos el link de la noticia para más información. Además, os animamos a seguiros informándoos sobre los beneficios de cada uno de los alimentos más frecuentes de la dieta Mediterránea.
Un saludo, el equipo PREDIMAR

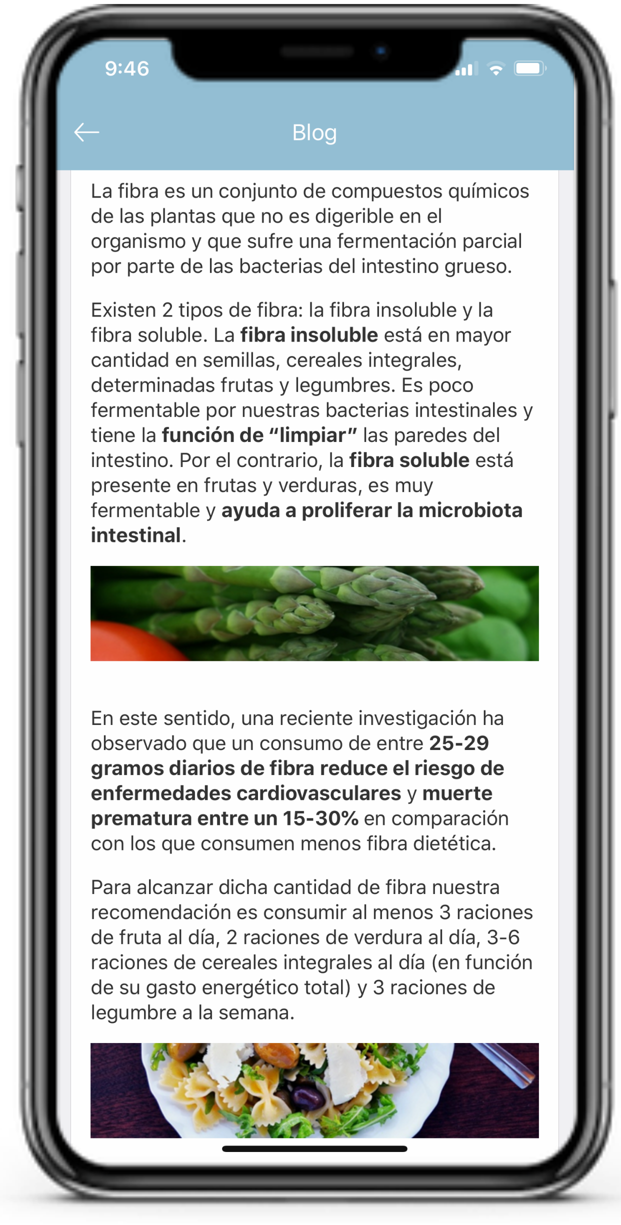
9:46
Blog
La fibra es un conjunto de compuestos químicos de las plantas que no es digerible en el organismo y que sufre una fermentación parcial por parte de las bacterias del intestino grueso.
Existen 2 tipos de fibra: la fibra insoluble y la fibra soluble. La fibra insoluble está en mayor cantidad en semillas, cereales integrales, determinadas frutas y legumbres. Es poco fermentable por nuestras bacterias intestinales y tiene la función de "limpiar" las paredes del intestino. Por el contrario, la fibra soluble está presente en frutas y verduras, es muy fermentable y ayuda a proliferar la microbiota intestinal.
En este sentido, una reciente investigación ha observado que un consumo de entre 25-29 gramos diarios de fibra reduce el riesgo de enfermedades cardiovasculares y muerte prematura entre un 15-30% en comparación con los que consumen menos fibra dietética.
Para alcanzar dicha cantidad de fibra nuestra recomendación es consumir al menos 3 raciones de fruta al día, 2 raciones de verdura al día, 3-6 raciones de cereales integrales al día (en función de su gasto energético total) y 3 raciones de legumbre a la semana.

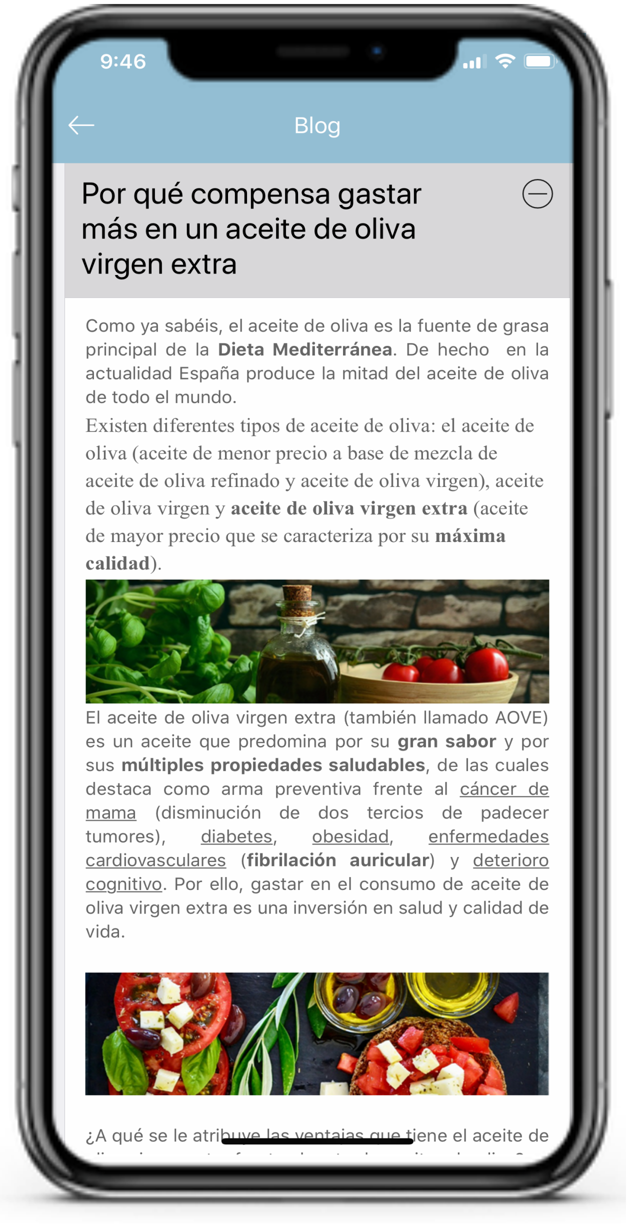
9:46
Blog
Por qué compensa gastar más en un aceite de oliva virgen extra
Como ya sabéis, el aceite de oliva es la fuente de grasa principal de la Dieta Mediterránea. De hecho en la actualidad España produce la mitad del aceite de oliva de todo el mundo.
Existen diferentes tipos de aceite de oliva: el aceite de oliva (aceite de menor precio a base de mezcla de aceite de oliva refinado y aceite de oliva virgen), aceite de oliva virgen y aceite de oliva virgen extra (aceite de mayor precio que se caracteriza por su máxima calidad).
El aceite de oliva virgen extra (también llamado AOVE) es un aceite que predomina por su gran sabor y por sus múltiples propiedades saludables, de las cuales destaca como arma preventiva frente al cáncer de mama (disminución de dos tercios de padecer tumores), diabetes, obesidad, enfermedades cardiovasculares (fibrilación auricular) y deterioro cognitivo. Por ello, gastar en el consumo de aceite de oliva virgen extra es una inversión en salud y calidad de vida.
¿A qué se le atribuye las ventajas que tiene el aceite de

4. BLOG: MONTHLY MENUS

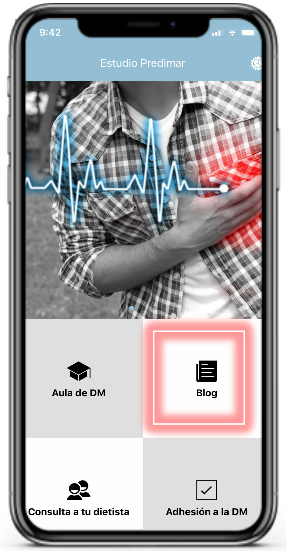

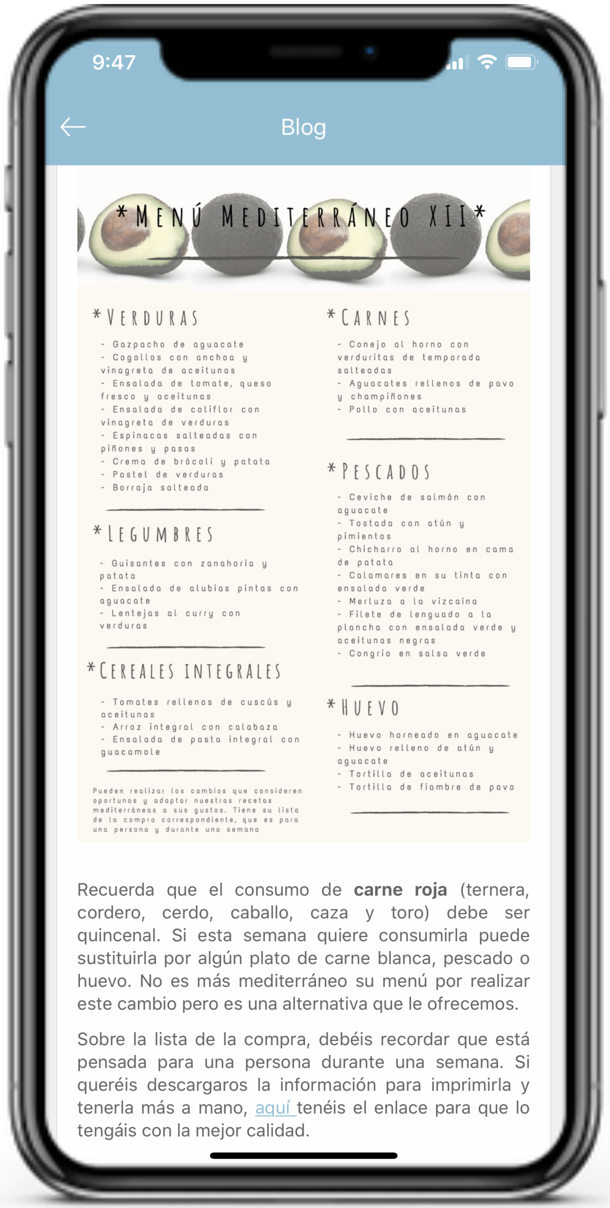

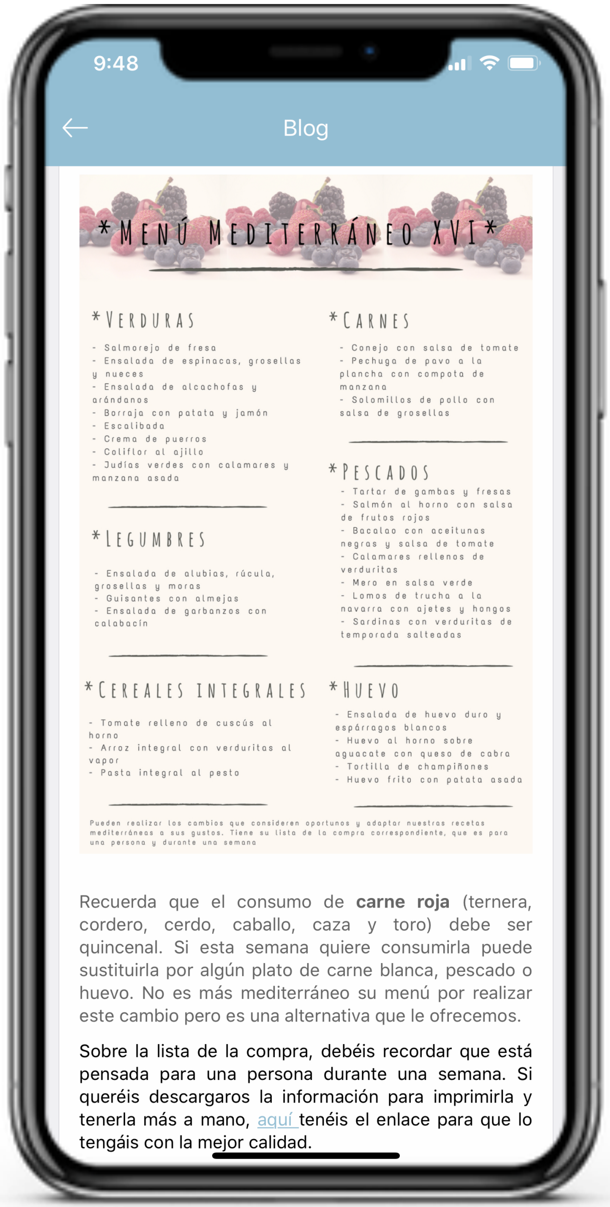

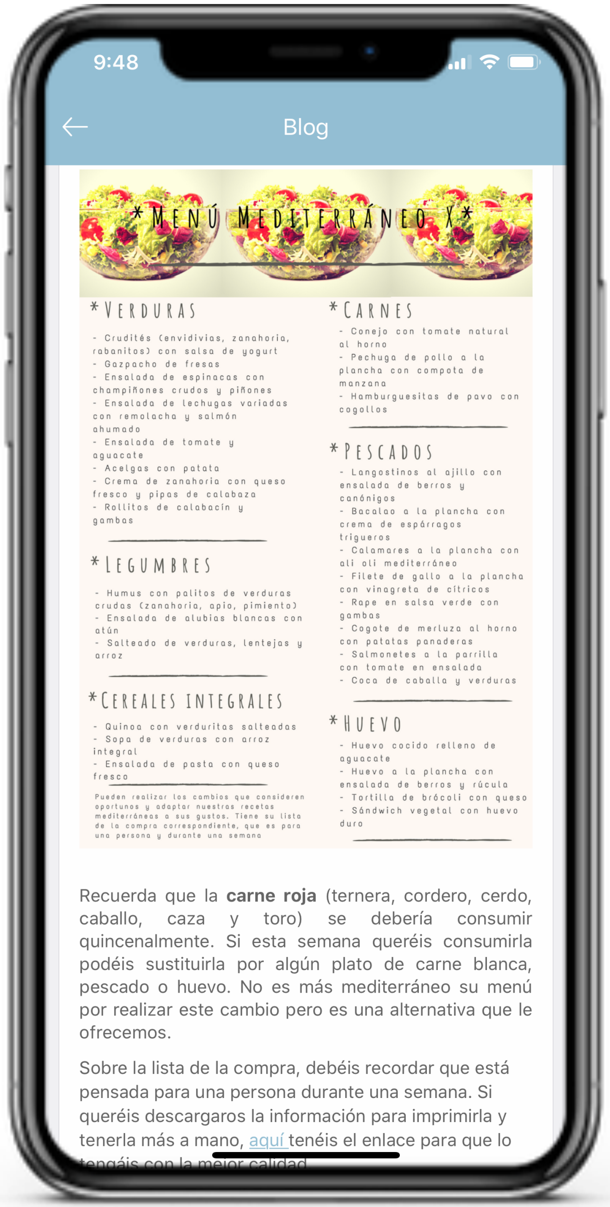

4. BLOG: PRACTICAL TIPS

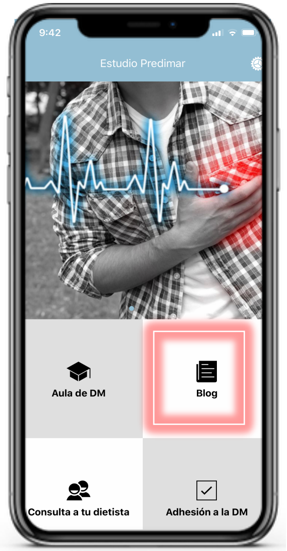

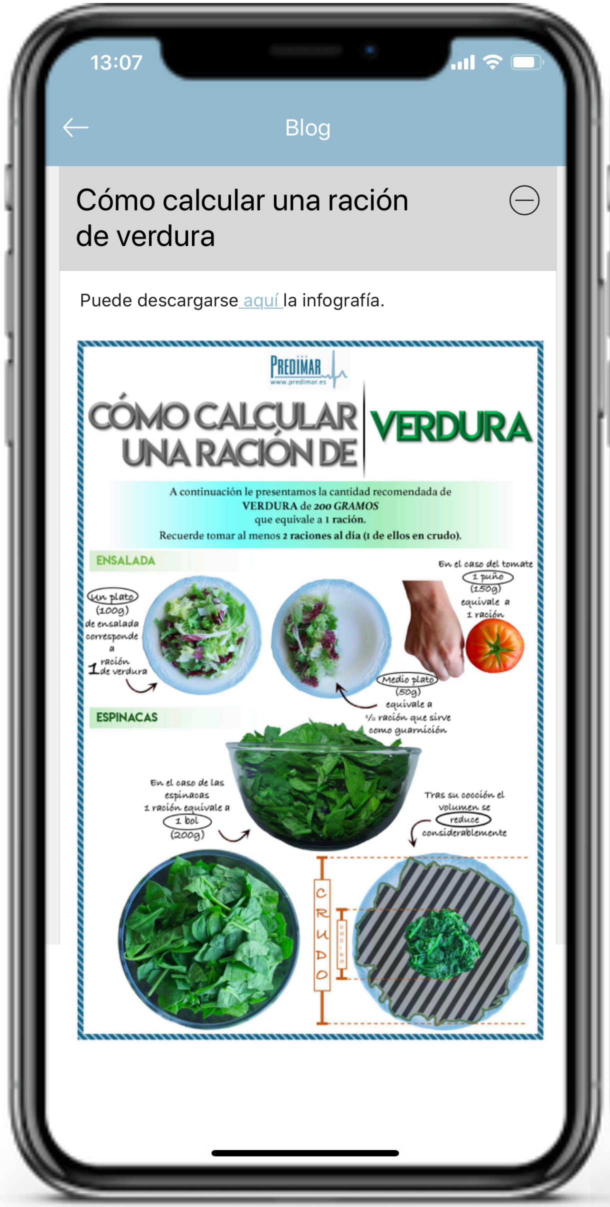

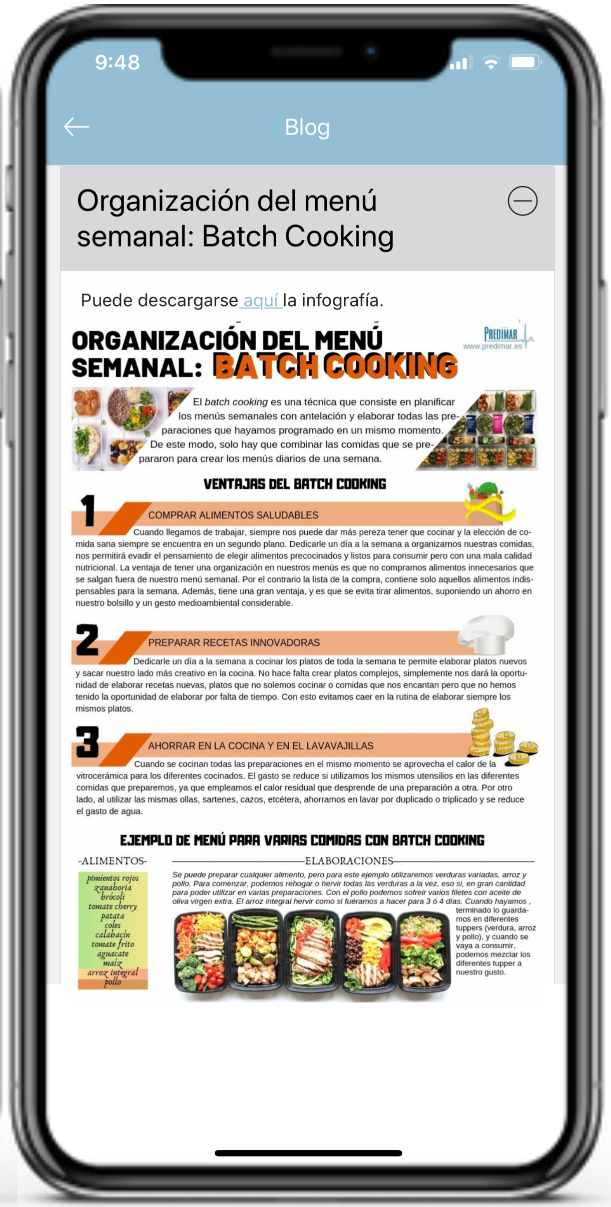

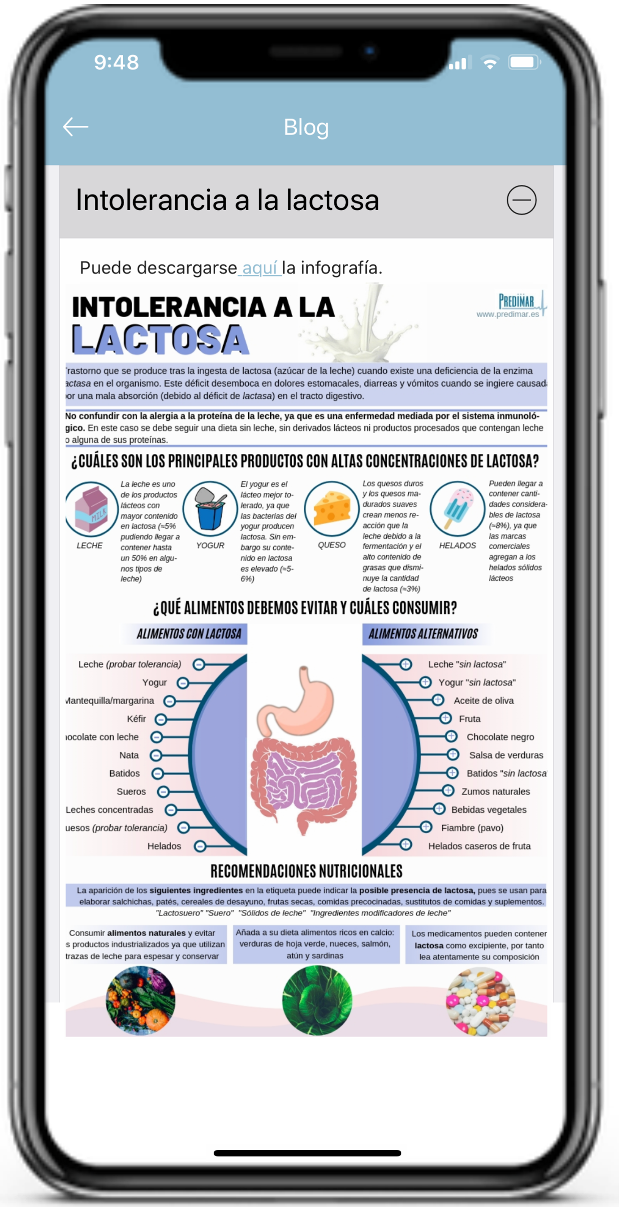

5. CONSULT YOUR DIETITIAN

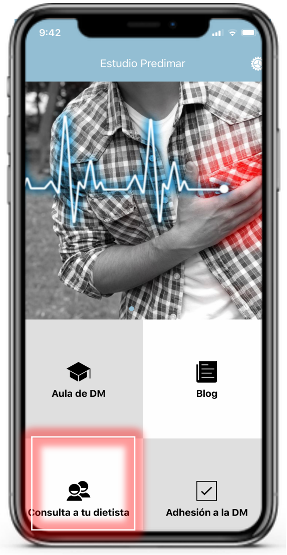

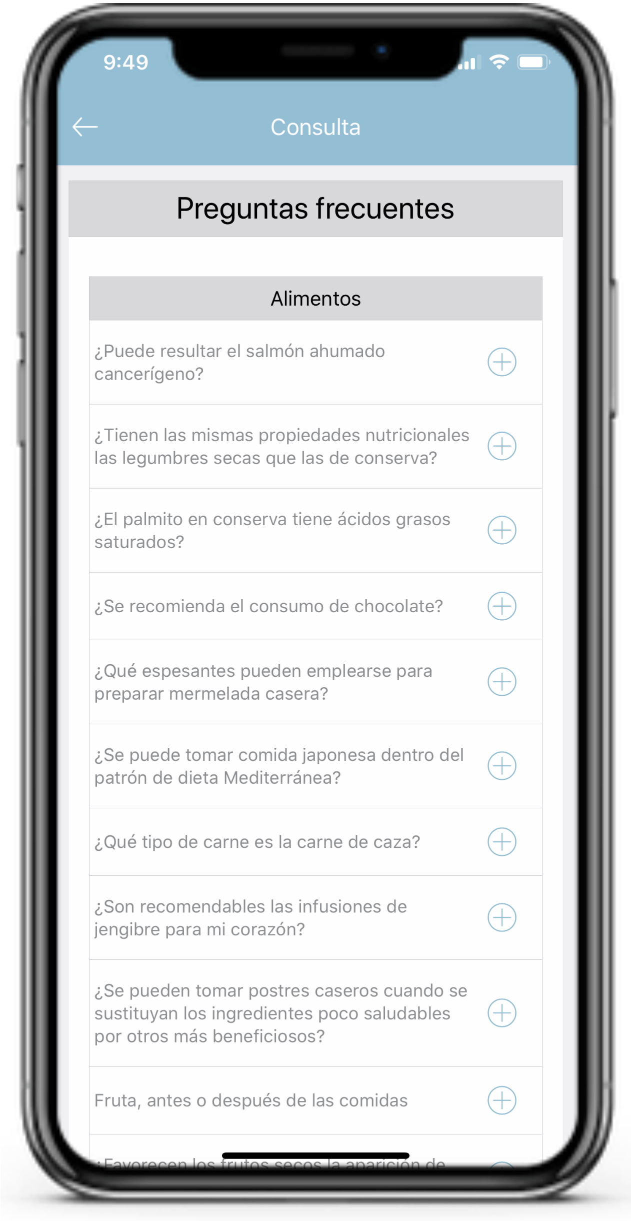

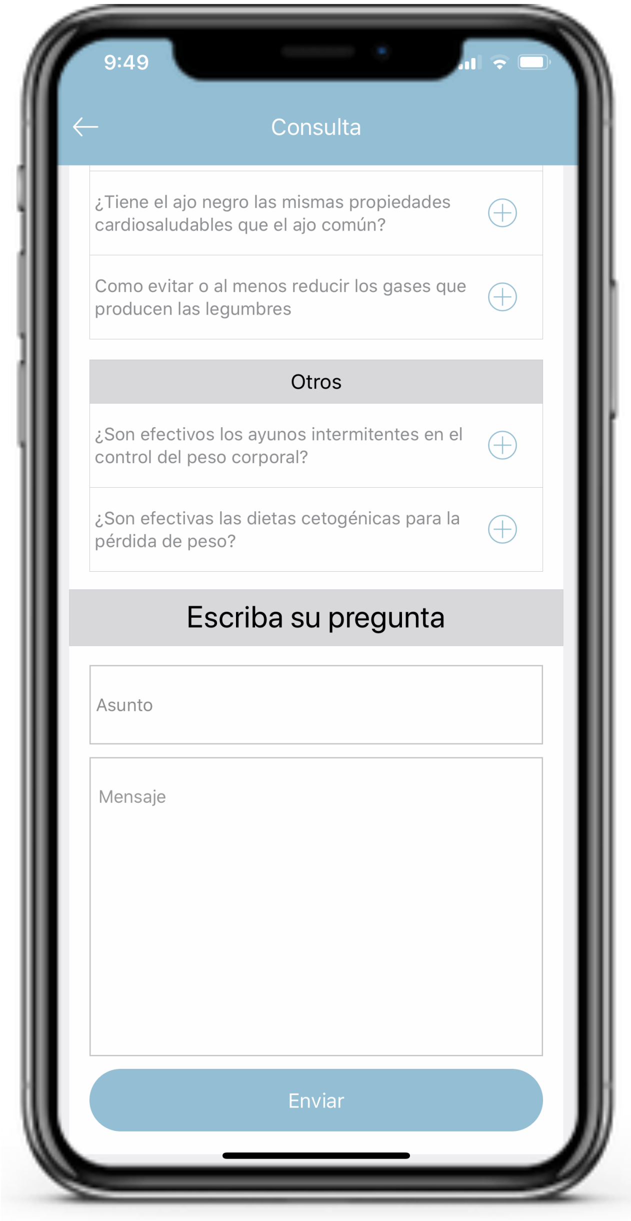

6. QUESTIONNAIRE OF ADHERENCE TO THE MEDITERRANEAN DIET

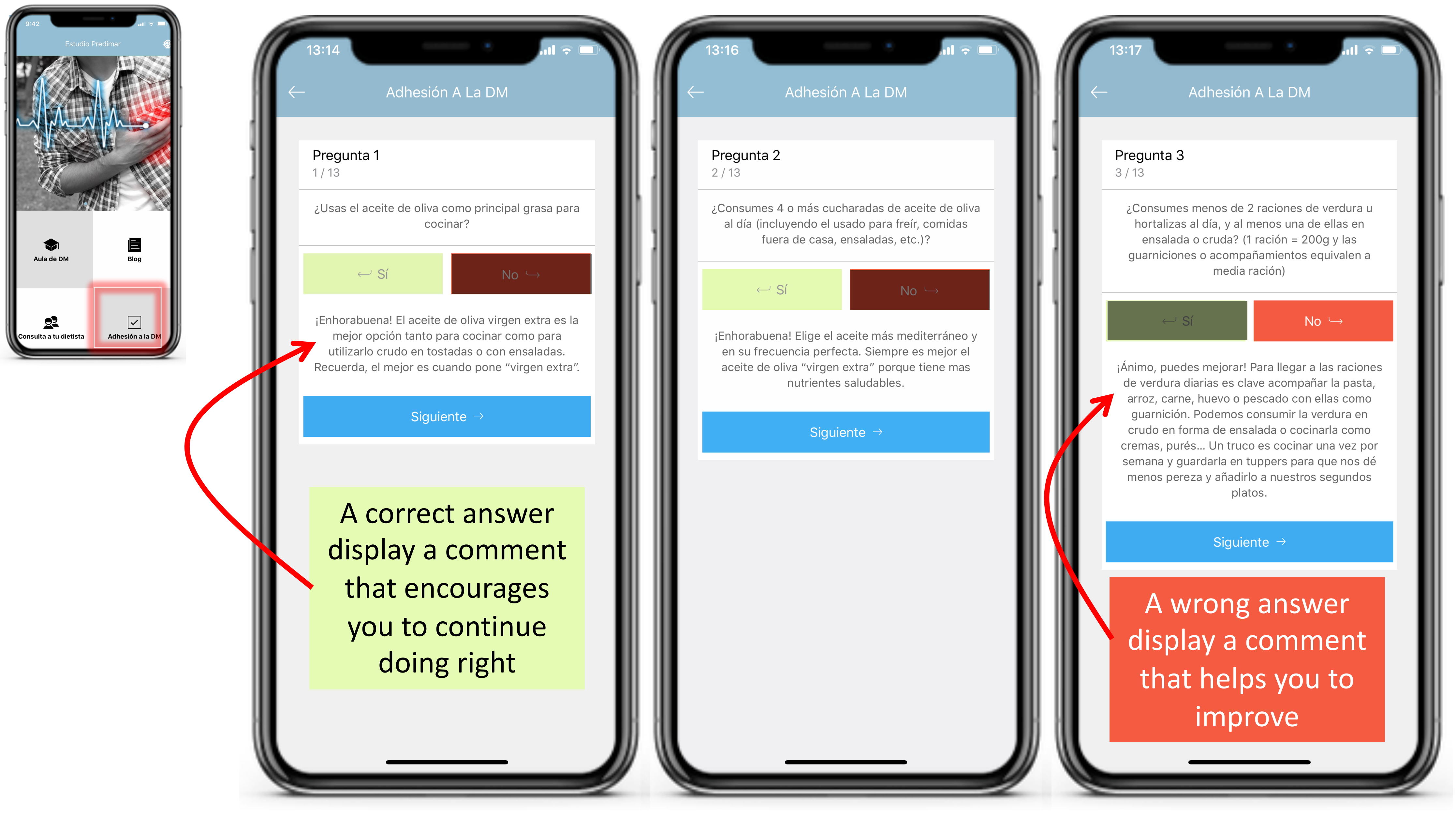

Supplement: Multimedia Appendix 4 [file jmir_v22i12e21436_app4.pdf]
