# Supplementary material for: A Remote Nutritional Intervention to Change the Dietary Habits of Patients Undergoing Ablation of Atrial Fibrillation: Randomized Controlled Trial
Source: J Med Internet Res. 2020 Dec 7;22(12):e21436. doi: 10.2196/21436 (PMC7752535; doi:10.2196/21436)
Supplement: Multimedia Appendix 5 [file jmir_v22i12e21436_app5.pdf]

MULTIMEDIA APPENDIX 5. FOOD FREQUENCY QUESTIONNAIRE

PREDIMAR

FRECUENCIA DE CONSUMO

marque así — *así no marque*

Nodo	Paciente	Visita	Fecha actual (Día / Mes / Año)	Número
				00002

1

Por favor, marque una única opción para cada alimento.

Para cada alimento, marque el recuadro que indica la frecuencia de consumo **por término medio** durante el **año pasado**. Se trata de tener en cuenta también la variación verano/invierno. Por ejemplo, si toma helados 4 veces/semana sólo durante los 3 meses de verano, el consumo promedio al año es 1/semana

I. LÁCTEOS

	CONSUMO MEDIO DURANTE EL AÑO PASADO									
	NUNCA O CASI NUNCA	AL MES	A LA SEMANA			AL DÍA				
		1 - 3	1	2 - 4	5 - 6	1	2 - 3	4 - 6	6 +
1. Leche entera (1 taza, 200 cc)									
2. Leche semidesnatada (1 taza, 200 cc)									
3. Leche descremada (1 taza, 200 cc)									
4. Leche condensada (1 cucharada)									
5. Nata o crema de leche (1/2 taza)									
6. Batidos de leche (1 vaso, 200 cc)									
7. Yogurt entero (1, 125 gr.)									
8. Yogurt descremado (1, 125 gr.)									
9. Petit suisse (1, 55 gr.)									
10. Requesón o cuajada (1/2 taza)									
11. Queso en porciones o cremoso (1, porción 25 gr.)									
12. Otros quesos: curados, semicurados (Manchego, Bola, Emmental…) (50 gr.)									
13. Queso blanco o fresco (Burgos, cabra…) (50 gr.)									
14. Natillas, flan, puding (1, 130 cc)									
15. Helados (1 cucurucho)									

II. HUEVOS, CARNES, PESCADOS

Un plato o ración de 100-150 gr., excepto cuando se indique otra cantidad	NUNCA O CASI NUNCA	AL MES	A LA SEMANA			AL DÍA			
		1 - 3	1	2 - 4	5 - 6	1	2 - 3	4 - 6	6 +
16. Huevos de gallina (uno)									
17. Pollo o pavo CON piel (1 ración o pieza)									
18. Pollo o pavo SIN piel (1 ración o pieza)									
19. Carne de ternera o vaca (1 ración)									
20. Carne de cerdo (1 ración)									
21. Carne de cordero (1 ración)									
22. Conejo o liebre (1 ración)									
23. Hígado (ternera, cerdo, pollo) (1 ración)									
24. Otras vísceras (sesos, corazón, mollejas) (1 ración)									
25. Jamón serrano o paletilla (1 loncha, 30 gr.)									
26. Jamón York, jamón cocido (1 loncha, 30 gr.)									
27. Carnes procesadas (salchichón, chorizo, morcilla, mortadela, salchichas, butifarra, sobrasada) (50 gr.)									
28. Patés, foie-gras (25 gr.)									
29. Hamburguesa (una, 50 gr.), albóndigas (3 unidades)									
30. Tocino, bacon, panceta (50 gr.)									
31. Pescado blanco: mero, lenguado, besugo, merluza, pescadilla,... (1 plato, pieza o ración)									
32. Pescado azul: sardinas, atún, bonito, caballa, salmón (1 plato, pieza o ración 130 gr.)									
33. Pescados salados: bacalao, salazones (1 ración, 60 gr. en seco)									
34. Ostras, almejas, mejillones y similares (6 unidades)									
35. Calamares, pulpo, chipirones, jibia (sepia) (1 ración, 200 gr.)									
36. Crustáceos: gambas, langostinos, cigalas, etc. (4-5 piezas, 200 gr.)									
37. Pescados y mariscos enlatados al natural (sardinas, anchoas, bonito, atún) (1 lata pequeña o media lata normal, 50 gr.)									
38. Pescados y mariscos en aceite (sardinas, anchoas, bonito, atún) (1 lata pequeña o media lata normal, 50 gr.)									

© Departamento de Medicina Preventiva UNIVERSIDAD DE NAVARRA

Por favor, marque una única opción para cada alimento.

Un plato o ración de 200 grs., excepto cuando se indique	CONSUMO MEDIO DURANTE EL AÑO PASADO								
	NUNCA O CASI NUNCA	AL MES	A LA SEMANA			AL DÍA			
		1 - 3	1	2 - 4	5 - 6	1	2 - 3	4 - 6	6 +
**III. VERDURAS Y HORTALIZAS**									
39. Acelgas, espinacas									
40. Col, coliflor, brócoles									
41. Lechuga, endivias, escarola (100 gr.)									
42. Tomate crudo (1, 150 gr.)									
43. Zanahoria, calabaza (100 gr.)									
44. Judías verdes									
45. Berenjenas, calabacines, pepinos									
46. Pimientos (150 gr.)									
47. Espárragos									
48. Gazpacho andaluz (1 vaso, 200 gr.)									
49. Otras verduras (alcachofa, puerro, cardo, apio)									
50. Cebolla (media unidad, 50 gr.)									
51. Ajo (1 diente)									
52. Perejil, tomillo, laurel, orégano, etc. (una pizca)									
53. Patatas fritas comerciales (1 bolsa, 50 gr.)									
54. Patatas fritas caseras (1 ración, 150 gr.)									
55. Patatas asadas o cocidas									
56. Setas, níscalos, champiñones									

Una pieza o ración	CONSUMO MEDIO DURANTE EL AÑO PASADO								
	NUNCA O CASI NUNCA	AL MES	A LA SEMANA			AL DÍA			
		1 - 3	1	2 - 4	5 - 6	1	2 - 3	4 - 6	6 +
**IV. FRUTAS**									
57. Naranja (una), pomelo (uno), o mandarinas (dos)									
58. Plátano (uno)									
59. Manzana o pera (una)									
60. Fresas/fresones (6 unidades, 1 plato postre)									
61. Cerezas, picotas, ciruelas (1 plato de postre)									
62. Melocotón, albaricoque, nectarina (una pieza)									
63. Sandía (1 tajada, 200-250 gr.)									
64. Melón (1 tajada, 200-250 gr.)									
65. Kiwi (1 unidad, 100 gr.)									
66. Uvas (un racimo, 1 plato postre)									
67. Aceitunas (10 unidades)									
68. Frutas en almíbar o en su jugo (2 unidades)									
69. Dátiles, higos secos, uvas-pasas, ciruelas-pasas (50 gr.)									
70. Almendras									
71. Pistachos									
72. Nueces									
73. Otros frutos secos									

74. ¿Cuántos días a la semana toma fruta como postre?	0	1	2	3	4	5	6	7

Un plato o ración	CONSUMO MEDIO DURANTE EL AÑO PASADO								
	NUNCA O CASI NUNCA	AL MES	A LA SEMANA			AL DÍA			
		1 - 3	1	2 - 4	5 - 6	1	2 - 3	4 - 6	6 +
**V. LEGUMBRES Y CEREALES**									
75. Lentejas (1 plato, 150 gr. cocidas)									
76. Alubias (pintas, blancas o negras) (1 plato, 150 gr. cocidas)									
77. Garbanzos (1 plato, 150 gr. cocidos)									
78. Guisantes, habas (1 plato, 150 gr. cocidas)									
79. Pan blanco, pan de molde (3 rodajas, 75 gr.)									
80. Pan negro o integral (3 rodajas, 75 gr.)									
81. Cereales desayuno (30 gr.)									
82. Cereales integrales: muesli, copos avena, all-bran (30 gr.)									
83. Arroz blanco (60 gr. en crudo)									
84. Arroz integral									
85. Pasta: fideos, macarrones, espaguetis, otras (60 gr. en crudo)									
86. Pasta integral									
87. Pizza (1 ración, 200 gr.)									

© Departamento de Medicina Preventiva UNIVERSIDAD DE NAVARRA

marque así *así no marque*

Número

Repita el número de la 1ª hoja
y vuelva a marcarlo

0	0	0	0	0
1	1	1	1	1
2	2	2	2	2
3	3	3	3	3
4	4	4	4	4
5	5	5	5	5
6	6	6	6	6
7	7	7	7	7
8	8	8	8	8
9	9	9	9	9

CONSUMO MEDIO DURANTE EL AÑO PASADO

¿Con que frecuencia consume?	NUNCA O CASI NUNCA	AL MES	A LA SEMANA			AL DÍA			
		1 - 3	1	2 - 4	5 - 6	1	2 - 3	4 - 6	6 +
88. Alimentos fritos en casa									
89. Alimentos fritos fuera de casa									

Por favor, marque una única opción para cada alimento.

CONSUMO MEDIO DURANTE EL AÑO PASADO

VI. ACEITES Y GRASAS

Una cucharada o porción individual. Para freír, untar, mojar en el pan, aliñar o para ensaladas, utiliza en total:	NUNCA O CASI NUNCA	AL MES	A LA SEMANA			AL DÍA			
		1 - 3	1	2 - 4	5 - 6	1	2 - 3	4 - 6	6 +
90. Aceite de oliva (una cucharada sopera)									
91. Aceite de oliva virgen (una cucharada sopera)									
92. Aceite de oliva de orujo (una cucharada sopera)									
93. Aceite de maíz (una cucharada sopera)									
94. Aceite de girasol (una cucharada sopera)									
95. Aceite de soja (una cucharada sopera)									
96. Mezcla de los anteriores (una cucharada sopera)									
97. Margarina (porción individual, 12 gr.)									
98. Mantequilla (porción individual, 12 gr.)									
99. Manteca de cerdo (10 gr.)									

100. Marca de aceite de oliva que usa habitualmente: ....

0	1	2	3	4	5	6	7	8	9
0	1	2	3	4	5	6	7	8	9

No marque aquí

VII. BOLLERÍA Y PASTELERÍA

CONSUMO MEDIO DURANTE EL AÑO PASADO	NUNCA O CASI NUNCA	AL MES	A LA SEMANA			AL DÍA			
		1 - 3	1	2 - 4	5 - 6	1	2 - 3	4 - 6	6 +
101. Galletas tipo María (4-6 unidades, 50 gr.)									
102. Galletas integrales o de fibra (4-6 unidades, 50 gr.)									
103. Galletas con chocolate (4 unidades, 50 gr.)									
104. Repostería y bizcochos hechos en casa (50 gr.)									
105. Croissant, ensaimada, pastas de té u otra bollería industrial comercial… (uno, 50 gr.)									
106. Donuts (uno)									
107. Magdalenas (1-2 unidades)									
108. Pasteles (uno, 50 gr.)									
109. Churros, porras y similares (1 ración, 100 gr.)									
110. Chocolates y bombones (30 gr.)									
111. Cacao en polvo-cacaos solubles (1 cucharada de postre)									
112. Turrón (1/8 de barra, 40 gr.)									
113. Mantecados, mazapán (90 gr.)									

VIII. MISCELÁNEA

CONSUMO MEDIO DURANTE EL AÑO PASADO	NUNCA O CASI NUNCA	AL MES	A LA SEMANA			AL DÍA			
		1 - 3	1	2 - 4	5 - 6	1	2 - 3	4 - 6	6 +
114. Croquetas, empanadillas, precocinados (una ración)									
115. Sopas y cremas de sobre (1 plato)									
116. Mostaza (una cucharadita de postre)									
117. Mayonesa comercial (1 cucharada sopera = 20 gr.)									
118. Salsa de tomate frito, ketchup (1 cucharadita)									
119. Picante: tabasco, pimienta, pimentón (una pizca)									
120. Sal (una pizca)									
121. Mermeladas (1 cucharadita)									
122. Azúcar (1 cucharadita)									
123. Miel (1 cucharadita)									
124. Snacks distintos de patatas fritas: gusanitos, palomitas, maíz, etc. (1 bolsa, 50 gr.)									
125. Otros alimentos de frecuente consumo:									
125.1									
125.2									
125.3									

SUMCO 000000-13 (2)

© Departamento de Medicina Preventiva UNIVERSIDAD DE NAVARRA

125. Otros alimentos de frecuente consumo (continuación)

125.1 (No marque aquí)
0 1 2 3 4 5 6 7 8 9
0 1 2 3 4 5 6 7 8 9

125.2 (No marque aquí)
0 1 2 3 4 5 6 7 8 9
0 1 2 3 4 5 6 7 8 9

125.3 (No marque aquí)
0 1 2 3 4 5 6 7 8 9
0 1 2 3 4 5 6 7 8 9

IX. BEBIDAS

Por favor, marque una única opción para cada alimento.

	CONSUMO MEDIO DURANTE EL AÑO PASADO								
	NUNCA O CASI NUNCA	AL MES	A LA SEMANA			AL DÍA			
		1 - 3	1	2 - 4	5 - 6	1	2 - 3	4 - 6	6 +
126. Bebidas carbonatadas con azúcar: bebidas con cola, limonadas, tónicas, etc. (1 botellín, 200 cc)									
127. Bebidas carbonatadas bajas en calorías, bebidas light (1 botellín, 200 cc)									
128. Zumo de naranja natural (1 vaso, 200 cc)									
129. Zumos naturales de otras frutas (1 vaso, 200 cc)									
130. Zumos de frutas en botella o enlatados (200 cc)									
131. Café descafeinado (1 taza, 50 cc)									
132. Café (1 taza, 50 cc)									
133. Té (1 taza, 50 cc)									
134. Mosto (100 cc)									
135. Vaso de vino rosado (100 cc)									
136. Vaso de vino moscatel (50 cc)									
137. Vaso de vino tinto joven, del año (100 cc)									
138. Vaso de vino tinto añejo (100 cc)									
139. Vaso de vino blanco (100 cc)									
140. Vaso de cava (100 cc)									
141. Cerveza (1 jarra, 330 cc)									
142. Licores, anís o anisetes… (1 copa, 50 cc)									
143. Destilados: whisky, vodka, ginebra, coñac (1 copa, 50 cc)									

144. ¿A que edad empezó a beber alcohol (vino, cerveza o licores), incluyendo el que toma con las comidas con regularidad (más de siete "bebidas" a la semana)?

Edad (años)
0 1 2 3 4 5 6 7 8 9 Decena
0 1 2 3 4 5 6 7 8 9 Unidad

145. ¿Cuántos años ha bebido alcohol con regularidad (más de siete "bebidas" a la semana)?

Años
0 1 2 3 4 5 6 7 8 9 Decena
0 1 2 3 4 5 6 7 8 9 Unidad

Si durante el año pasado tomó vitaminas y/o minerales (incluyendo calcio) o productos dietéticos especiales (salvado, aceite de onagra, leche con ácidos grasos omega-3, flavonoides, etc.), por favor indique la marca y la frecuencia con que los tomó:

Marcas de los suplementos de vitaminas o minerales o de los productos dietéticos	CONSUMO MEDIO DURANTE EL AÑO PASADO								
	NUNCA O CASI NUNCA	AL MES	A LA SEMANA			AL DÍA			
		1 - 3	1	2 - 4	5 - 6	1	2 - 3	4 - 6	6 +
146.									
146.1									
146.2									

146 (No marque aquí)
0 1 2 3 4 5 6 7 8 9
0 1 2 3 4 5 6 7 8 9

146.1 (No marque aquí)
0 1 2 3 4 5 6 7 8 9
0 1 2 3 4 5 6 7 8 9

146.2 (No marque aquí)
0 1 2 3 4 5 6 7 8 9
0 1 2 3 4 5 6 7 8 9

SUMCO 000000-13 2 Ret.

Muchas gracias por su colaboración

© Departamento de Medicina Preventiva UNIVERSIDAD DE NAVARRA
