# Supplementary material for: A Remote Nutritional Intervention to Change the Dietary Habits of Patients Undergoing Ablation of Atrial Fibrillation: Randomized Controlled Trial
Source: J Med Internet Res. 2020 Dec 7;22(12):e21436. doi: 10.2196/21436 (PMC7752535; doi:10.2196/21436)

MULTIMEDIA APPENDIX 6. ADHERENCE TO THE MEDITERRANEAN DIET AT BASELINE, AFTER 12- AND 24-MONTH FOLLOW-UP VISITS ACCORDING TO INTERVENTION GROUPS

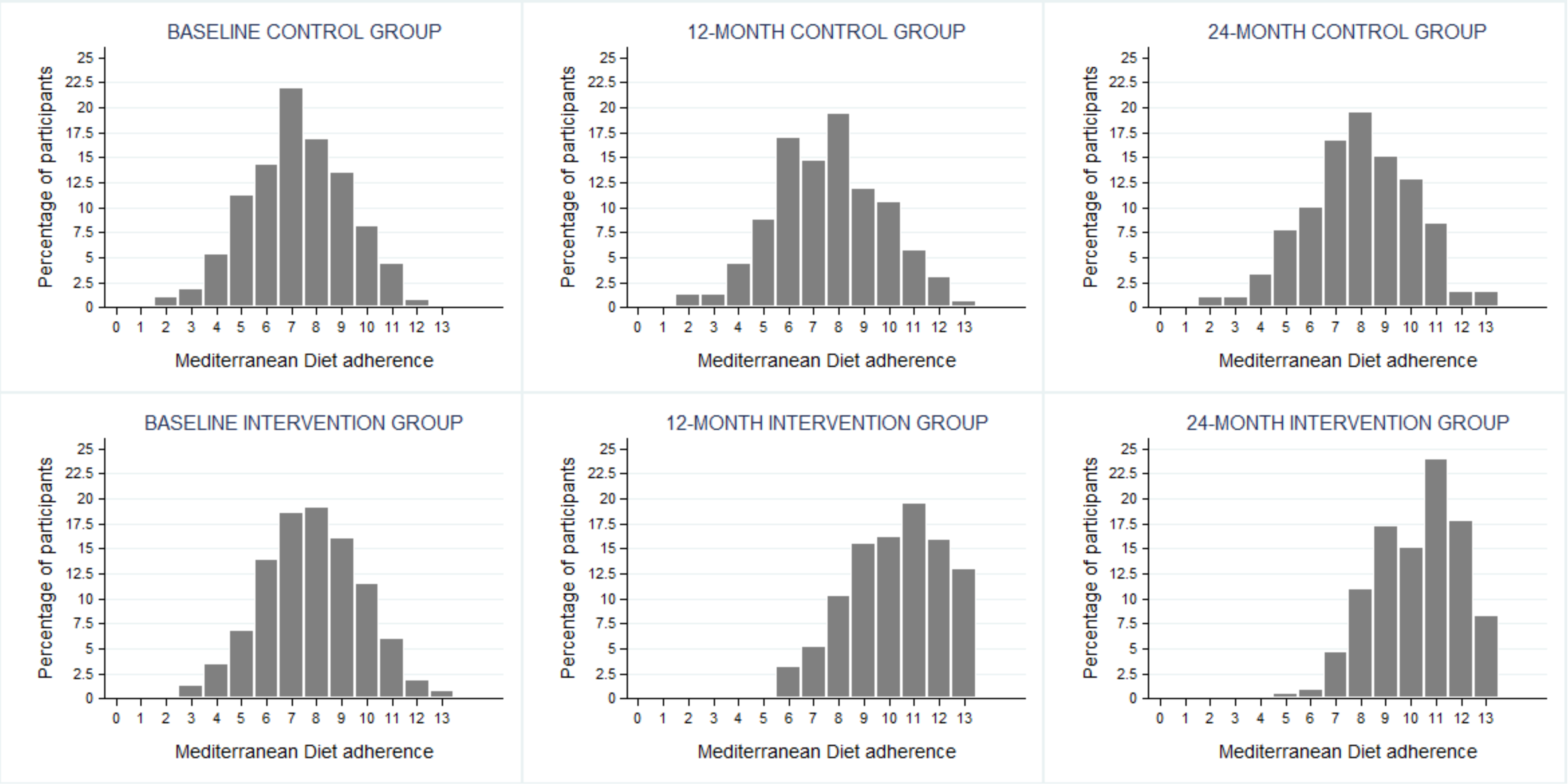

Supplement: Multimedia Appendix 6 [file jmir_v22i12e21436_app6.pdf]
